# Unravelling a stearidonic acid-rich triacylglycerol biosynthetic pathway in the developing seeds of *Buglossoides arvensis*: A transcriptomic landscape

**DOI:** 10.1038/s41598-017-09882-y

**Published:** 2017-09-05

**Authors:** R. V. Sreedhar, P. Prasad, L. Prasanna Anjaneya Reddy, Ram Rajasekharan, Malathi Srinivasan

**Affiliations:** 1 Department of Lipid Science, CSIR-Central Food Technological Research Institute (CSIR-CFTRI), Mysuru, 570020 India; 2 Academy of Scientific and Innovative Research (AcSIR), CSIR-Central Food Technological Research Institute Campus, Mysuru, 570020 India

## Abstract

*Buglossoides arvensis* is an emerging oilseed crop that is rich in stearidonic acid (SDA) and has several potential applications in human health and nutrition. The molecular basis of SDA biosynthesis in this plant remains unknown due to lack of genomic information. To unravel key genes involved in SDA-rich triacylglycerol (TAG) biosynthesis, we performed transcriptome sequencing of pooled mRNA from five different developmental stages of *B*. *arvensis* seeds using Illumina NextSeq platform. *De novo* transcriptome assembly generated 102,888 clustered transcripts from 39.83 million high-quality reads. Of these, 62.1% and 55.54% of transcripts were functionally annotated using Uniprot-Viridiplantae and KOG databases, respectively. A total of 10,021 SSR-containing sequences were identified using the MISA tool. Deep mining of transcriptome assembly using *in silico* tools led to the identification of genes involved in fatty acid and TAG biosynthesis. Expression profiling of 17 key transcripts involved in fatty acid desaturation and TAG biosynthesis showed expression patterns specific to the development stage that positively correlated with polyunsaturated fatty acid accumulation in the developing seeds. This first comprehensive transcriptome analysis provides the basis for future research on understanding molecular mechanisms of SDA-rich TAG accumulation in *B*. *arvensis* and aids in biotechnological production of SDA in other oilseed crops.

## Introduction

Long-chain ω-3 polyunsaturated fatty acids (LC ω-3 PUFA) are potent biological regulators with therapeutic and preventive properties for chronic diseases. Major available sources of LC ω-3 PUFA include marine foods (fish and microalgae) and plant seed oils^1, 2^. Concerns regarding sustainable supply, safety, taste, fishy odour and the non-vegetarian nature of marine sources have constrained their utilization^3^. Although terrestrial plant sources rich in alpha linolenic acid (ALA; 18:3 n-3), specifically, flax, chia, perilla, hemp and sacha inchi are safe, sustainable and scalable, but the efficiency of conversion of ALA to eicosapentaenoic acid (EPA; 20:5 n-3) in humans is limited due to the rate-limiting step catalysed by delta-6 desaturase (D6D)^4^. Therefore, higher proportions of ALA-rich plant oils need to be consumed to meet the recommended long chain ω-3 levels, which makes their utilization less efficient^5^.

*Buglossoides arvensis* (L.) I. M. Johnston, commonly called Corn Gromwell or Field Gromwell, is an annual weed of the Boraginaceae family and is native to the northern temperate regions of Asia and Europe^4^. The plant has gained attention recently in ω-3 fatty acid research because of its natural ability to synthesize and accumulate a non-traditional fatty acid, stearidonic acid (SDA), in its seed. SDA is an 18 carbon ω-3 PUFA with four *cis* double bonds in the acyl chain. Mounting evidence in humans and animal models indicate that consumption of SDA-rich plant oils raises tissue EPA levels more efficiently (2.2–4 times) than ALA rich plant oils because it bypasses the D6D rate-limiting step^4, 6–8^. The oil content of *B*. *arvensis* is approximately 19–21% (w/w) and is the richest natural source of SDA, which comprises 17–21% of total seed fatty acids. In addition to SDA, seed oil also contains 42–48% ALA and 4.5–8% γ-linolenic acid (GLA)^4^. Furthermore, seed oil has ~80–90% PUFA with a ω-6/ω-3 ratio of less than 1. The United States (US) FDA affirms self-determined GRAS (Generally Recognised as Safe) status for *B*. *arvensis* seed oil. The European Union has granted novel food status for the oil and approved daily intake levels in dietary supplements up to 500 mg SDA/day or 2.5 g of oil/day^4, 9^. Although other plant sources of SDA, such as *Ribes nigrum* (2–4% SDA) and *Echium* (12–14% SDA), are commercially available for human consumption, barriers associated with respect to their SDA content and seed yields obstruct their commercial exploitation^7^. Monsanto has developed a transgenic SDA-rich soybean crop (21–30% SDA) by introducing *Primula juliae* delta-6 desaturase and *Neurospora crassa fad3* (delta-15 desaturase) genes under the control of a seed-specific promoter^10^. However, its usage and market potential in genetically modified organism (GMO) free countries is still questionable. Given all these factors, improving the *B*. *arvensis* crop with a high seed yield, oil content and quality will be a promising strategy for meeting the increasing ω-3 PUFA demand.

Mostly, plant species that accumulate non-traditional fatty acids are wild and are not agronomically adapted. Currently, *B*. *arvensis* cultivars with a high seed yield and SDA content are developed and commercially cultivated in the UK^4^. Our group successfully assessed the adaptability and suitability of a *B*. *arvensis* wild accession (collected from the north temperate regions of India) for large scale commercial cultivation in the tropical regions of South India. We are currently in the process of developing high yielding lines with improved oil and SDA content through classical and modern molecular plant breeding techniques. However, the paucity of complete genetic data in *B*. arvensis has made the genomics-assisted breeding programme a challenging task.

In plants, the oil biosynthesis pathway is well known and mainly follows the pathways for fatty acid biosynthesis in plastids and TAG assembly in the endoplasmic reticulum (ER)^11^. Fatty acids synthesised in the plastids (oleic acid > palmitic acid > stearic acid) are exported to the acyl-CoA pool for incorporation into membrane phosphatidylcholine (PC) through acyl-editing reactions or into TAG through acyl-CoA dependent reactions^12–14^. Furthermore, TAG can also be synthesised through acyl-CoA independent reactions involving the transfer of the acyl group from PC to DAG^15^. PC is the substrate for fatty acid modifying enzymes, such as desaturases. Therefore, acyl flux into TAG by PC acyl editing reactions or PC-DAG interconversion or acyl Co-A independent reaction greatly influence the fatty acid composition. Oil accumulation during seed development shows great diversity in TAG structure and the rate of oil synthesis^16^. Molecular mechanisms underlying non-traditional fatty acid (SDA and GLA) biosynthesis and accumulation in *B*. *arvensis* seed triacylglycerols are still unknown. Therefore, elucidating the *B*. *arvensis* lipid biosynthesis pathway using transcriptomic approach may provide a better insight into the process by unravelling the key lipid genes in addition to assisting with crop improvement. Previously, our group explored the TAG biosynthesis pathway in the developing seeds of ALA-rich *Salvia hispanica* (Chia) using a similar approach^17^.

With the advent of high throughput next generation sequencing (NGS) technologies, *denovo* transcriptome sequencing and assembly have become a fast and inexpensive tool to identify key genes and molecular markers in non-model crops or orphan crops that lack a reference genome^18^. In this study, we used an Illumina NextSeq. 500 platform aimed at establishing *B*. *arvensis* seed transcriptome data. We further characterized the temporal expression profiles of key lipid biosynthetic genes in five different developing stages of seeds and mature leaves along with their fatty acid profiles. Our data provide an insight for understanding non-traditional fatty acid synthesis and accumulation during seed development in *B*. *arvensis* that can be applied to improve its seed nutritional quality and quantity through molecular plant breeding programmes. Additionally, our data also helps in identifying key transcripts that can be exploited through transgenic technology for reprogramming fatty acid and oil biosynthesis in traditional oilseed crops.

## Results and Discussion

### Profiling of total lipid and fatty acid composition in *B*. *arvensis* developing seeds

**Figure 1:**
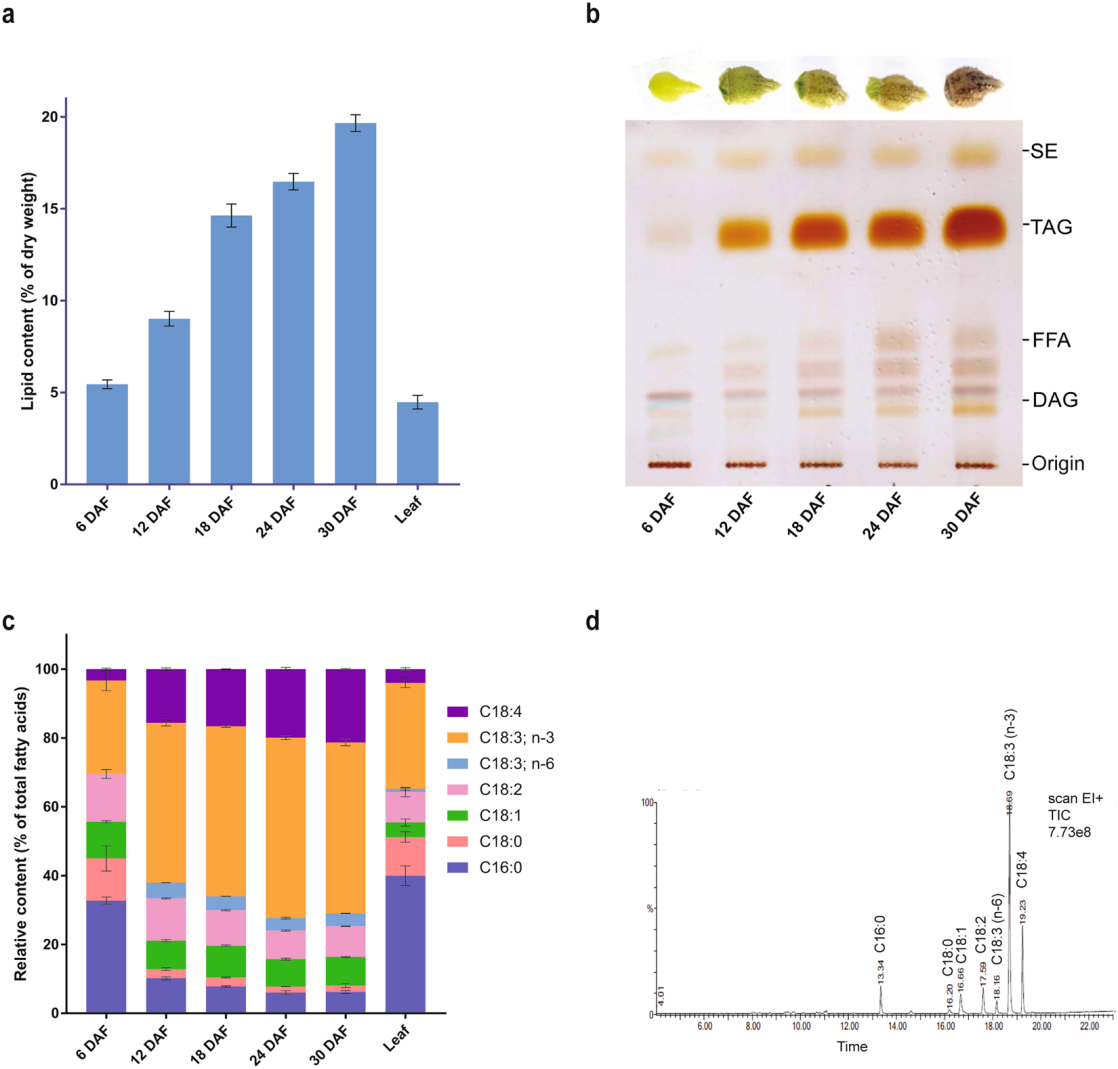
Lipid content and fatty acid composition. (**a**) Lipid content of *B*. *arvensis* developing seeds and leaves. Lipid content was determined gravimetrically and expressed as % of dry weight. (**b**) Neutral lipid profile of five developmental stages of *B*. *arvensis* seed by TLC. Lipids were extracted and separated on a silica-TLC plate using the solvent system, petroleum ether:diethyl ether:acetic acid (70:30:1, v/v) and developed by manganese chloride charring. (**c**) Fatty acid composition of developing seeds and leaves. (**d**) Representative GC-MS chromatogram showing the fatty acid profile of *B*. *arvensis* seeds (24 DAF). All values are represented as the mean of biological triplicates ± SD. DAF, days after flowering; SE, steryl ester; TAG, triacylglycerol; DAG, diacylglycerol; FFA, free fatty acid; C16:0, palmitic acid; C18:0, stearic acid; C18:1, oleic acid; C18:2, linoleic acid; C18:3 (n-6), gamma-linolenic acid; C18:3 (n-3), alpha-linolenic acid; C18:4, stearidonic acid.

Accumulation patterns of oil and fatty acids during five different developing stages of *B*. *arvensis* seeds (6, 12, 18, 24, 30 days after flowering) were studied by analysing their total lipid content and fatty acid composition. During seed development, total lipid accumulation paralleled seed maturation starting with a very low lipid content of 5.4% (of seed dry weight) at 6 days after flowering (DAF) and increased steadily to 9% at 12 DAF, 14.6% at 18 DAF, 16.4% at 24 DAF and 19.6% at 30 DAF (Fig. 1a). After 30 DAF, no significant changes in oil accumulation were observed, which suggested that seed development and maturation are completed in approximately 30 DAF. Lipid content of the leaf was also quantified and found to be 4.4% of leaf dry weight. Neutral lipid profiling of five different development stages of seed by thin layer chromatography showed a sharp increase in the triacylglycerol levels between 6 DAF and 12 DAF (Fig. 1b). Later, the TAG content increased gradually and reached its maximum level at 30 DAF, which suggested that TAG biosynthesis usually occurs at a high rate after 6 DAF and progresses until 30 DAF. A similar pattern of oil accumulation has been reported for a number of oilseed crops, such as *Arabidopsis* ^19^, *Brassica* ^20^, *Perilla* ^21^ and rapeseed^22^.

The fatty acid compositions of developing seeds and mature leaves were analysed by GC-MS (Fig. 1c,d and Supplementary Fig. S1). At 6 DAF, the relative amount of saturated fatty acids, such as palmitic acid (PA;16:0) and stearic acid (SA; 18:0), were about 32.7% and 12.27% of total fatty acids, respectively. The relative amounts of unsaturated fatty acids such as oleic acid (OA; 18:1), linoleic acid (LA; 18:2), α-linolenic acid (ALA; 18:3 n-3) and stearidonic acid (SDA; 18:4) were found to be 10.6%. 13.8%, 27.1% and 3.2% of total fatty acids, respectively. At 12 DAF, the fatty acid composition changed drastically with a marked decrease in the levels of 16:0, 18:0 and 18:1, whereas the levels of 18:2, 18:3 and 18:4 increased rapidly. Moreover, the levels of γ-linolenic acid (GLA; 18:3 n-6), which were not observed at 6 DAF were found to be 4.5% at 12 DAF, and later its level decreased to 3.5% at 24 DAF. From 12 DAF to 24 DAF, the levels of 16:0, 18:0 and 18:1 were decreased to 5.9%, 1.7% and 8.0%, respectively whereas the relative amounts of ALA and SDA increased to 52.0% and 19.9%, respectively. Mature seeds at 30 DAF contained 21.0% of SDA, 49.6% of ALA, 3.7% of GLA, 8.9% of LA, 8.2% of OA, 1.9% of SA and 6.1% of PA. In leaf samples, 3.9% of SDA and 30.8% of ALA were detected, which was almost comparable to the ALA and SDA content of the 6 DAF seed samples. A similar trend in fatty acid composition during seed development reflected a gradual transition from the early stage with more saturated fatty acids to a mature stage with more unsaturated fatty acids, which were observed in *Arabidopsis thaliana* ecotype WS^23^ and *Perilla* ^21^.

Overall, it is evident from these data that oil and PUFA accumulate in a stage-specific manner during seed development. Therefore, to explore all the transcripts involved in oil and PUFA synthesis, we used RNA samples pooled from five different stages of developing seeds (6, 12, 24, 18 and 30 DAF) for transcriptome sequencing and analysis.

### Transcriptome sequencing of *B*. *arvensis* developing seeds and its *de novo* assembly

RNA-seq, a promising NGS technology, has been widely used in plant functional genomics studies. It provides insights into the gene content, identification of transcripts of interest and molecular markers in non-model plants with no genomic information or sequencing data in public databases^24^. To obtain *B*. *arvensis* transcriptome data, a normalized pair end cDNA library constructed by pooling total RNA from five different developing stages of seeds was sequenced with the Illumina NextSeq 500 platform. Sequencing pooled sample decreases the frequency of highly expressed transcripts and increases the frequency of unique or rare transcripts^25, 26^. A total of 42,528,726 raw paired end sequence reads, each 150 bp in length, were generated in a FASTQ file with a size of 8 Gb. After stringent quality checks and processing raw reads to remove low quality bases and adapter trimming, 39,833,196 high-quality processed reads with a phred quality score greater than 30 were selected for downstream analysis. The Q30 percentage was 82.24% and the GC content was 44%, which suggested there was high accuracy and reliability for the transcriptome sequencing. The raw data statistics are shown in Table 1. Raw data in FASTQ format was submitted to the National Centre for Biotechnology Information (NCBI) Sequence Reads Archive (SRA) under the BioProject accession number PRJNA344425.

**Table 1:** Transcriptome raw data and *de novo* assembly statistics of *B*. *arvensis*.

Summary of sequencing reads		
Number of raw reads	42528726	
Number of processed reads	39833196	
Total number of nucleotides	6379308900	
GC content (%)	44%	
Q30 (%)	82.24%	
Summary of *de novo* assembly		
**Tool used**	**Trinity (Transcripts)**	**Trinity + Cd- hit (Clustered transcripts)**
Hash length	25	25
Transcripts generated	1,20,320	1,02,888
Maximum transcript length	16659	16659
Minimum transcript length	301	301
Average transcript length	954.6	922.8
Median Transcript Length	823	450
Total Transcripts Length	11,48,54,624	9,49,41,727
Total Number of Non-ATGC Characters	0	0
Transcripts >= 300 b	1,20,320	1,02,888
Transcripts >= 500 b	77640	64959
Transcripts >= 1 Kb	40411	32509
Transcripts >= 5 Kb	335	259
Transcripts >= 10 Kb	10	9
N50 value	1318	1263
GC%	39.13252809	38.94526376

Due to the lack of a reference genome for *B*. *arvensis*, *de novo* assembly was carried out using the Trinity assembler with a hash length of 25. A total of 120,320 transcripts were generated with minimum and maximum transcript lengths of 301 bp and 16,659 bp, respectively. The mean length of the transcripts was 954.6 bp and the N50 length was 1318 bp, which was high comparable to the length reported in other species of the Boraginaceae family, such as the *Echium wildpretii* seedling transcriptome (mean length = 707; N50 = 1041)^27^ and *Lithospermum multiflorum* floral transcriptome (mean length = 691; N50 = 1201)^28^. The GC content of the assembled transcripts was 39.13%, which was similar to the *L*. *multiflorum* transcriptome GC content (38.53%) and indicated an evolutionary relationship between the species within the family^28^. The final transcripts were clustered *via* CD-HIT using a 90% identity cut-off and a total of 102,888 clustered transcripts were generated with a mean transcript length of 922.8 bp and an N50 length of 1263 bp. *De novo* assembly statistics are shown in Table 1.

Deep sequencing coverage and the quality of the transcriptome assembly evident from parameters such as the mean transcript length, N50 value and GC content in the present study not only provide the first reference transcriptome data but also assist in elucidating SDA and TAG biosynthesis pathways as well as conducting subsequent genomics-based plant breeding studies in *B*. *arvensis*.

### Functional annotation and classification of *B*. *arvensis* transcriptome

**Figure 2:**
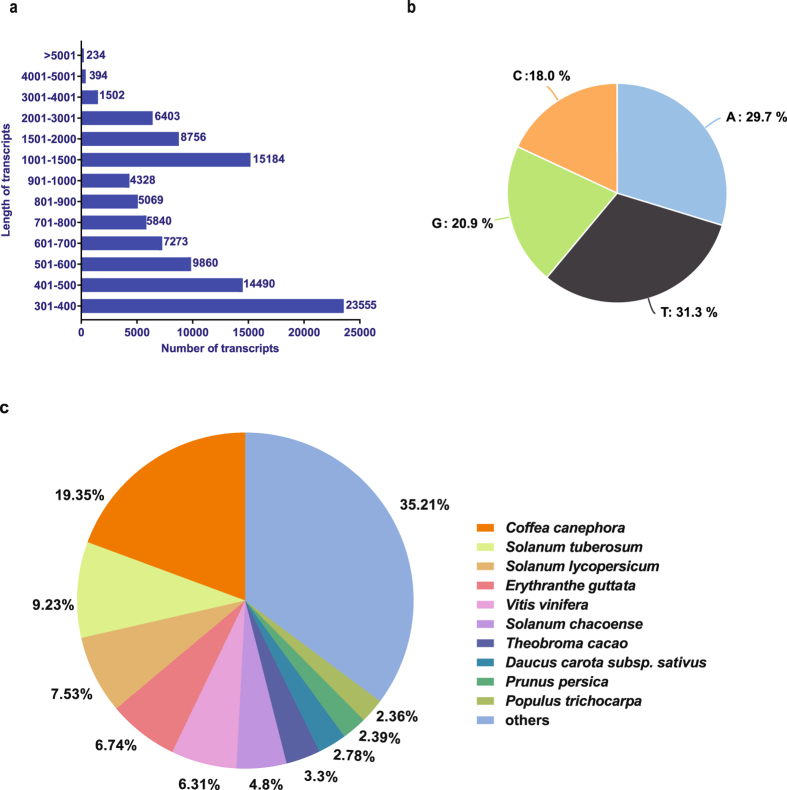
Annotation statistics of *B*. *arvensis* clustered transcripts against Uniprot-Viridiplantae database. (**a**) Length distribution of clustered transcripts. (**b**) Nucleotide content of clustered transcripts. (**c**) Species distribution of the top BLASTX hits in the Uniprot database.

To predict the molecular functions of *de novo* assembled transcripts, functional annotation was performed using a BLASTX homology search against the Uniprot-Viridiplantae database. A total of 63,823 (62.1%) transcripts were functionally annotated with an e-value of ≤10^–5^, whereas 39,065 (37.9%) transcripts were not annotated, which might represent putative unique *B*. *arvensis* transcripts, non-coding RNAs or short transcripts with no conserved protein domains. Analysis of *B*. *arvensis* clustered transcripts length distribution indicated that 32,473 transcripts had a length >1000 bp, of these 29,897 transcripts showed homology to Uniprot-Viridiplantae database and majority of which could be full length (9331 transcripts showed ≥75% homology) (Supplementary Table S1). Length distribution and nucleotide content of the clustered transcripts are shown in Fig. 2a and b. Species distribution of annotated transcripts showed maximum homology to *Coffea canephora* (19.35%) followed by *Solanum tuberosum* (9.23%) and *Solanum lycopersicum* (7.53%) (Fig. 2c).

**Figure 3:**
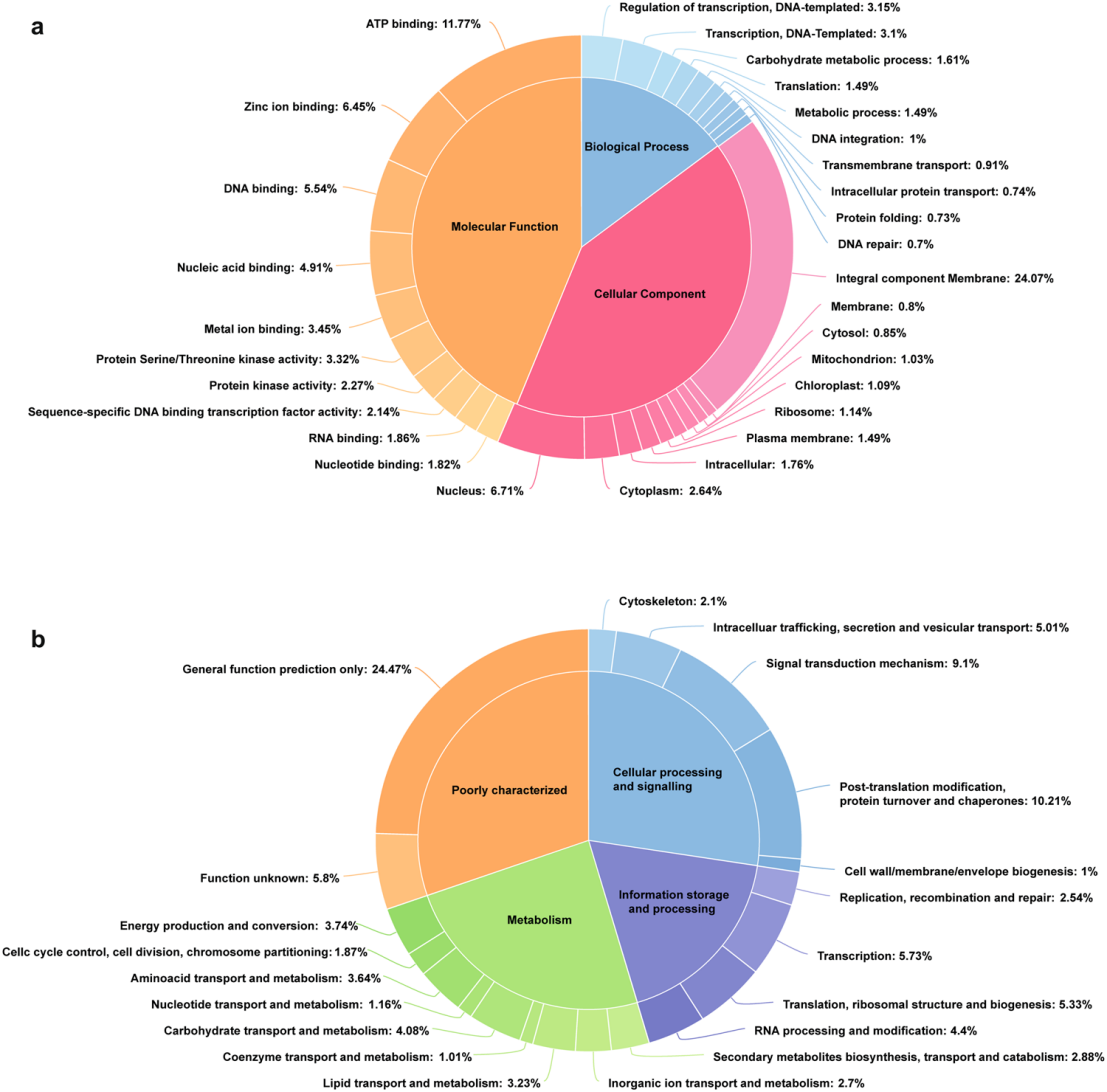
GO and KOG classification. (**a**) Gene ontology distribution of *B*. *arvensis* clustered transcripts according to biological process, cellular component and molecular function. The percentage of transcripts encoding the top ten GO terms in each category is represented. (**b**) KOG classification of clustered transcripts into metabolism, cellular processes and signalling, poorly characterized and information storage and processing. The percentage of clustered transcripts encoding the KOG terms in each category are represented.

Transcripts annotated against the Uniprot database were functionally categorized using Gene Ontology (GO) terms (Fig. 3a and Supplementary Data 1). In total, 44,938 transcripts were assigned with at least one GO term and were further categorized into three major functional ontologies: biological process (20,697 transcripts), molecular function (34,983 transcripts) and cellular component (24,470 transcripts). In the biological process category, regulation of transcription was the most represented group followed by transcription and the carbohydrate metabolic process. Under the molecular function category, ATP binding was a highly represented group followed by zinc ion binding and DNA binding. Among the cellular component category, the integral component membrane was the predominant group followed by the nucleus and cytoplasm.

Clustered transcripts were also annotated against the KOG database for function prediction and classification (Fig. 3b and Supplementary Data 2). In total, 57,146 transcripts (55.54%) were annotated, of which 35,338 (34.3%) transcripts were grouped into four functional categories: cellular processing and signalling (9,402 transcripts), information storage and processing (5,820 transcripts), metabolism (6,993 transcripts) and poorly characterized (9,433 transcripts). In the metabolism category, carbohydrate transport and metabolism class were highly represented followed by energy production and conversion, amino acid and lipid metabolism and transport. Developing seeds accumulate carbohydrates and lipids at high rate^17^, which is evident from the transcripts related to those metabolisms that are prominently represented in the KOG classification. In the information storage and processing category, the transcription class is the most predominant, whereas in the cellular processing and signalling category, the post-translation modification, protein turnover and chaperone classes were the most represented. However, poorly characterized category with general function prediction and function unknown classes were accounted for large fraction of transcripts, suggesting distant relation of *B*. *arvensis* to the organisms comprising the KOG database. Overall, functional annotation analysis of *B*. *arvensis* developing seeds transcriptome provides comprehensive data about the expressed transcripts involved in specific processes, which helps with translational research for crop improvement.

### Pathway analysis using the Kyoto Encyclopedia of Genes and Genomes (KEGG)

**Figure 4:**
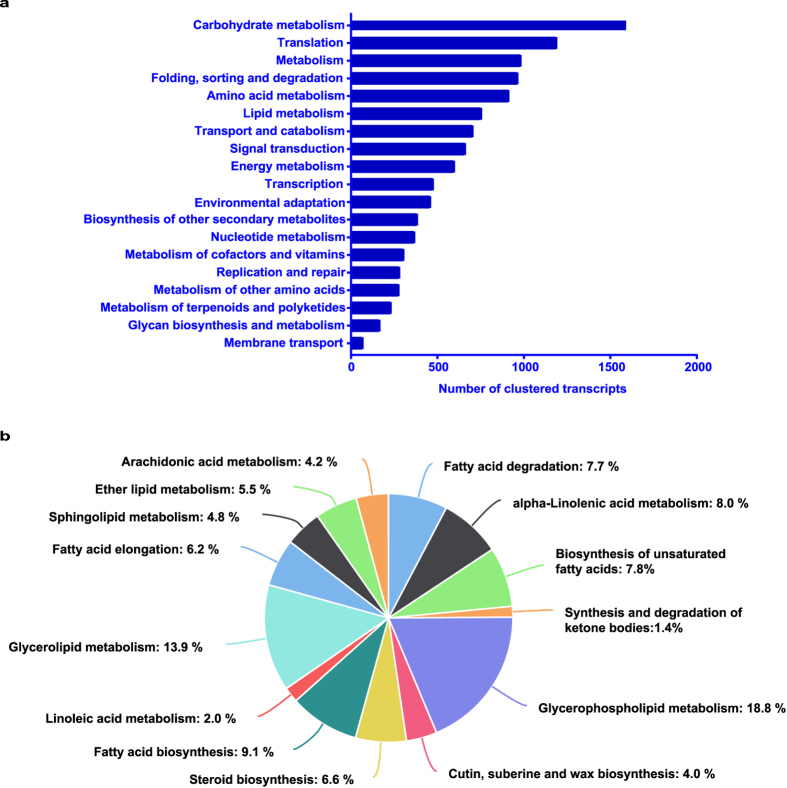
KEGG pathway analysis. (**a**) Classification of *B*. *arvensis* clustered transcripts based on the metabolism category. The bars represent the number of clustered transcripts in each sub-category of metabolism. (**b**) Percentage distribution of lipid metabolism transcripts.

Pathway analysis using the KEGG automatic annotation server (KAAS) provides a systematic understanding of the biological functions and molecular interactions between genes. Biological pathways active in *B*. *arvensis* were identified by annotating all of the clustered transcripts with KO identifiers in KEGG database using BLASTX and the bi-directional best hit method. A total of 11,439 transcripts (11.12%) showed significant hits in the KEGG database and were categorized into 19 pathway functions (Fig. 4a and Supplementary Data 3). The most represented pathway functions include carbohydrate metabolism, translation, metabolism, folding, sorting and degradation, amino acid metabolism and lipid metabolism. To further unravel the pathways in relation to lipid metabolism, we categorized transcripts involved in lipid metabolism into 14 sub-categories (Fig. 4b and Supplementary Data 3). The predominant pathways in lipid metabolism include glycerophospholipid metabolism (18.8%), glycerolipid metabolism (13.9%), fatty acid biosynthesis (9.1%), α-linolenic acid metabolism (8.0%) and biosynthesis of unsaturated fatty acids (7.8%). The KEGG Pathway analysis provides more targeted information on gene products involved in various pathways that will guide future research in *B*. *arvensis*.

### Identification of transcription factors and simple sequence repeats

Transcription Factors (TFs) play an important role in modulating the transcriptional behaviour of genes involved in plant growth and development^29^. We identified a total of 28,973 TF-encoding transcripts belonging to 70 different TF families through a homology search against the Plant TFDB (Transcription factor database). Identified TFs were mapped to KEGG pathways and a total of 3,101 transcripts were found to be involved in various KEGG pathway functions (Supplementary Data 4). Furthermore, we also identified a total of 176 transcripts mapped to lipid metabolism, of which TFs belonging to NAC, MYB-related, C3H, bHLH, ARF, MYB and G2 like families were more predominant (Supplementary Fig. S2). TFs belonging to MYB-related, bHLH, B3, GRAS, MYB and G2-like families play major roles in the regulation of fatty acid and oil biosynthesis^30^. Many transcription factors such as LEAFY COTYLEDON 1 (LEC1), FUSCA 3 (FUS3), WRINKLED 1 (WRI1), HIGH LEVEL EXPRESSION OF SUCROSE INDUCIBLE GENE (HSI2 and HSI1), PICKLE (PKL), GLABRA 2 (GL2) and APETALA 2 (AP2) are known to be master regulators of seed oil synthesis and deposition^16^. Putative transcripts encoding these TFs were identified from our transcriptome data (Supplementary Table S2), which could be potential candidates for metabolic engineering to improve the quality and quantity of oil in *B*. *arvensis*.

Simple sequence repeats (SSRs) are molecular markers with tandem repeats of 2–6 nucleotide bases. Transcriptome sequencing is a powerful and cost-effective tool to identify SSRs compared to whole genome sequencing because it targets genomic regions with corresponding transcribed gene sequences^31^. In the current study, we identified a total of 11,271 SSRs and 10,021 SSR-containing sequences, of which 1096 sequences have more than one SSR (Supplementary Data 5). The most predominant SSRs were mononucleotide repeats (4,658) followed by trinucleotide (3,987), dinucleotide (2,498), tetranucleotide (101), pentanucleotide (14) and hexanucleotide repeats (13). SSRs statistics are provided in Supplementary Table S3. Furthermore, we also identified 51 SSR-containing transcripts involved in lipid metabolism using KEGG pathway analysis. Among these, 16 and 8 SSR containing transcripts were found to be involved in fatty acid and TAG biosynthesis, respectively (Supplementary Data 5). With no molecular marker information available for *B*. *arvensis* in public databases, genic SSRs identified in our transcriptome data will be important genomic resources and valuable data for genetic map construction, germplasm evaluation and molecular marker-assisted breeding for this crop.

### Validation of transcriptome assembly

*De novo* transcriptome assembly using a Trinity assembler was validated by PCR amplification of 25 randomly selected genes (transcripts), namely, formate Dehydrogenase (FDH; 1141 bp), uricase (URI; 938 bp), class I glutamine amidotransferase-like superfamily protein (GAT; 1208 bp), adenosine kinase 2 (ADK2; 1223 bp), monodehydroascorbate reductase 1 (MDAR1; 1338 bp), NAD(P)-binding Rossmann-fold superfamily protein (FLDH; 1085 bp), 4-coumarate:CoA ligase 3 (4CL3; 1846 bp), pantoate-beta-alanine ligase (PANC; 1040 bp), proliferating cell nuclear antigen 2 (PCNA2; 894 bp), aldolase superfamily protein (ALD; 1224 bp), adenylate kinase 1 (ADK 1; 837 bp), cytosolic NADP+-dependent isocitrate dehydrogenase (clCDH; 1218 bp), ascorbate peroxidase 3 (APX3; 1070 bp), ras-related small GTP-binding family protein (RAS; 706 bp), squalene synthase 1 (SQS1; 1348 bp), glyceraldehyde-3-phosphate dehydrogenase C subunit 1 (GAPC; 1329 bp), rubisco activase (RCA; 1453 bp), Actin-11 (ACT11; 1235 bp), alpha tubulin (AT; 1360 bp), diacylglycerol acyltransferase 2 (DGAT2; 1111 bp), hydroxysteroid dehydrogenase 1 (HSD1; 1164 bp), fatty acid desaturase 2 (FAD2; 1291 bp), caleosin (CAL; 886 bp), delta-6 desaturase (D6D; 1350 bp) and oleosin (OLE; 441 bp). Agarose gel electrophoresis of PCR-amplified products showed amplicons of the expected size for the respective genes, which validated the transcriptome assembly (Supplementary Fig. S3a and b). To further confirm the sequence identity of assembled transcripts, we have sequenced all the above PCR amplified products using Sanger sequencing platform and found 99–100% sequence identity with the respective assembled transcript sequences (Supplementary Data 6).

### Identification of acyl-lipid transcripts involved in fatty acid (FA) biosynthesis

*B*. *arvensis* seeds have several nutraceutical and industrial application due to the natural ability to synthesize and accumulate unsaturated fatty acids (especially SDA) in their oil. To identify transcripts involved in FA biosynthesis, we mined the transcriptome assembly using tBLASTN with protein sequences encoding *Arabidopsis* FA biosynthesis genes^14^ as queries. All the major enzymes involved in fatty acid biosynthesis were identified from our transcriptome data (Table 2) and their length, identity to *Arabidopsis* and RPKM values are provided in Supplementary Data 7. In general, plant fatty acid biosynthesis occurs in plastids and is catalysed by two enzyme systems, acetyl CoA carboxylase (ACC) and fatty acid synthase (FAS). The first committed step in the FA biosynthesis is the conversion of acetyl CoA to malonyl CoA, which is catalysed by a regulatory heteromeric ACC complex composed of 4 subunits, namely biotin carboxyl carrier protein (BCCP), biotin carboxylase (BC), α-carboxyltransferase (α-CT) and β-carboxyl transferase (β-CT)^32^. In the *B*. *arvensis* transcriptome, we identified a total of 19 transcripts encoding heteromeric ACC complex subunits (13 for α-CT, 3 for BCCP, 1 for BC and 2 for β-CT). Two transcripts encoding malonyl CoA-acyl carrier protein malonyl transferase (MCMT) were identified that catalyse the transfer of the malonyl group from malonyl CoA to the acyl carrier protein (ACP). Fatty acid synthesis and elongation is further continued by a series of sequential reactions of condensation, reduction and dehydration catalysed by the FAS enzyme system, which is composed of 3-oxoacyl-(ACP) synthase (KASI, II, III), 3-oxoacyl-(ACP) reductase (KAR), 3-hydroxyacyl-(ACP) dehydratase (HAD) and enoyl-(ACP) reductase I (ER)^33^. We found a total of 19 transcripts encoding the FAS enzyme system (1 for KASIII, 4 for KASII, 4 for KASI, 5 for KAR, 1 for HAD and 4 for ER).

**Table 2:** List of enzymes involved in fatty acid biosynthesis, desaturation and TAG biosynthesis in developing seeds of *B*. *arvensis*.

Symbol	Enzymes	Arabidopsis gene ID	Number of Clustered transcripts	RPKM Value of best hit transcript
**Fatty acid biosynthesis**				
ACC1	Acetyl-CoA carboxylase/biotin carboxylase 1	AT1G36160	1	17.11
α-CT	Acetyl-CoA carboxylase carboxyl transferase subunit alpha	AT2G38040	12	34.02
BCCP	Acetyl-CoA carboxylase biotin carboxyl carrier protein	AT5G15530	3	50.92
BC	Acetyl-CoA carboxylase, biotin carboxylase subunit	AT5G35360	1	19.46
β-CT	Acetyl-CoA carboxylase carboxyl transferase subunit beta	ATCG00500	2	56.11
MCMT	Malonyl-CoA- ACP Malonyl transferase	AT2G30200	2	26.29
KASIII	3-oxoacyl-[acyl-carrier-protein] synthase III	AT1G62640	1	13.91
KASII	3-oxoacyl-[acyl-carrier-protein] synthase II	AT1G74960	4	32.6
KASI	3-oxoacyl-[acyl-carrier-protein] synthase I	AT5G46290	4	16.46
KAR	3-oxoacyl-[acyl-carrier protein] reductase	AT1G24360	5	67.56
HAD	3-hydroxyacyl-[acyl-carrier-protein] dehydratase	AT2G22230	1	26.91
ER	Enoyl-[acyl-carrier protein] reductase I	AT2G05990	4	26.44
FATA	Fatty acyl-ACP thioesterase A	AT3G25110	2	18.24
FATB	Fatty acyl-ACP thioesterase B	AT1G08510	21	15.06
LACS 8	Long-chain acyl-CoA synthetase 8	AT2G04350	2	27.40
LACS 9	Long-chain acyl-CoA synthetase 9	AT1G77590	5	3.25
LACS 4	Long-chain acyl-CoA synthetase 4	AT4G23850	11	4.03
**Fatty acid desaturation**				
SAD	Stearoyl-ACP desaturase	AT2G43710	6	9.99
FAD2	Omega-6 fatty acid desaturase (∆^12^-desaturase), Microsomal	AT3G12120	5	505.81
FAD6	Omega-6 fatty acid desaturase (∆^12^-desaturase), Choloroplastic	AT4G30950	2	10.12
FAD3	Omega-3 fatty acid desaturase (∆^15^-desaturase), Microsomal	AT2G29980	2	1763.42
FAD7/8	Omega-3 fatty acid desaturase (∆^15^-desaturase), Chloroplastic	AT3G11170	3	14.08
D6D-1	Delta-6 desaturase	—	3	11.30
D6D-2	Delta-6 desaturase	—	1	329.42
**TAG biosynthesis**				
GPAT9	Glycerol-3-Phosphate Acyltransferase 9	AT5G60620	2	13.03
LPAAT2	1-Acylglycerol-3-Phosphate Acyltransferase	AT3G57650	4	20.57
PAH2	Phosphatidate Phosphatase	AT5G42870	6	6.17
DGAT1	Acyl-CoA: Diacylglycerol Acyltransferase 1	AT2G19450	2	8.60
DGAT2	Acyl-CoA: Diacylglycerol Acyltransferase 2	AT3G51520	3	6.88
PDCT	Phosphatidylcholine:diacylglycerol cholinephosphotransferase	AT3G15820	4	20.23
CPT	Diacylglycerol Cholinephosphotransferase	AT1G13560	3	26.18
PDAT1	Phospholipid: Diacylglycerol Acyltransferase 1	AT5G13640	2	22.88
PDAT2	Phospholipid: Diacylglycerol Acyltransferase 2	AT3G44830	3	19.25
LPCAT	1-Acylglycerol-3-Phosphocholine Acyltransferase	AT1G12640	4	17.07
OLE1	Oleosin 1	AT4G25140	2	2144.55
WRI	Wrinkled 1	AT3G54320	2	35.94

Number of clustered transcripts and RPKM value of the best hit for each gene is represented.

Fatty acyl chains (C16:0, C18:0 and C18:1) synthesized in the plastid are hydrolysed to free fatty acids by fatty acyl-ACP thioesterase (FATA and FATB)^14^. FATA and FATB were identified with 2 and 21 transcripts, respectively, in our transcriptome. Long-chain acyl-CoA synthetase (LACS) catalyses the synthesis of fatty acyl-CoA from free fatty acids and CoA, which are later used in the endoplasmic reticulum for desaturation and triacylglycerol synthesis^34^. In the *B*. *arvensis* transcriptome, 2 transcripts of LACS8, 5 transcripts of LACS9 and 1 transcript of LASC4 were identified. A large number of transcripts obtained for some genes is likely due to different fragments of the same gene or different members of the same gene family. Overall, identification of key regulatory FA biosynthesis genes in our seed transcriptome helps to improve fatty acid and oil content in *B*. *arvensis* seeds.

### Identification and expression profiling of the fatty acid desaturase gene during seed development

*B*. *arvensis* seed oil contains a large amount of unsaturated fatty acids and their rate of synthesis relies on the activity of fatty acid desaturases. To understand the correlation between unsaturated fatty acid synthesis and expression of desaturase genes during seed development, we identified fatty acid desaturase genes using tBLASTN as mentioned above and analysed their expression levels using qRT-PCR. The list of fatty acid desaturation enzymes identified from our transcriptome data is presented in Table 2 and Supplementary Data 7. We identified 6 transcripts encoding plastidial stearoyl-ACP desaturase (SAD) in our transcriptome, which catalyse the first desaturation step involving the formation of monounsaturated fatty acids (oleic acid; C18:1) from saturated fatty acids (stearic acid; C18:0) in the plastid^35^. The best hit transcript showed 88% homology to *Arabidopsis* FAB2 protein sequence (AT2G43710). Oleic acid produced in the plastid undergoes further desaturation to linoleic acid (18:2) and then to α-linolenic acid(18:3) either on chloroplast membrane galactolipid by plastidial ∆^12^-desaturase (FAD6) and ∆^15^-desaturase (FAD7/8), respectively, or on endoplasmic reticulum (ER) membrane phosphatidylcholine (PC) by microsomal ∆^12^ (FAD2) and ∆^15^-desaturases (FAD3)^14^. In the *B*. *arvensis* transcriptome, we identified 7 transcripts encoding ∆^12^-desaturase (5 for FAD2 and 2 for FAD6) and the best hit transcripts showed 76% (FAD2) and 73% (FAD6) protein sequence identity to *Arabidopsis* FAD2 (AT4G12120) and FAD6 (AT4G30950) genes. *B*. *arvensis* FAD3 and FAD7/8 genes were identified with 2 transcripts each. The best hit transcript of FAD3 showed 71% protein sequence identity to *Arabidopsis* FAD3, whereas FAD7/8 showed 71% and 72% protein sequence identity to *Arabidopsis* FAD7 and FAD8, respectively.

FA biosynthesis pathway is well studied through transcriptome approach in various oil seed crops which accumulate significant amount of diverse fatty acids in their seed oils such as *Brassica napus* ^36^, *Arachis hypogea* ^37^, *Plukenetia volubilis* ^38^, *Camelina sativa* ^30^ and Arabidopsis^39^. *B*. *arvensis* seeds are unique, which accumulate SDA and GLA in addition to ALA, LA and OA, which are most common in the above oilseed crops. To understand how a similar pathway could contribute to different FA accumulation in *B*. *arvensis* seeds, we compared the transcripts or unigenes or EST (*Arabidopsis*) involved in various steps of FA biosynthesis in these oilseeds with *B*. *arvensis* transcripts and found that FA biosynthesis pathway is highly conserved except for Delta-6-desaturase gene, that produces SDA and GLA in *B*. *arvensis* (Supplementary Table S4).

**Figure 5:**
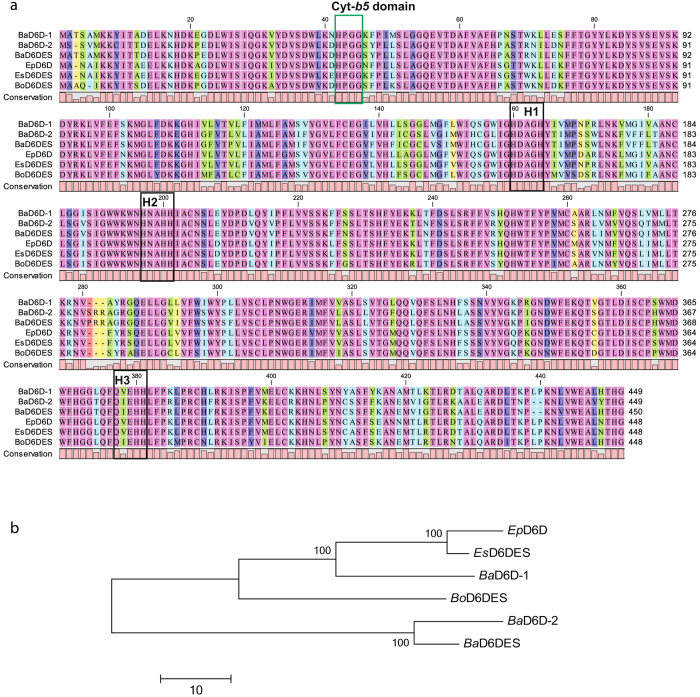
Multiple sequence alignment and phylogenetic analysis. (**a**) Amino acid sequence alignment of delta-6 desaturases (D6D) of *B*. *arvensis* isoforms (*Ba*D6D-1 and *Ba*D6D-2) identified from our transcriptome data with the reported D6D sequences of *B*. *arvensis* (*Ba*D6DES; GenBank ID: AJS13593), *Echium plantagineum* (*Ep*D6D; GenBank ID: AAZ08559), *Echium sabulicola* (*Es*D6D; GenBank ID: AAZ23035), and *Borage officinalis* (*Bo*D6DES; GenBank ID: ABU51607). Pink shades indicate identical amino acids. Green, blue, violet and yellow shades indicate dissimilar amino acids with different percentages of amino acid conservation. Dashes indicate gaps in alignment. H1, H2 and H3 boxes indicate conserved histidine boxes. The green box indicates a conserved N-terminal cytochrome *b5* heme binding motif. (**b**) Phylogenetic tree showing the relationship of *B*. *arvensis* D6D (BaD6D-1 and BaD6D-2) with orthologue proteins from the other related organisms in the Boraginaceae family. The tree was constructed by MEGA6 software using ClustalW and a neighbour-joining method. Bootstrap value = 1000.

GLA and SDA are formed by the desaturation of LA and ALA respectively at the ∆^6^ position, which is catalysed by delta-6 desaturase (D6D)^40^. Using the only reported D6D protein sequence of *B*. *arvensis* (Protein ID: AJS13593.1) as a query in tBLASTN, we identified two transcripts, c55726_g1_i2 and c42659_g2_i2, encoding a full length D6D coding sequence in our data with protein sequences identity of 95% and 77%, respectively. The results of amino acid sequence alignment and phylogenetic analysis indicate that transcripts c55726_g1_i2 and c42659_g2_i2 are isoforms (Fig. 5a and b). Transcript c42659_g2_i2, represented as D6D-1, is more closely related to other members of the Boraginaceae family (*Echium plantagineum*, *Echium sabulicola* and *Borage officinalis*) with 85–92% protein sequence identity, whereas transcript c55726_g1_i2, represented as D6D-2 (same as earlier reported D6D), diverged from other members of Boraginaceae with 73–75% protein sequence identity. Furthermore, both isoforms have 75.2% and 80.5% identity at the amino acid and nucleotide level, respectively. Gene expression levels of two D6D isoforms could not be determined separately because of high nucleotide identity. However, based on the RPKM values obtained from our developing seed transcriptome, we found that the D6D-1 isoform had a higher RPKM value (329.42) compared to that of the D6D-2 isoform (11.3). The higher amount of SDA (21%) in a *B*. *arvensis* seed compared to GLA (3.9%) suggests that D6D-1 having high RPKM value and D6D-2 having a lower RPKM are probably involved in SDA and GLA formation respectively. Further functional characterization of two D6D isoforms need to be carried out in detail to validate them.

**Figure 6:**
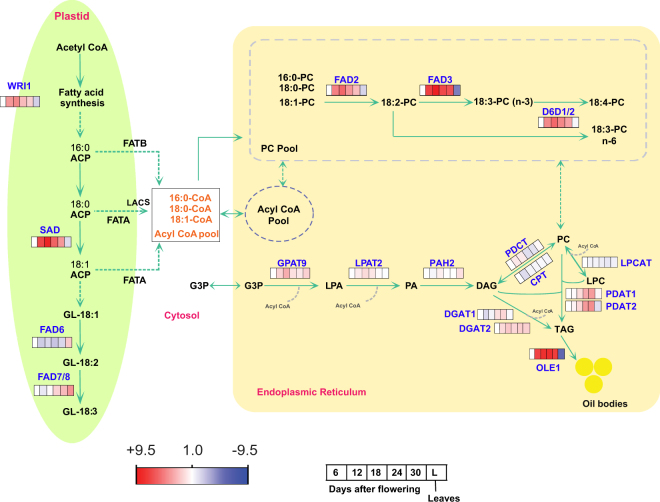
Schematic representation of a *B*. *arvensis* oil biosynthetic pathway rich in stearidonic acid. The relative expression of genes involved in fatty acid desaturation and triacylglycerol biosynthesis in developing seeds(6, 12, 18, 24, 30 DAF) and leaves of *B*. *arvensis* were analysed through qRT-PCR. Alpha-tubulin and clathrin adaptorcomplex (CAC) were used as internal reference genes. Fold change is represented as log2 in a heat map. Stage-1(6 DAF) was used as a calibrator and its expression was set as 1.0. ACP: acyl-Carrier protein; GL: galactolipid; G3P: *sn*-glycerol-3 phosphate; PA: phosphatidic acid; LPA; lysophosphatidic acid; LPC: lysophosphatidylcholine; DAG: diacylglycerol; TAG: triacylglycerol; PC: phosphatidylcholine. For full name of genes, see Table 2.

Expression profiling of SAD6, FAD2, FAD3 and D6D genes during seed development showed a bell shaped expression pattern with low expression at 6 DAF and then their expression level increased drastically reaching a maximum at18 DAF (Fig. 6), which is in agreement with high levels of ALA and SDA content in the seed observed between 12 to 30 DAF. In leaf, expression levels of SAD6, FAD2 and FAD3 was less compared to seed, suggesting active unsaturated fatty acid synthesis in seed. FAD6 and FAD7/8 showed maximum expression in leaf compared to seed indicating its major role of LA and ALA synthesis in leaf. SDA content of leaf was similar to seeds at 6 DAF which is positively correlated with their similar D6D gene expression profiles. Further, the expression level of FAD3 is very high throughout the seed development compared to other desaturases which coincide with high levels of ALA in the seed. Similar bell shaped gene expression profiles for SAD6, FAD2, FAD3 and FAD7/8 were observed in *Arabidopsis*, *Brassica* and *Perilla* seed development^21, 41, 42^. Overall, coordinated expression of all desaturase diverts the flux of saturated fatty acid more towards PUFA biosynthesis, making *B*. *arvensis* seed oil PUFA (ALA and SDA) rich.

### Identification and expression profiling of TAG biosynthesis genes during seed development

Triacylglycerol biosynthesis in ER occurs through two metabolic pathways, namely acyl CoA dependent pathway and acyl CoA independent pathways^11, 43^. All the key enzymes involved in TAG biosynthesis were identified in our transcriptome data (Table 2 and Supplementary Data 7). In the acyl CoA dependent pathway or Kennedy pathway, acyl CoAs are sequentially acylated at sn-1 and sn-2 positions of glycerol-3 phosphate by glycerol-3 phosphate acyltransferase (GPAT) and 1-acylglycerol-3-phosphate acyltransferase (LPAAT) to produce phosphatidic acid, which is later dephosphorylated to diacylglycerol (DAG) by phosphatidate phosphatase (PAP). Finally, diacylglycerol acyltransferase (DGAT) produces TAG by transferring an acyl-CoA to DAG^13, 44^. In *B*. *arvensis* transcriptome, we identified 2 transcripts encoding GPAT9, 4 transcripts for LPAAT2, 6 transcripts for PAP, 2 transcripts for DGAT1 and 3 transcripts for DGAT2. The expression profiles of TAG biosynthesis enzymes are shown in Fig. 6. Expression profiling of *B*. *arvensis* GPAT9 and LPAAT2 showed bell-shaped expression patterns during seed development with a peak at 18 DAF, whereas in leaves, GPAT9 and LPAAT2 had similar expression to seeds at 12 DAF and 6 DAF, respectively. PAP expression showed constant expression in all stages of seed development, and its expression was higher in leaves compared to that in seeds. DGAT1 is expressed in both developing seeds and leaves but its expression occurs more in the late maturation phase (24–30 DAF). Expression of DGAT2 increased at 12 DAF and then remained constant throughout seed development. Our results on DGAT1 and DGAT2 expression patterns clearly indicate the active involvement of DGAT2 (throughout seed development) and DGAT1 (at late maturation phase) in TAG biosynthesis, which agrees with the gradual increase in TAG content from 12 DAF to 30 DAF. A similar expression profile was reported for DGAT1 and DGAT2 in *Perilla* during seed development^21^.

As described, PC is the substrate for fatty acid desaturation. A flux of fatty acid into TAG from PC by either acyl-editing reactions or PC-DAG interconversion or an acyl-CoA independent pathway probably enriches the oil with more PUFA (ALA and SDA). In an acyl-editing reaction, lysophosphatidylcholine acyltransferase (LPCAT) enriches the acyl Co-A pool with PUFA through constant acylation and deacylation of fatty acids esterified to PC. Later, PUFAs enriched in the acyl-CoA pool enter the Kennedy pathway for TAG synthesis^43^. We identified four transcripts encoding LPCAT in our transcriptome data. LPCAT has constant expression throughout seed development and is also expressed similarly in leaves, which suggests there is constitutive acyl editing function (Fig. 6). Phosphatidylcholine:diacylglycerol cholinephosphotransferase (PDCT) and Diacylglycerol cholinephosphotransferase (CPT) catalyse PC-DAG inter-conversion through phosphocholine group exchange^45^. In our transcriptome, we identified four transcripts encoding PDCT and three transcripts encoding CPT. Expression profiling of PDCT showed higher expression at 12 and 18 DAF, and then the expression declined drastically when the seeds reached maturation phase, whereas leaves showed an expression pattern similar to that in seeds at 6 DAF. A similar expression pattern was observed for PDCT in *Perilla* seed development^21^. CPT showed a constant expression pattern throughout the seed development except for a slight decline at 12 DAF, whereas the expression pattern in leaves was similar to that in developing seeds (Fig. 6). In the acyl-CoA independent pathway, TAG is synthesized by transfer of sn-2 fatty acid from PC to the sn-3 position of DAG by Phospholipid: Diacylglycerol Acyltransferase (PDAT)^15^. We found two transcripts encoding PDAT1 and three transcripts encoding PDAT2 in our transcriptome data. Both PDAT1 and PDAT2 showed high expression at the mid and late stages (18–30 DAF) of seed development, which indicates the major role of these transcripts in enriching TAG with PUFA during 18–30 DAF. PDAT2 showed seed-specific expression, whereas PDAT1 was expressed both in seeds and leaves (Fig. 6). Similar seed specific expression of PDAT2 was observed in the *Perilla* and *Physaria* seed transcriptomes^21, 46^. Overall, identification of full length ORFs of LPCAT, PDCT, CPT and PDAT in our transcriptome data provides a molecular basis for further investigating their roles in enriching the TAG with PUFA.

After synthesis, TAG molecules are usually stored as oil bodies, which are surrounded by a layer of phospholipids embedded with oleosins^47^. We found 2 transcripts encoding OLE1 in our data with seed-specific expression. OLE1 showed a very high expression pattern from 12 DAF to 30 DAF, which indicated its major role in oil body accumulation (Fig. 6). WRINKLED (WRI1), which is an AP2/EREBP family transcription factor, plays a major role in regulating oil accumulation in seed tissues by controlling the expression of FA biosynthesis genes^48^. Earlier studies of WRI1 over expression showed an increase in the oil content by 10–40% in *Brassica* and Maize^49, 50^. The *B*. *arvensis* orthologue of Arabidopsis WRI1 was identified in our data and it had seed-specific expression with a bell-shaped expression pattern during seed development, which peaked at 18 DAF (Fig. 6). The finding of *B*. *arvensis* WRI1 would suggest that it can be a potential target for increasing fatty acid synthesis in plastids and for increasing oil content in the seed.

## Conclusion

*B*. *arvensis* is one of the richest natural sources of SDA in the plant kingdom and is nutritionally valuable due to its high omega-3 fatty acid content in the seed. Our results for seed oil and PUFA accumulation showed accumulation patterns specific to each development stage. The transcriptome sequencing and *de novo* assembly for developing seeds was carried out to maximize the gene space associated with lipid biosynthesis. We identified most of the transcripts encoding enzymes involved in fatty acid biosynthesis, desaturation and TAG assembly. Identification and characterization of temporal expression profiles of key genes (delta-6 desaturase, DGAT, LPCAT, PDAT and PDCT) involved in PUFA (SDA) and oil biosynthesis not only provide a molecular basis for future research into understanding the mechanisms of high SDA containing TAG biosynthesis in *B*. *arvensis* seeds but also for potential targets in genetic reprogramming of other oilseed crops to produce SDA enriched oils. The discovery of a large number of SSR-containing sequences in our transcriptome data will be useful in a marker-assisted plant breeding programme. Finally, it is anticipated that the first public transcriptome data established in the current study may assist in developing research programmes focusing on improving the nutritional quality and quantity of SDA-rich *B*. *arvensis* seed oil through genomics-assisted plant breeding programmes.

## Methods

### Plant material and growth conditions

*B*. *arvensis* seeds from a naturally growing wild accession were collected from the north temperate region of India (Pampore, Jammu and Kashmir; latitude 34.02°N and longitude 74.93°E) and germinated through cold stratification (4–6 °C) for 7–10 days. Germinated seeds were sown in nursery trays and then transplanted to red loamy soil in a poly house (18 °C–28 °C) located at the CSIR-Central Food Technological Research Institute research farm, Mysuru, India (latitude 12°19′39.3456″N; longitude 76°37′16.1364 E; average altitude 770 MSL; average rainfall 804.2 mm). The plants were assessed for adaptability and yield potential for four successive generations (120–130 days per generation). The highest yielding line (BA-26) was selected as experimental material for the present study (Supplementary Fig. S4). Fully opened flowers were tagged and tagging date was recorded as 0^th^ DAF. Developing seeds at 6, 12, 18, 24, 30 DAF and leaf samples from mature plants were collected, immediately frozen in liquid nitrogen and stored at −80 °C until further use.

### Determination of total lipid content and fatty acid composition

Total lipids were extracted according to the methods of Hara and Radin^51^. Briefly, freeze-dried seeds from five different developing stages (6, 12, 18, 24, 30 DAF) and mature leaves were homogenized separately, using a mortar and pestle. The lipids were extracted three times with hexane: isopropanol (3:2 v/v). The extracts were combined and washed with 1 M KCl to remove proteins and carbohydrates. The final solvent was dried under a nitrogen stream. Extracted lipids were weighed and expressed as a percentage of dry weight. Neutral lipid profiling was carried out on a silica-TLC plate using the solvent system petroleum ether: diethyl ether: acetic acid (70:30:1, v/v), followed by manganese chloride charring.

Total fatty acid composition was determined by GC-MS. Briefly, a known amount of internal standard (Heptadecanoic acid; C17:0) was added to the extracted lipids, dried and esterified with 1 ml of BF_3_-methanol complex solution (13–15% BF_3_ basis, Sigma, USA) at 65 °C for 1 hr to obtain fatty acid methyl esters. After cooling, hexane: water (1:1 v/v) was added to the tubes, vortexed and centrifuged at 1000 g for 5 min. The upper organic layer was transferred to a GC vial, dried under a nitrogen stream and re-suspended in HPLC grade hexane for analysis by GC-MS (Perkin Elmer, USA equipped with Turbo mass Gold mass spectrometer) using a TR-FAME column (70% cyanopropyl polysilphenylene-siloxane; 30 m length, 0.25 mm ID, 0.25 m film thickness; Thermo Scientific, USA). GC and MS conditions were represented in Supplementary Fig. S1. Fatty acids were identified by comparison of their mass spectrum to known standards (Sigma, USA), and the results were confirmed by a mass spectral library search (NIST version 2.0). All experiments were done in biological triplicates and the results were expressed as the mean of three independent measurements.

### Total RNA extraction, library construction and transcriptome sequencing

Total RNA was isolated in duplicate separately from five different stages of developing seeds (6, 12, 18, 24 and 30 DAF) using a Qiagen RNeasy plant mini kit. The purity, integrity and yield of RNA were analysed with an Agilent 2100 Bioanalyzer (Agilent Technologies, USA). Samples with an RNA integrity number (RIN) value greater than 8 were used for sequencing library preparation with the Illumina compatible NEBNext^®^ Ultra™ Directional RNA Library Prep Kit (New England Biolabs, MA, USA). One hundred ng of total RNA from each stage were pooled and subjected to poly(A) purification of mRNA. Purified mRNA was fragmented using divalent cations at elevated temperatures. First strand cDNA was synthesised using random hexamer primers and ProtoScript II Reverse Transcriptase in the presence of actinomycinD (Gibco, Life Technologies, CA, USA). This step was followed by second strand cDNA synthesis using NEB second strand synthesis reaction buffer and an enzyme mix. The double stranded cDNA was purified using HighPrep PCR magnetic beads (Magbio Genomics Inc, USA) and was subjected to end repair process, adenylation and then ligated to Illumina multiplex barcode adapters. The adapter ligated cDNA was purified using HighPrep beads and was subjected to 12 cycles of indexing-PCR to enrich the adapter-ligated fragments, which were further purified with HighPrep beads. An Illumina-compatible sequencing library was initially quantified by a Qubit fluorimeter (Thermo Fisher Scientific, MA, USA) and then by quantitative PCR using the Kapa Library Quantification Kit (Kapa Biosystems, Wilmington, MA, USA).The constructed cDNA library was sequenced on an Illumina NextSeq 500 platform. The RNA-Seq data was generated in FastQ format.

### *De novo* transcriptome assembly, functional annotation and pathway analysis

Raw reads of 150 paired ends (PE) generated by sequencing were subjected to a quality check by FastQC (Version 0.11.5)^52^ and further processed with the Perl script ABLT.pl (Proprietary tool of Genotypic Technology Pvt Ltd., Bangalore, India) for adapter trimming and low quality base removal towards the 3′-end. Reads less than 50 bp were discarded during processing. Processed high quality reads were *de novo* assembled using Trinity software^53^ with a hash length of 25. Assembled transcripts were then clustered using the CD-HIT program with a 90% identity cut-off ^54^. High quality clean reads were mapped to clustered transcripts using BOWTIE2 (2.0.5)^55^ software at default parameters. Following mapping, raw read count for each transcript was derived and normalized to RPKM (Reads Per Kilobase of transcript per Million mapped reads)^56^. Assembled transcripts with a sequence length above 300 bp were used for downstream transcript annotation.

Clustered transcripts were annotated by performing BLASTX^57^ against uniprot-Viridiplantae database. Gene ontology (GO) terms were assigned to transcripts based on the best hit with the above database using the in-house GO annotation pipeline (Proprietary tool of Genotypic Technology Pvt Ltd., Bangalore, India) that works based on Uniport GO ID. Clustered transcripts were also annotated by using the Eukaryotic Orthologous Group (KOG) database. The pathway analysis was done with KAAS (KEGG Automatic Annotation Server) using the bi-directional best hit method against 15 plant reference organisms (see supplementary file 3)^58^. Local BLAST (tBLASTN) was performed using BlastStation2 software (http://www.blaststation.com/intl/en/blaststation2.php). Protein sequences of Arabidopsis lipid genes were retrieved from TAIR (www.arabidopsis.org) and were used to query *B*. *arvensis* assembled transcripts using tBLASTN. The best hit for each query sequence was retained based on *E*-value, bit score and identity. MEGA6 was used for phylogenetic tree construction using ClustalW and neighbour-joining analysis. Amino acid sequence alignment of the proteins was performed using CLC Main Workbench 7 (CLC bio, Aarhus, Denmark) software.

### Transcription factors and SSR marker identification

Transcription factors were identified by a homology search of clustered transcripts against plant transcription factor database v4.0^59^. Simple sequence repeat (SSR) regions were predicted from clustered transcripts using the MIcroSAtellite (MISA) tool. Transcripts encoding TFs and SSR were mapped to KEGG pathways.

### Validation of transcriptome assembly

To validate the precision and quality of transcriptome assembly, PCR amplification of 25 randomly selected genes with gene-specific primers was carried out using pooled cDNA (from five stages of seed development) as a template. The gene-specific primer for all the genes was designed based on the transcriptome sequence using Primer3Plus software^60^. The primer sequences are provided in Supplementary Table S5. PCR was carried out using *Taq* DNA polymerase with standard *Taq* buffer (New England Biolabs, MA, USA). The PCR conditions that are used are as follows: Initial denaturation of 95 °C for 1 min followed by 35 cycles of denaturation at 95 °C for 20 sec, annealing temperature for 30 sec, extension of 68 °C for 2 min and final extension at 68 °C for 5 min. PCR products were analysed on a 0.8% agarose gel. Further, PCR amplified products of all the genes were sequenced using Sangers platform and compared with the *De novo* assembled transcripts to confirm their sequence identities.

### Quantitative Real-Time PCR analysis

Total RNA was isolated separately from the developing seeds (6, 12, 18, 24, 30 DAF) and leaves using Trizol reagent. RNA concentration and purity ratios (A260/280 and 260/230) were quantified using a NanoDrop 2000 spectrophotometer (Thermo Scientific). Then, 1 µg of total RNA was used as sample for first strand cDNA synthesis using the Verso cDNA synthesis kit (Thermo Scientific) with an oligo dT primer. The qRT-PCR reactions were performed on a CFX96 Real- Time PCR system (Bio-Rad) in a total volume of 10 µl containing 5 µl of 2x iTaq Universal SYBR Green Supermix (Bio-Rad), 0.5 µl of each primer (500 nM), 3 µl of 1:10 diluted cDNA template from samples and 1 µl sterile distilled water. The thermal cycling programme was performed at 95 °C for 30 s followed by 40 cycles at 95 °C for 5 s, 54 °C for 30 s and 60 °C for 30 s. Gene-specific primers were designed using Primer3Plus software with the following parameters: melting temperature set to 58–60 °C and product length set to 100–120 bp^60^. The specificity of the primer pairs was confirmed by performing PCR using a mixture of cDNA from different samples and analysed on a 2% agarose gel (Supplementary Fig. S5a and b). qBase plus software v3.1^61^ was used to determine primer efficiencies for each primer pair by generating the standard curves with a series of five, 10-fold dilutions of the cDNA mixture (10^6^–10^2^). Normalized relative fold expression was calculated using qBase plus software after normalization with two reference genes: α-Tubulin and clathrin adaptor complex (CAC), which are stably expressed in seeds^62^. Seed samples at 6 DAF were used as a calibrator for the remaining samples. The fold change was log_2_ transformed and represented as a heat map. All reactions were performed with three independent biological replicates, including no template controls. The list of genes, primers and their efficiency values is provided in Supplementary Table S6.

### Data Availability

Raw sequence reads are available at the NCBI Sequence Reads Archives (SRA) under accession number SRS1714215. BioProject accession number PRJNA344425. BioSample accession number SAMN05823147.

## Electronic supplementary material

Supplementary Information

Supplementary Data 1

Supplementary Data 2

Supplementary Data 3

Supplementary Data 4

Supplementary Data 5

Supplementary Data 6

Supplementary Data 7
