# Supplementary material for: Unravelling a stearidonic acid-rich triacylglycerol biosynthetic pathway in the developing seeds of Buglossoides arvensis: A transcriptomic landscape
Source: Sci Rep. 2017 Sep 5;7:10473. doi: 10.1038/s41598-017-09882-y (PMC5585386; doi:10.1038/s41598-017-09882-y)

Unravelling a stearidonic acid rich triacylglycerol biosynthetic pathway in the developing seeds of *Buglossoides arvensis*: A transcriptomic landscape

Sreedhar R.V.[1,2#], P. Prasad[1,2#], L. Prasanna Anjaneya Reddy[1], Ram Rajasekharan[1], Malathi Srinivasan[1*]

[1]Department of Lipid Science, CSIR-Central Food Technological Research Institute (CSIR-CFTRI), Mysuru-570 020, India.
[2]Academy of Scientific and Innovative Research, CSIR-CFTRI, Mysuru-570020, India.

[#]Both authors have contributed equally

*Corresponding author
Malathi Srinivasan
CSIR-Central Food Technological Research Institute
Mysuru-570 020
India
Tel: +91-821-2514153
E-mail: m.srinivasan@cftri.res.in

Supplementary Table S1: Annotation statistics of *De novo* assembled transcripts

Annotation Database	Annotated Number	300>=length<1000	Length>=1000
Total Transcripts	102888		
Uniprot-Viridiplantae	63823	33926	29897
KOG	57146	28829	28317
KEGG	11439	7589	3850

Supplementary Table S2: List of transcripts encoding major Transcription factors (TF) involved in oil biosynthesis. TFs were identified by tBLASTN using *Arabidopsis* TFs amino acid sequences as query.

S.No	Transcription factor	Best hit transcript ID	*Arabidopsis* Gene ID	Homology (%) to *Arabidopsis* homologue
**1.**	LEAFY COTYLEDON 1 (LEC1)	c43466_g1_i2	AT1G21970	73
**2.**	FUSCA 3 (FUS3)	c53059_g1_i2	AT3G26790	57
**3.**	WRINKLED 1 (WRI1)	c51431_g2_i2	AT3G54320	80
**4.**	HIGH LEVEL EXPRESSION OF SUCROSE INDUCIBLE GENE 2 (HSI2)	c51910_g3_i1	AT2G30470	47
**5.**	HIGH LEVEL EXPRESSION OF SUCROSE INDUCIBLE GENE 1 (HSI1)	c51910_g3_i1	AT4G32010	44
**6.**	ARABIDOPSIS SIX-B-INTERACTING PROTEIN 1-LIKE 1 (ASI4)	c49273_g1_i2	AT1G54060	53
**7.**	PICKLE (PKL)	c55592_g1_i2	AT2G25170	65
**8.**	GLABRA 2 (GL2)	c53258_g1_i2	AT1G79840	60
**9.**	APETALA 2 (AP2)	c51866_g1_i5	AT4G36920	55

Supplementary Table S3. Statistics of simple sequence repeats (SSRs) identified from *B. arvensis* transcriptome.

Total number of sequences examined	102888
Total size of examined sequences (bp)	94941727
Total number of identified SSRs	11271
Number of SSR containing sequences	10021
Number of sequences containing more than 1 SSR	1096
Number of SSRs present in compound formation	521
Monomer [p1]	4658
Dimer [p2]	2498
Trimer [p3]	3987
Tetramer [p4]	101
Pentamer [p5]	14
Hexamer [p6]	13

Supplementary Table S4: Comparative table representing number of transcripts or unigenes or EST (Arabidopsis) for each gene involved in various steps of FA biosynthesis.

<table>
<tr><th rowspan="2">Symbol</th><th rowspan="2">Arabidopsis gene ID</th><th colspan="6">Number of transcripts/unigenes</th></tr>
<tr><th>B. arvensis</th><th>Brassica napus[37]</th><th>Plukenetia volubilis[39]</th><th>Arachis hypogea[38]</th><th>Camelina sativa[30]</th><th>Arabidopsis EST counts[40]</th></tr>
<tr><td colspan="8">Fatty acid biosynthesis</td></tr>
<tr><td>ACC1</td><td>AT1G36160</td><td>1</td><td>-</td><td>2</td><td>-</td><td>3</td><td>4</td></tr>
<tr><td>α-CT</td><td>AT2G38040</td><td>12</td><td>3</td><td>5</td><td>2</td><td>18</td><td>17</td></tr>
<tr><td>BCCP</td><td>AT5G15530</td><td>3</td><td>4</td><td>6</td><td>9</td><td>10</td><td>2</td></tr>
<tr><td>BC</td><td>AT5G35360</td><td>1</td><td>3</td><td>2</td><td>12</td><td>2</td><td>22</td></tr>
<tr><td>β-CT</td><td>ATCG00500</td><td>2</td><td>3</td><td>6</td><td>-</td><td>2</td><td>0</td></tr>
<tr><td>MCMT</td><td>AT2G30200</td><td>2</td><td>2</td><td>1</td><td>6</td><td>3</td><td>2</td></tr>
<tr><td>KASIII</td><td>AT1G62640</td><td>1</td><td>3</td><td>2</td><td>4</td><td>1</td><td>1</td></tr>
<tr><td>KASII</td><td>AT1G74960</td><td>4</td><td>6</td><td>1</td><td>10</td><td>3</td><td>10</td></tr>
<tr><td>KASI</td><td>AT5G46290</td><td>4</td><td>1</td><td>10</td><td>-</td><td>6</td><td>14</td></tr>
<tr><td>KAR</td><td>AT1G24360</td><td>5</td><td>2</td><td>2</td><td>20</td><td>8</td><td>5</td></tr>
<tr><td>HAD</td><td>AT2G22230</td><td>1</td><td>5</td><td>1</td><td>3</td><td>5</td><td>2</td></tr>
<tr><td>ER</td><td>AT2G05990</td><td>4</td><td>1</td><td>3</td><td>3</td><td>2</td><td>10</td></tr>
<tr><td>FATA</td><td>AT3G25110</td><td>2</td><td>2</td><td>2</td><td>4</td><td>7</td><td>1</td></tr>
<tr><td>FATB</td><td>AT1G08510</td><td>21</td><td>4</td><td>3</td><td>5</td><td>8</td><td>6</td></tr>
<tr><td>LACS 8</td><td>AT2G04350</td><td>2</td><td rowspan="3">10</td><td rowspan="3">41</td><td rowspan="3">33</td><td>-</td><td rowspan="3">3
1
10</td></tr>
<tr><td>LACS 9</td><td>AT1G77590</td><td>5</td><td>9</td></tr>
<tr><td>LACS 4</td><td>AT4G23850</td><td>11</td><td>-</td></tr>
<tr><td colspan="8">Fatty acid desaturation</td></tr>
<tr><td>SAD</td><td>AT2G43710</td><td>6</td><td>3</td><td>3</td><td>8</td><td>3</td><td>20</td></tr>
<tr><td>FAD2</td><td>AT3G12120</td><td>5</td><td>1</td><td>6</td><td>13</td><td>3</td><td>260</td></tr>
<tr><td>FAD6</td><td>AT4G30950</td><td>2</td><td>2</td><td>2</td><td>-</td><td>-</td><td>36</td></tr>
<tr><td>FAD3</td><td>AT2G29980</td><td>2</td><td>1</td><td>2</td><td>-</td><td>1</td><td>6</td></tr>
<tr><td>FAD7/8</td><td>AT3G11170</td><td>3</td><td>8</td><td>8</td><td>-</td><td>8</td><td>26</td></tr>
<tr><td>D6D-1</td><td>-</td><td>3</td><td>-</td><td>-</td><td>-</td><td>-</td><td>-</td></tr>
<tr><td>D6D-2</td><td>-</td><td>1</td><td>-</td><td>-</td><td>-</td><td>-</td><td>-</td></tr>
</table>

Supplementary Table S5: List for primers used for assembly validation (PCR amplification and Sangers sequencing).

S. No	Gene name (Transcript ID)	Primers (5'---------3')	Annealing temperature	Size of the amplicon
1.	Formate Dehydrogenase (FDH) (c49054_g1_i1)	FP: CCACTCTTTCATGGCGATG<br>RP: TCAACGGTATTGACTTGCTAG	56°C	1141bp
2.	Uricase (URI) (c46123_g1_i1)	FP : CGTGAGATATGGCGGAGA<br>RP: TCACATTTTGGACTGAACGC	56°C	938bp
3.	Class I glutamine amidotransferase-like superfamily protein (GAT) (c44846_g1_i1)	FP: CCTACCATAGTCTAGAAGTGTCA<br>RP: TTAAAATGAGACGCGCACA	56°C	1208bp
4	Adenosine kinase 2 (ADK2) (c48968_g1_i2)	FP: CTTGAGACGAGTGATTCAACA<br>RP: CGGTAATATGGCGTTCAAAC	56°C	1223bp
5	Monodehydroascorbate reductase 1 (MDAR1) (c49725_g2_i1)	FP: TGTCCGATCAATATTATGGCTG<br>RP: GGTTACAGGTTACAGCATTCA<br>MRP: TCACCGACATTGTCTCCATA	54°C	1338bp
6	NAD(P)-binding Rossmann-fold super family protein (FLDH) (c47782_g1_i1)	FP: GAGTTTCCCAAGATGCAAGA<br>RP: TTGTGCTACATACCTCCTGT	56°C	1085bp
7	4-coumarate:CoA ligase 3 (4CL3) (c52280_g1_i1)	FP: AGGTGTGGGAACAACAAATC<br>RP: GTAAGAATTCAAGAGGAGGCC<br>MFP: AACCAACTTCCATCAACTCC<br>MRPCCACCCTAATCGAACTCAGA	56°C	1846bp
8	Pantoate-beta-alanine ligase (PANC) (c50667_g1_i3)	FP: GCACAAGAATATGGCAGCTA<br>RP: GGCTGAATCTGAGTTTTCGA	56°C	1040bp
9	Proliferating cell nuclear antigen 2 (PCNA2) (c45183_g1_i1)	FP: CCCTCTTCGAAAATGTTGGA<br>RP: CACATATGAAACGGATCTTCAC	56°C	894bp
10	Aldolase superfamily protein (ALD) (c41346_g1_i1)	FP: GATCCCACACTCTACTGTCA<br>RP: TACACATTTGGAGGAGGCC	56°C	1224bp
11	Adenylate kinase 1 (ADK 1) (c46003_g1_i2)	FP: CACTCATCTAATCTCTCAACCA<br>RP: CAATGTTTGCCCGTGACTAT	54°C	837bp
12	cytosolic NADP+ dependent isocitrate dehydrogenase (cICDH) (c52536_g1_i1)	FP: TTCGACAAGATCAAGGTTGC<br>RP: GGATCTCAGATCAGCTGCTA	56°C	1218bp
13	ascorbate peroxidase 3 (APX3) (c42820_g2_i1)	FP: GTCCATTAACATTGACCGAAAG<br>RP: TCTTAAGCTTCTTGCCACAC	56°C	1070
14	Ras-related small GTP-binding family protein (RAS) (c43771_g2_i6)	FP: CAATTGGTTGTTGAATGGCG<br>RP: TAGAACCTACAAACGCCCTT	56°C	706
15	squalene synthase 1 (SQS1) (c51006_g1_i2)	FP:ATGGGAGGGGGTAATATTATAGG<br>RP:CAACTTTCCACATGAAACGG	57°C	1348bp

16	glyceraldehyde-3-phosphate dehydrogenase C subunit 1(GAPC1) (c49443_g4_i2)	FP: TCTAACACCTACTACTCTCATGG<br>RP: GATACTGTCCAAATATGCATCG<br>MRP:ACACTCTAACTGAAGCCACA	57°C	1329bp
17	Rubisco activase (RCA) (c45330_g1_i1)	FP: AGCCAAAATCGACACAATCA<br>RP: ATACCATTGGACTCCCTTACA<br>MRP: CTCCTGTTCTTGAACAAGCA	57°C	1453bp
18	Actin-11 (ACT11) (c53638_g2_i3)	FP: ATGGCAGACACTGAGGATAT<br>RP: CCATGTCAACCAACCTTTGT	56°C	1235bp
19	Alpha tubulin (AT) (c49714_g3_i3)	FP: ATGAGGGAGTGCATTTCGAT<br>RP: ACTCCCCTTAGTAGTCTTCAC	56°C	1360bp
20	Diacylglycerol acyltransferase 2 (DGAT2) (c50394_g1_i2)	FP: TAAGATGGAGGTGAACGGC<br>RP: ACAGTTCTACCATGCCAAAG	56°C	1111bp
21	fatty acid desaturase 2 (FAD2) (c51804_g2_i1)	FP: TGAACAATGGGTGCTGGA<br>RP: GTCAGTTTCAAGTTCTCTGATG	56°C	1291bp
22	hydroxysteroid dehydrogenase 1 (HSD1) (c44755_g2_i1)	FP: GCCATGGGACTTGTTGATTT<br>RP: TACACAACTCGACTGCAATC	56°C	1164bp
23	Caleosin (CAL) (c45867_g4_i2)	FP: GGTAGGAGAAGCACCTCTAG<br>RP: CACTGGTTCCTTGGCATTTA	56°C	886bp
24	Delta-6 desaturase (D6D) (c42659_g2_i2)	FP: ATGGCTACTTCTGCAATGAAGA<br>RP: TTAACCATGAGTGTGAAGAGCT	58°C	1350 bp
25	Oleosin (OLE) (c41275_g1_i1)	FP: ATGGCTATGACTGTTGATCACCA<br>RP: TTACTGGTTCCCTCCCTGAAC	56°C	441 bp

Supplementary Table S6: List for primers used for qRT-PCR.

S.No	Gene name	Primers (5'---------3')	Product Size	Amplification Efficiency (%)	Amplification factor	R^2 Value
1	GPAT9	Forward primer: AAGGGTGCCTTTGAACTTGG<br>Reverse primer: AAGCTGCAACAGATGTGTGG	120	104	2.04	0.996
2	LPAT	Forward primer: TGGCCGACCTTTGAAATCTC<br>Reverse primer: TTTCCACGAAGACAGAAGCG	106	94	1.94	0.998
3	PAP	Forward primer: TGGGAAACAGCAGGTTGATG<br>Reverse primer: ACCCAAGGCATGAATTGTCC	120	96	1.96	0.998
4	CPT	Forward primer: ATGCATGGCTGATTGAAGGG<br>Reverse primer: TCCTCTGGTAACGGTAGCTG	112	96	1.96	0.998
5	PDCT	Forward primer: GCTGTTGATGGCAAACAAGC<br>Reverse primer: TCCCAAATGCCATTGCTTCG	106	93	1.93	0.996
6	PDAT1	Forward primer: AATGCCTGCTTCGTTTCCTC<br>Reverse primer: AAACAACACCGGATGCTTCG	115	103	2.03	0.998
7	PDAT2	Forward primer: ATCGGTCCAGCATTTCTTGG<br>Reverse primer: AAAATACCAGGCGCCATAGC	101	95	1.95	0.999
8	DGAT1	Forward primer: TTTCCTTGCTTGCGGAATGG<br>Reverse primer: GAAAATGTGGCATGGTACGG	108	94	1.94	0.998
9	DGAT2	Forward primer: TTTATATGGAGCGCGGTTCC<br>Reverse primer: TGGCCAAAGCAGAAAACTGG	109	94	1.94	0.999
10	LPCAT1	Forward primer: TGGTTTCCAGGTGTTAAGCC<br>Reverse primer: GGCTTTGCCGGTTTGATAAC	120	90	1.90	0.999
11	OLE1	Forward primer: TGACTTTGGCAGCAACTGTG<br>Reverse primer: AAGCAAGAAGACGGTGAGTG	101	94	1.94	0.999
12	FAB2	Forward primer: ACGCTTGATGGTGTTCGAGA<br>Reverse primer: ATTTAGGAGGTCACCGTGCC	105	101	2.01	0.988
13	FAD2	Forward primer: AATCAAAGTCCGACGTCGTC<br>Reverse primer: AGATCGCTGGAAACAATGGG	101	94	1.94	0.997
14	FAD6	Forward primer: AGGACATGATTGTGCCCACA<br>Reverse primer: TTGAATCGCCATGGCTCGTA	111	98	1.98	1
15	FAD3	Forward primer: TGGAAGCTTTTCGGATAGCG<br>Reverse primer: ATGGTTGGCATGGTGAGTTC	118	96	1.96	0.999
16	FAD7/8	Forward primer: TGTTTTCGGTTTGGCTGCTG<br>Reverse primer: TGACCACAATCATGGCCAAG	120	104	2.04	0.991
17	D6D	Forward primer: TTCAGAGTGGTTGGATTGGG<br>Reverse primer: CCAAGACAATTCGCAGCAAC	100	104	2.04	0.995

18	WRI1	Forward primer: CCACCATAATGGACGTTGGG<br>Reverse primer: ATGGCTGCCTTGTCATATGC	117	89	1.89	0.999
19	α- TUB	Forward primer: CGTGGTGATGTGGTGCCTAA<br>Reverse primer: ATTCCACACTTGAAGCCGGT	107	100	2.00	0.999
20	CAC	Forward primer: AGTTTCCAGGTTACGTCAGG<br>Reverse primer: TTCCACCTCAGCACTCAATG	114	97	1.97	0.999

Supplementary Fig S1: Mass spectra of identified fatty acids in the lipid extracts of *B. arvensis* developing seeds. GC conditions were conducted as follows: The carrier gas was helium with a1 ml/min flow rate, the injection volume was 1 µl, the split ratio was 1:20, and the temperature programme was an initial temperature of 80°C, ramp to 120°C at 5°C/min and then to 160°C/min at 8°C/min with a 3 min hold. Finally, the temperature was ramped to 200°C at 6°C/min with a 0.33 min hold. The MS detection conditions included temperatures of ion sources and quadrupole set at 180°C and 120°C. The mass spectra were recorded under electron impact ionization at electron energy of 70eV with a mass range m/z 40-400 da. (a) GC-MS chromatogram of *B. arvensis* seed oil (Stage-4). (b) Mass spectra of Palmitic acid (Hexadecanoic acid; C16:0). (c) Mass spectra of stearic acid (Octadecanoic acid; C18:0). (d) Mass spectra of Oleic acid (9-octadecenoic acid; C18:1). (e) Mass spectra of Linoleic acid (9, 12-octadecadienoic acid; C18:2). (f) Mass spectra of Gamma Linolenic acid (6, 9, 12-octadecatrienoic acid; C18:3n-6). (g) Mass spectra of α- linolenic acid (9, 12, 15-octadecatrienoic acid; C18:3n-3).(h) Mass spectra of Stearidonic acid (6, 9, 12, 15-octadecatetraenoic acid; C18:4)

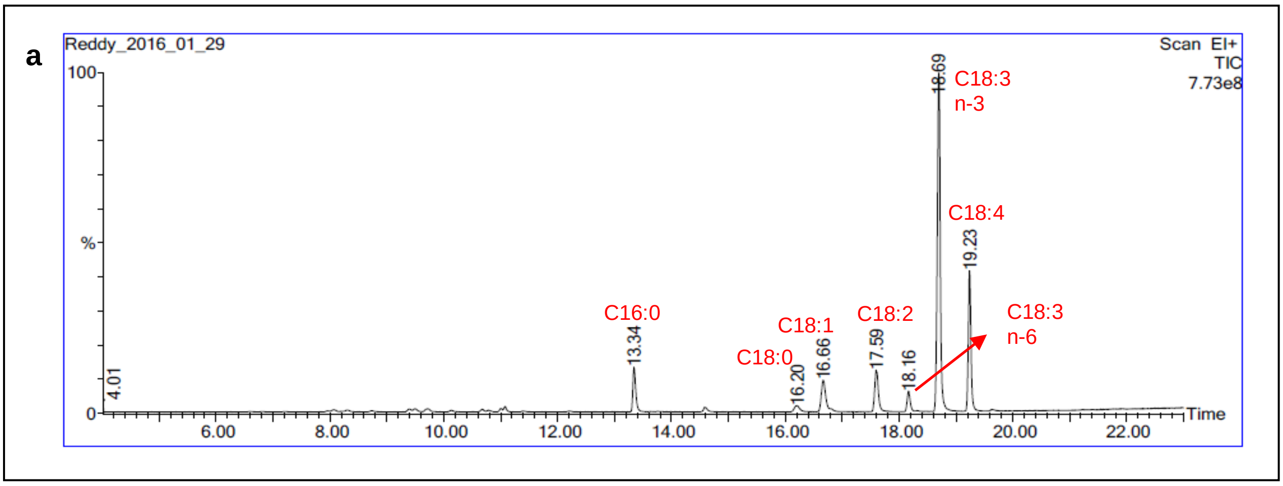

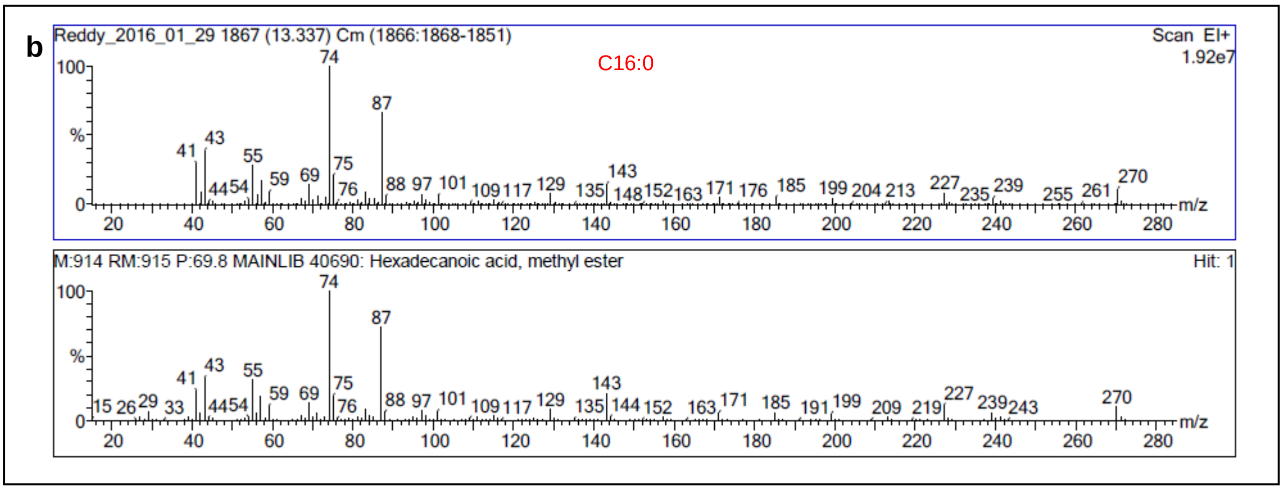

c

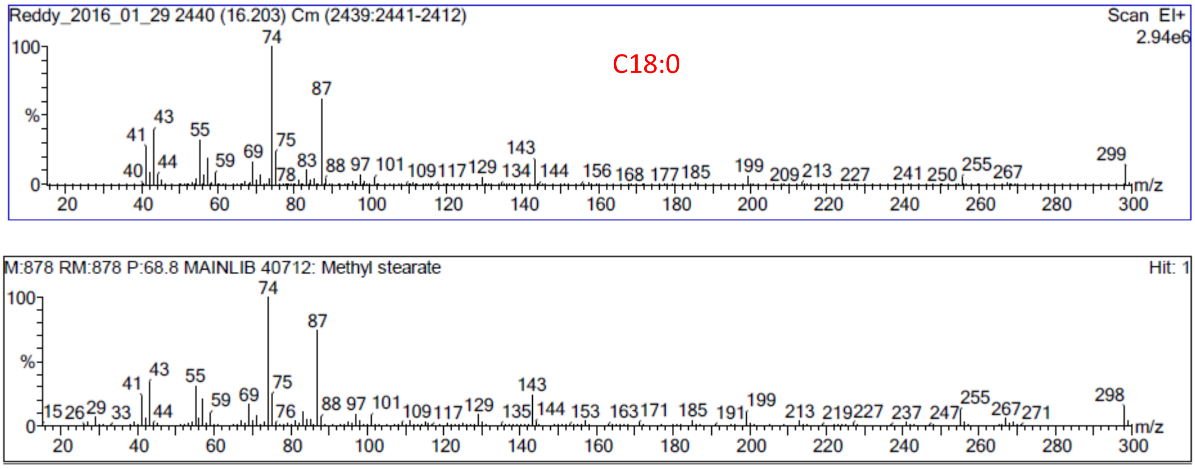
Reddy_2016_01_29 2440 (16.203) Cm (2439:2441-2412)
Scan EI+
2.94e6
C18:0
M:878 RM:878 P:68.8 MAINLIB 40712: Methyl stearate
Hit: 1
m/z

d

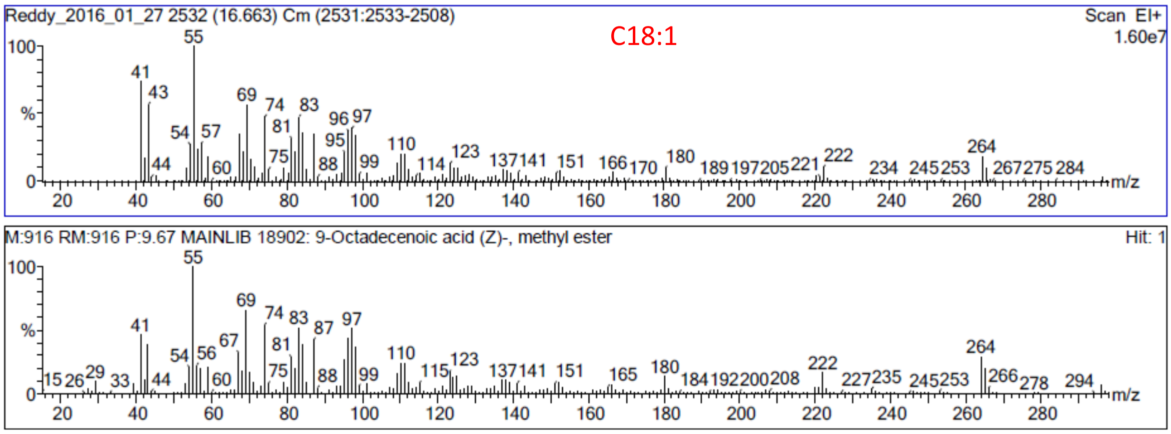
Reddy_2016_01_27 2532 (16.663) Cm (2531:2533-2508)
Scan EI+
1.60e7
C18:1
M:916 RM:916 P:9.67 MAINLIB 18902: 9-Octadecenoic acid (Z)-, methyl ester
Hit: 1
m/z

e

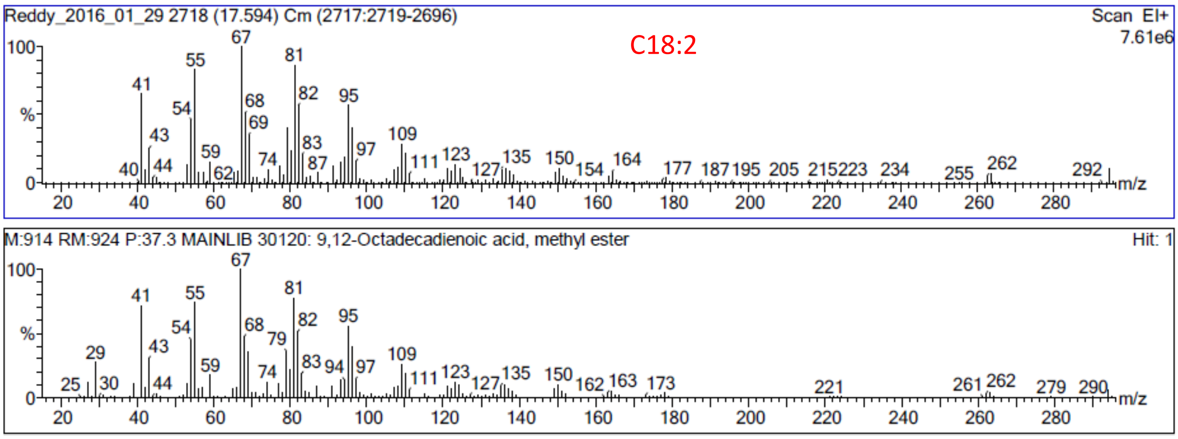
Reddy_2016_01_29 2718 (17.594) Cm (2717:2719-2696)
Scan EI+
7.61e6
C18:2
M:914 RM:924 P:37.3 MAINLIB 30120: 9,12-Octadecadienoic acid, methyl ester
Hit: 1
m/z

f

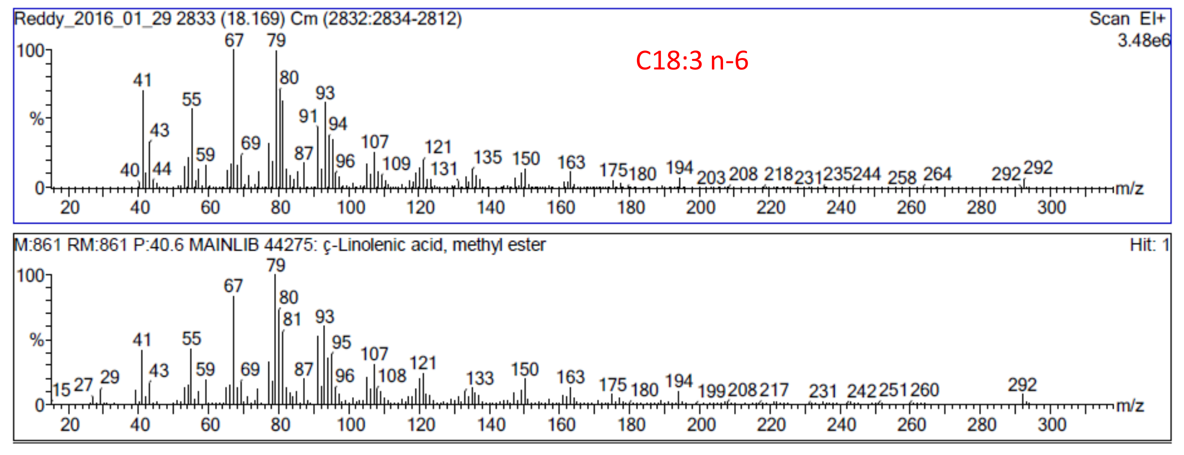
Reddy_2016_01_29 2833 (18.169) Cm (2832:2834-2812)
Scan EI+
3.48e6
C18:3 n-6
M:861 RM:861 P:40.6 MAINLIB 44275: ç-Linolenic acid, methyl ester
Hit: 1
m/z

g

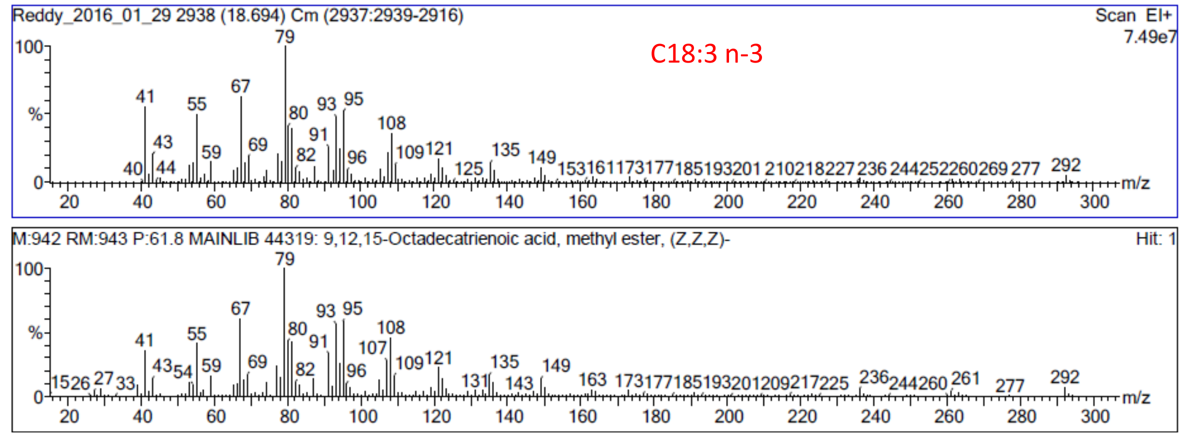
Reddy_2016_01_29 2938 (18.694) Cm (2937:2939-2916)
Scan EI+
7.49e7
C18:3 n-3
M:942 RM:943 P:61.8 MAINLIB 44319: 9,12,15-Octadecatrienoic acid, methyl ester, (Z,Z,Z)-
Hit: 1
m/z

h

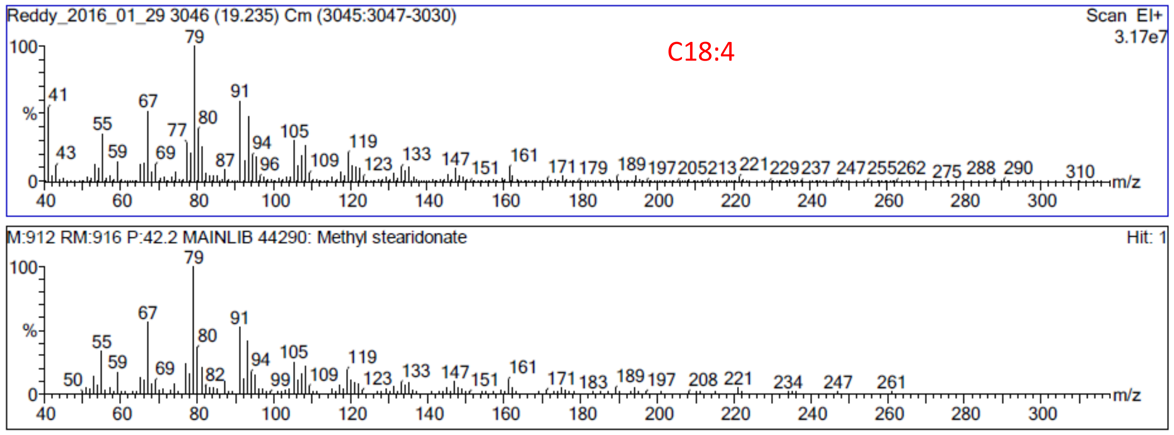
Reddy_2016_01_29 3046 (19.235) Cm (3045:3047-3030)
Scan EI+
3.17e7
C18:4
M:912 RM:916 P:42.2 MAINLIB 44290: Methyl stearidonate
Hit: 1
m/z

Supplementary Fig. S2: Distribution of transcripts encoding transcription factors involved in lipid metabolism.

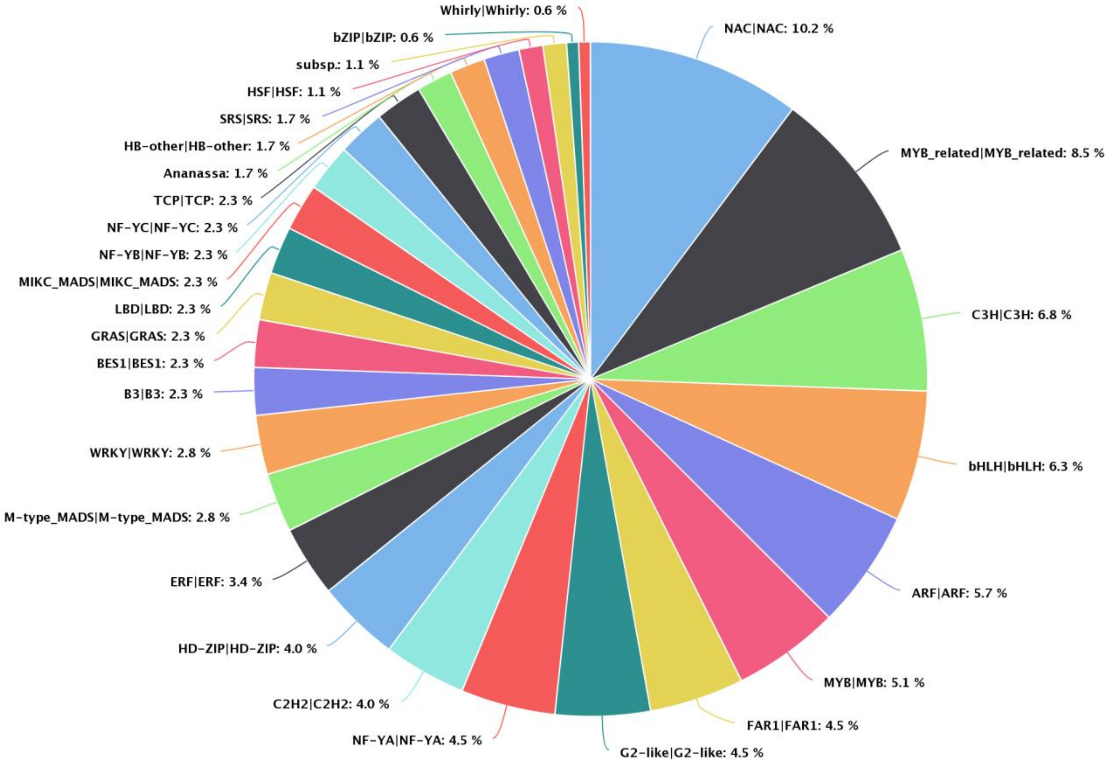

Supplementary Fig. S3: Validation of transcriptome assembly by PCR amplification. cDNA pooled from five different developing stages of the seed was used as a template to amplify lipid genes. The amplified products were analysed using 0.8% agarose gel electrophoresis. (a) M = 1 Kb DNA ladder (0.5 Kb – 10 Kb; New England Biolabs; cat no. N3232S). Lane 1: FDH (1141 bp); Lane 2: URI (938 bp); Lane 3: GAT (1208 bp); Lane 4: ADK2 (1223 bp); Lane 5: 4CL3 (1846 bp); Lane 6: HSD1 (1164 bp); Lane 7: PCNA2 (894 bp); Lane 8: DGAT2 (1111 bp); Lane 9: ALD (1224 bp); Lane 10: ADK1 (837 bp); Lane 11: cICDH (1218 bp) Lane 12: APX3 (1070 bp); Lane 13: RAS (706 bp); Lane 14: SQS1 (1348 bp); Lane 15: GAPC1 (1329 bp); Lane 16: PANC (1040 bp); Lane 17: FAD2 (1291 bp); Lane 18: RCA (1453 bp); Lane 19: AT (1360 bp); Lane 20: ACT11 (1235 bp); Lane 21: CAL (886 bp); Lane 22: MDAR1 (1338 bp); Lane 23: FLDH (1085 bp); Lane 24: D6D (1350 bp). (b) Lane 25: OLE (441 bp). M = 1 Kb DNA ladder (0.5 Kb – 10 Kb; New England Biolabs; cat no. N3232S).

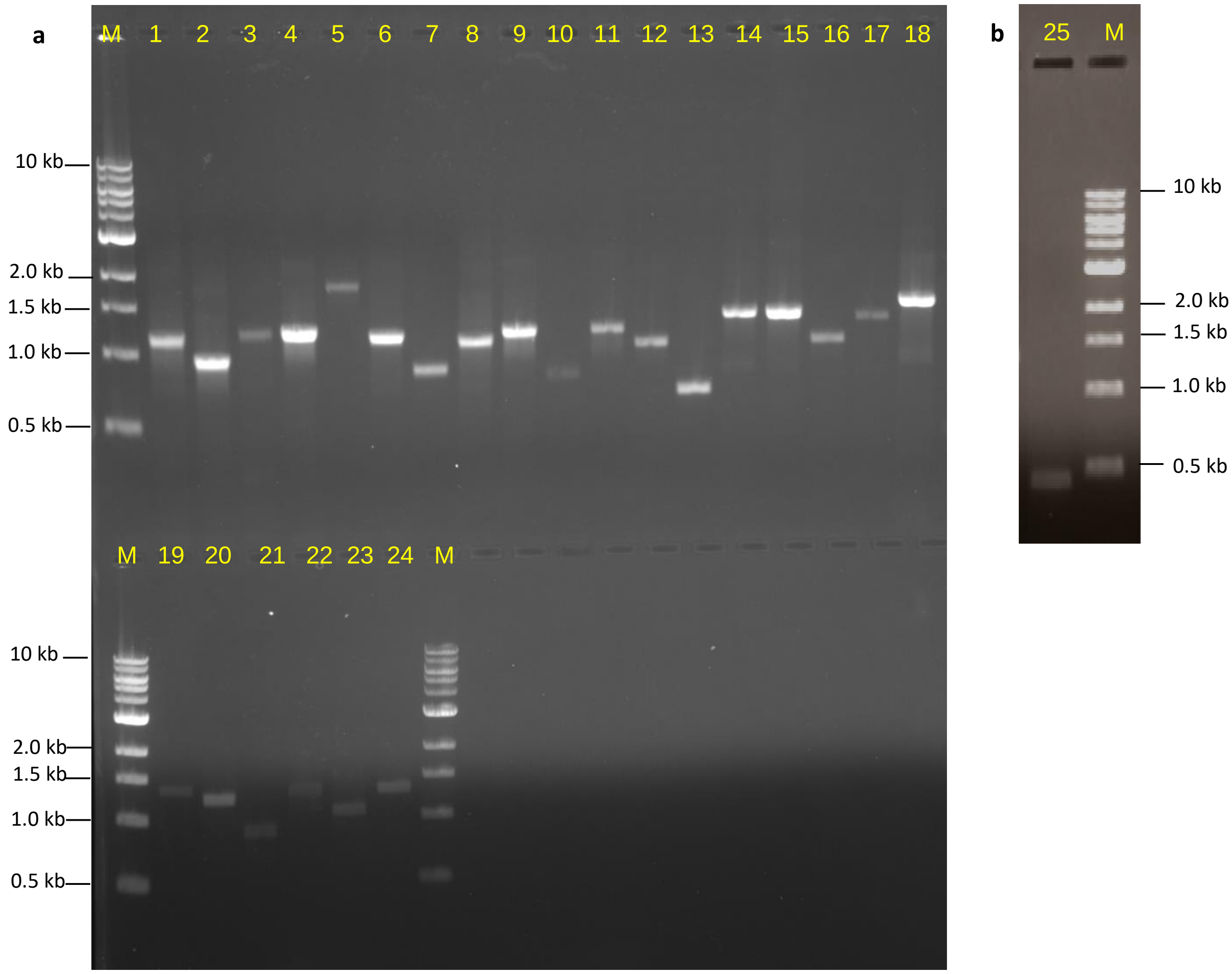

Supplementary Fig. S4: (a) Whole *B. arvensis* plant. (b) Flowers. (c) Developing seeds. (d) Mature seeds.

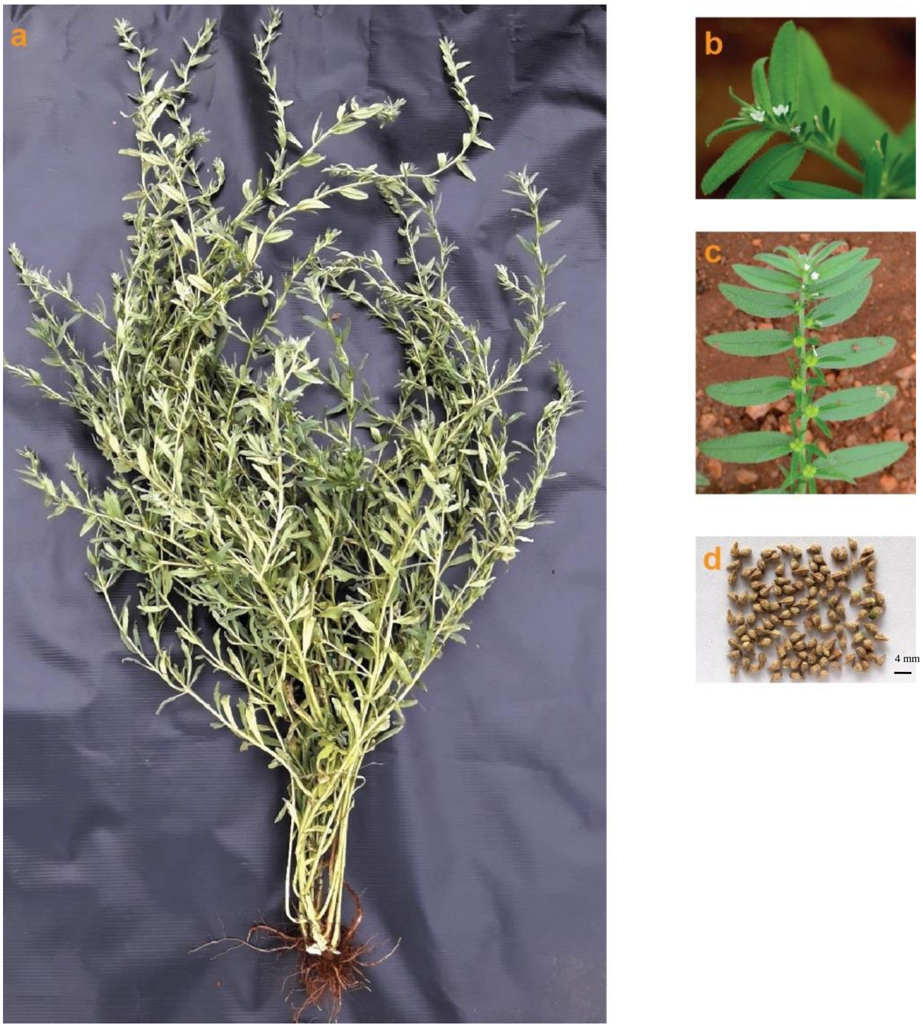

Supplementary Fig. S5: Specificity of primer pairs for qPCR amplification of candidate lipid genes. Pooled cDNA was used as a template for PCR amplification. PCR products of expected size (single band) are shown on a 2.0% agarose gel. M = 100 bp DNA ladder (100 bp -1517 bp; New England Biolabs; cat no. N3231S). Lane 1: GPAT (120 bp); Lane 2: LPAAT (106 bp); Lane 3: PAH2 (120 bp); Lane 4: CPT (112 bp); Lane 5: PDCT (106 bp); Lane 6: PDAT1 (115 bp); Lane 7: PDAT2 (106 bp); Lane 8: DGAT2 (109 bp); Lane 9: LPCAT (120 bp); Lane 10: OLE (101 bp); Lane 11: Alpha-Tubulin (107 bp); Lane 12: Clathrin Adaptor Complex (114 bp); Lane 13: SAD (101 bp); Lane 14: FAD2 (101 bp); Lane 15: FAD3 (118 bp); Lane 16: FAD7/8 (120 bp); Lane 17: D6D1/2: (100 bp); Lane 18: DGAT1 (108 bp); Lane 19: WRI1 (117 bp). (b) Lane 20: FAD6 (111 bp). M = 100 bp DNA ladder (100 bp -1517 bp; New England Biolabs; cat no. N3231S).

a

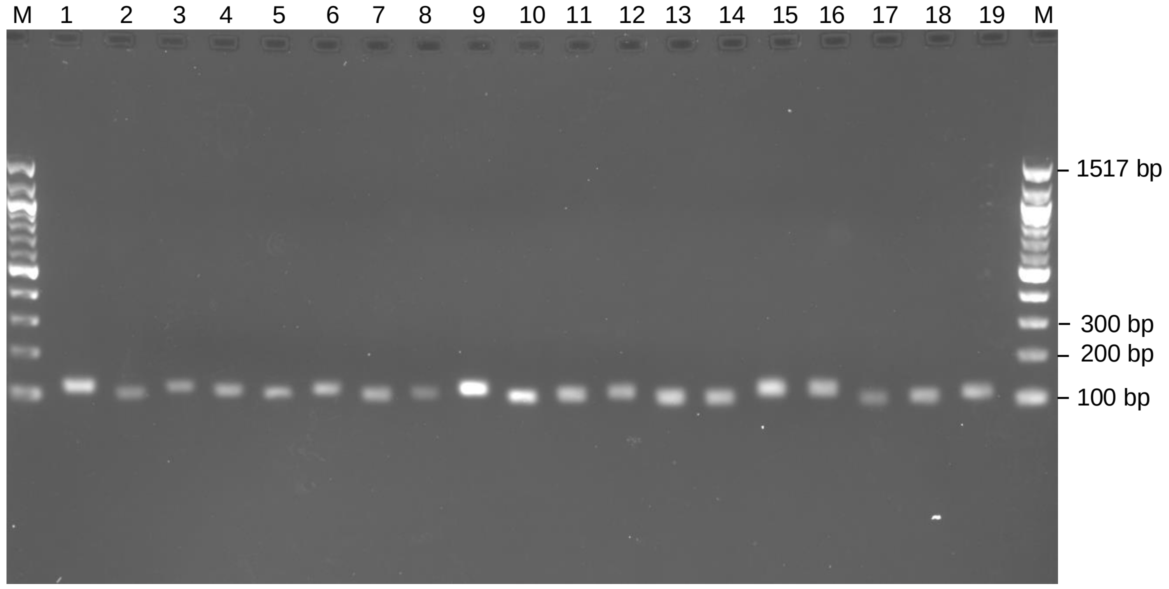

b

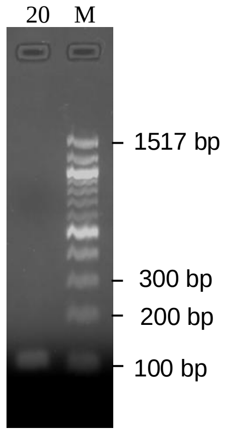

Supplement: Supplementary file 1 — Supplementary Information [file 41598_2017_9882_MOESM1_ESM.pdf]
