# Supplementary material for: Unravelling a stearidonic acid-rich triacylglycerol biosynthetic pathway in the developing seeds of Buglossoides arvensis: A transcriptomic landscape
Source: Sci Rep. 2017 Sep 5;7:10473. doi: 10.1038/s41598-017-09882-y (PMC5585386; doi:10.1038/s41598-017-09882-y)
Supplement: Supplementary file 7 — Supplementary Data 6 [file 41598_2017_9882_MOESM7_ESM.doc]

### Supplementary Data 6: Sequence alignment between assembled transcripts and sanger sequence

**Formate Dehydrogenase (FDH)**

FDH TRANSCIPT CCACTCTTTCATGGCGATGAAGCGTGTTGCTGTAACTGCGATCAAAGCCTTTGCTGCTCC

FDH SANGERS ------------------------------------------------------------

FDH TRANSCIPT TTCTCGTCACTTTCATAGTGGTATTGGAACCAAGAAGATAGTTGGAGTCTTCTACAATGG

FDH SANGERS --------------------GTATTGGAACCAAGAAGATAGTTGGAGTCTTCTACAATGG

****************************************

FDH TRANSCIPT AAATGAGTACGCTGCAAAGAATCCCAATTTCGTCGGGTGTGTGGAGAATGCATTAGGTAT

FDH SANGERS AAATGAGTACGCTGCAAAGAATCCCAATTTCGTCGGGTGTGTGGAGAATGCATTAGGTAT

************************************************************

FDH TRANSCIPT ACGTGAATGGTTGGAATCACAGGGTCACCAGTACATAGTTACAGCTGACAAAGAAGGGCC

FDH SANGERS ACGTGAATGGTTGGAATCACAGGGTCACCAGTACATAGTTACAGCTGACAAAGAAGGGCC

************************************************************

FDH TRANSCIPT AGACTGTGAGCTTGAGAAGCACATGGAAGATCTTCACGTGTTGGTAACCACCCCCTTCCA

FDH SANGERS AGACTGTGAGCTTGAGAAGCACATGGAAGATCTTCACGTGTTGGTAACCACCCCCTTCCA

************************************************************

FDH TRANSCIPT TCCAGCCTATGTCACTGCTGAAAGAATCAACAGGGCAAAGAATTTGAAGTTGTTGCTTAC

FDH SANGERS TCCAGCCTATGTCACTGCTGAAAGAATCAACAGGGCAAAGAATTTGAAGTTGTTGCTTAC

************************************************************

FDH TRANSCIPT GGCTGGAATTGGCTCAGATCACATTGATCTCAAAGCTGCAGCTGCAGCCGGATTGACAGT

FDH SANGERS GGCTGGAATTGGCTCAGATCACATTGATCTCAAAGCTGCAGCTGCAGCCGGATTGACAGT

************************************************************

FDH TRANSCIPT GGCAGAGGTCACTGGGAGCAATACTGTTTCAGTTGCAGAAGATGAATTAATGAGAATTCT

FDH SANGERS GGCAGAGGTCACTGGGAGCAATACTGTTTCAGTTGCAGAAGATGAATTAATGAGAATTCT

************************************************************

FDH TRANSCIPT CATTCTGGTTCGAAACTTTCTGCCTGGTTACCATCAGGTAACTCAAGGGGAATGGAATGT

FDH SANGERS CATTCTGGTTCGAAACTTTCTGCCTGGTTACCATCAGGTAACTCAAGGGGAATGGAATGT

************************************************************

FDH TRANSCIPT TGCAGGTGTTGCATATAGAGCTTATGATCTCGAAGGCAAGACCATTGGAACAGTTGGTGC

FDH SANGERS TGCAGGTGTTGCATATAGAGCTTATGATCTCGAAGGCAAGACCATTGGAACAGTTGGTGC

************************************************************

FDH TRANSCIPT TGGTCGCATTGGAAGGCTTTTGCTACAAAGGCTGAAACCTTTCAACTGTAATTTATTGTA

FDH SANGERS TGGTCGCATTGGAAGGCTTTTGCTACAAAGGCTGAAACCTTTCAACTGTAATTTATTGTA

************************************************************

FDH TRANSCIPT TCATGATCGTCTAAAGATAACCCCTGAATTGGAGAAAGAGACTGGGGCAACATTCGAAGA

FDH SANGERS TCATGATCGTCTAAAGATAACCCCTGAATTGGAGAAAGAGACTGGGGCAACATTCGAAGA

************************************************************

FDH TRANSCIPT AGACCTTGATGCAATGCTCCCAAAATGTGATATTATAGTCATCAATACACCTCTCACGGA

FDH SANGERS AGACCTTGATGCAATGCTCCCAAAATGTGATATTATAGTCATCAATACACCTCTCACGGA

************************************************************

FDH TRANSCIPT GAAAACAATAGGGATGTTTAACAAGGAGAAAATTTCAAAGCTAAAGAAGGGTGTTTTAAT

FDH SANGERS GAAAACAATAGGGATGTTTAACAAGGAGAAAATTTCAAAGCTAAAGAAGGGTGTTTTAAT

************************************************************

FDH TRANSCIPT TGTTAACAATGCCCGAGGAGCAATCATGGACACACAAGCAGTGGTAGATGCCTGTTCTAG

FDH SANGERS TGTTAACAATGCCCGAGGAGCAATCATGGACACACAAGCAGTGGTAGATGCCTGTTCTAG

************************************************************

FDH TRANSCIPT TGGACACATTGCAGGCTACAGTGGAGATGTCTGGTATCCACAACCAGCTCCGAAGGACCA

FDH SANGERS TGGACACATTGCAGGCTACAGTGGAGATGTCTGGTATCCACAACCAGCTCCGAAGGACCA

************************************************************

FDH TRANSCIPT CCCATGGCGTTACATGCCGAACCATGCCATGACTCCTCATTTTTCTGGTACCACCATTGA

FDH SANGERS CCCATGGCGTTACATGCCGAACCATGCCATGACTCCTCATTTTTCTGGTACCACCATTGA

************************************************************

FDH TRANSCIPT TGCTCAGCTTCGCTATGCCGCTGGTGTGAAGGACATGCTGCAGAAGTACTTCAAGGGAGA

FDH SANGERS TGCTCAGCTTCGCTATGCCGCTGGTGTGAAGGACATGCTGCAGAAGTACTTCAAGGGAGA

************************************************************

FDH TRANSCIPT GGAGTTTCCAGTAGAAAACTACATTGTCAAAGATGGGGAGCTAGCAAGTCAATACCGTTGA

FDH SANGERS -------------------------------------------------------------

**Uricase (URI)**

URI TRANSCRIPT CGTGAGATATGGCGGAGACAATAAATGGGGGGTTGAAGTTCGAGCAGAGACACGGGAAGT

URI SANGERS ---------------------------------------TCGAGCAGAGACACGGGAAGT

*********************

URI TRANSCRIPT CTAGGGTTAGAGTAGGAAGAGTATGGAAACTCCCAAACGGATCTCACTTTTTCGCCGAAT

URI SANGERS CTAGGGTTAGAGTAGGAAGAGTATGGAAACTCCCAAACGGATCTCACTTTTTCGCCGAAT

************************************************************

URI TRANSCRIPT GGAAAGTCAACGTCAGCCTCCTCTCCGATTGCGTTGCCTCCTATCTCCACCAGGATAACT

URI SANGERS GGAAAGTCAACGTCAGCCTCCTCTCCGATTGCGTTGCCTCCTATCTCCACCAGGATAACT

************************************************************

URI TRANSCRIPT CCGATATCGTTGCTACCGATACCATCAAGAACACCGTTTATGTAAAAGCTAAAGAGTGCT

URI SANGERS CCGATATCGTTGCTACCGATACCATCAAGAACACCGTTTATGTAAAAGCTAAAGAGTGCT

************************************************************

URI TRANSCRIPT CTGAGCAGGTGACTGTGGAAGATTTTGCCATCAAACTGGCCCAGCATTTTACAAGTTTCT

URI SANGERS CTGAGCAGGTGACTGTGGAAGATTTTGCCATCAAACTGGCCCAGCATTTTACAAGTTTCT

************************************************************

URI TRANSCRIPT ATCAGCAGGTAACAACGGCCATAGTCAATATTGTGGAGAAGCCGTGGGAGCGCGTATCTG

URI SANGERS ATCAGCAGGTAACAACGGCCATAGTCAATATTGTGGAGAAGCCGTGGGAGCGCGTATCTG

************************************************************

URI TRANSCRIPT TAAATGGCCAGCCTCACAAACATGGTTTCAAGCTCGGTTCTGAGAAGCACACAACAGAAG

URI SANGERS TAAATGGCCAGCCTCACAAACATGGTTTCAAGCTCGGTTCTGAGAAGCACACAACAGAAG

************************************************************

URI TRANSCRIPT TCATATTCAGTAAATTAGGCACGTTGCAAGTGAATTCTGGTATTGAAGGGTTGGCACTAC

URI SANGERS TCATATTCAGTAAATTAGGCACGTTGCAAGTGAATTCTGGTATTGAAGGGTTGGCACTAC

************************************************************

URI TRANSCRIPT TGAAGACAACACAGTCGGGCTTTGAAGGGTTTATTAGAGACAAATACACCATTTTGCCAG

URI SANGERS TGAAGACAACACAGTCGGGCTTTGAAGGGTTTATTAGAGACAAATACACCATTTTGCCAG

************************************************************

URI TRANSCRIPT AGACACGAGAAAGGATGTTGGCAACGGAGGTCACCATCTCTTGGAAGTACAATTACAACT

URI SANGERS AGACACGAGAAAGGATGTTGGCAACGGAGGTCACCATCTCTTGGAAGTACAATTACAACT

************************************************************

URI TRANSCRIPT CTGTGTCAAGTCTCCCTGTGAAGCCATTGTACTTTTCAGAAAGATACATGGATGTGAAGA

URI SANGERS CTGTGTCAAGTCTCCCTGTGAAGCCATTGTACTTTTCAGAAAGATACATGGATGTGAAGA

************************************************************

URI TRANSCRIPT AAGCTTTAGTTGACACTTTCTTTGGTTCTCCAAATGAGGGAGTTTATAGCCCATCTGTTC

URI SANGERS AAGCTTTAGTTGACACTTTCTTTGGTTCTCCAAATGAGGGAGTTTATAGCCCATCTGTTC

************************************************************

URI TRANSCRIPT AGAGGACTCTGTATCTTATGGGAAAGGCCGTTCTTGGCAGATTTCCTGATATATCCTCGG

URI SANGERS AGAGGACTCTGTATCTTATGGGAAAGGCCGTTCTTGGCAGATTTCCTGATATATCCTCGG

************************************************************

URI TRANSCRIPT TCCACTTGAAGATGCCAAATATACATTTTCTACCAGTTAACTTATCAAGCAAAGACAACC

URI SANGERS TCCACTTGAAGATGCCAAATATACATTTTCTACCAGTTAACTTATCAAGCAAAGACAACC

************************************************************

URI TRANSCRIPT CAGAAATTGTCAAGTTTGCAGATGATGTCTATTTACCAACAGATGAACCACACGGATCTA

URI SANGERS CAGAAATTGTCAAGTTTGCAGATGATGTCTATTTACCAACAGATGAACCACACGGATCTA

************************************************************

URI TRANSCRIPT TCGAGGCCAGACTAAGCCGCGTTCAGTCCAAAATGTGA

URI SANGERS TCGAGGCCAGAC--------------------------

************

**Class I glutamine amidotransferase-like superfamily protein (GAT)**

GAT TRANSCRIPT CCTACCATAGTCTAGAAGTGTCACATATTTTCATAGTCCGAAAAATGGTTGATAAAAGAG

GAT SANGERS ------------------------------------------------------------

GAT TRANSCRIPT TTCTTTTACTATGTGGAGACTATGTCGAAGATTACGAGGTGATGGTACCATTTCAAGCTT

GAT SANGERS ----TTTACTATGTGGAGACTATGTCGAAGATTACGAGGTGATGGTACCATTTCAAGCTT

********************************************************

GAT TRANSCRIPT TGCTAGCATATGGTGTCGCTGTTGATGCAGTGTGTCCTGGGAAGAAGGCGGGTGATGTTT

GAT SANGERS TGCTAGCATATGGTGTAGCTGTTGATGCAGTGTGTCCTGGGAAGAAGGCGGGTGATGTTT

**************** *******************************************

GAT TRANSCRIPT GCCGCACTGCTATTCATCAGCTATCTGTTCACCAGACTTACTCTGAATCAAGAGGTCACA

GAT SANGERS GCCGCACTGCTATTCATCAGCTATCTGTTCACCAGACTTACTCTGAATCAAGAGGTCACA

************************************************************

GAT TRANSCRIPT ACTTCACCCTTAATGCCACATTTGATGAAGTTAATGCTACTAAGTACGATGGGCTTGTAA

GAT SANGERS ACTTCACCCTTAATGCCACATTTGATGAAGTTAATGCTACTAAGTACGATGGGCTTGTAA

************************************************************

GAT TRANSCRIPT TACCTGGAGGAAGAGCACCAGAATACCTTTCCATGGATCAATCTGTCCTGGATCTGGTTA

GAT SANGERS TACCTGGAGGAAGAGCACCAGAATACCTTTCCATGGATCAATCTGTCCTGGATCTGGTTA

************************************************************

GAT TRANSCRIPT CGAAATTTTTCAAGTTGGAAAAGGCAGTTGCTTCGATCTGTCATGGGCAATTGATCTTGG

GAT SANGERS CGAAATTTTTCAAGTTGAAAAAGGCAGTTGCTTCGATCTGTCATGGGCAATTGATCTTGG

***************** ******************************************

GAT TRANSCRIPT CTGCAGCAGGGTTAGTTAAAGGTCGGAGGTGCACTGCTTATCCTGCTGTGGGACCTGCAT

GAT SANGERS CTGCAGCAGGGTTAGTTAAAGGTCGGAAGTGCACTGCTTATCCTGCTGTGGGACCTGCAT

*************************** ********************************

GAT TRANSCRIPT TGATTGCCGCAGGTGCCCACTGGATAGAACCGAAGACAGCGGCTTCATGCACCGTTGATG

GAT SANGERS TGATTGCCGCAGGTGCCCATTGGATAGAACCGAAGACAGCGGCTTCATGCACCGTTGATG

******************* ****************************************

GAT TRANSCRIPT GGAATCTTATAACTGGAGCTACATATGAAGGGCATCCTGAGTTCATCAACCTTTTCATCA

GAT SANGERS GGAATCTTATAACTGGAGCTACATATGAAGGGCATCCTGAGTTCATCAACCTTTTCATCA

************************************************************

GAT TRANSCRIPT AGGCTTTGGGGGGAAATATAAGTGATTCTGGTAAAAAGATTCTCTTTCTCTGTGGGGACT

GAT SANGERS AGGCTTTGGGGGGAAATATAAGTGATTCTGGTAAAAAGATTCTCTTTCTCTGTGGGGACT

************************************************************

GAT TRANSCRIPT ACATGGAAGACTATGAAGTCATGGTTCCTTTTCAGTCCCTTCAAGCTCTCGACTGCCATG

GAT SANGERS ACATGGAAGACTATGAAGTCATGGTTCCTTTTCAGTCCCTTCAAGCTCTCGACTGCCATG

************************************************************

GAT TRANSCRIPT TTGATGCCGTTTGTCCCAAGAAAAGTGCTGGTGATAAATGCCCAACAGCTGTCCATGATT

GAT SANGERS TTGATGCCGTTTGTCCCAAGAAAAGTGCTGGTGATAAATGCCCAACAGCTGTCCATGATT

************************************************************

GAT TRANSCRIPT TTGAGGGTGATCAAACTTATTCTGAAAAACCAGGGCATGATTTCACCCTGACTGCAGACT

GAT SANGERS TTGAGGGTGATCAAACTTATTCTGAAAAACCAGGGCATGATTTCACCCTGACTGCAGACT

************************************************************

GAT TRANSCRIPT TTAATGACGTTGAAGATTCCATGTATCACGGTCTTGTAATTCCTGGAGGACGAGCTCCAG

GAT SANGERS TTAATGACGTTGAAGATTCCATGTATCACGGTCTTGTAATTCCTGGAGGACGAGCTCCAG

************************************************************

GAT TRANSCRIPT AGTATTTGGCACTAGATGAAGGCGTTATTAGATTGGTGAAGCAATTTATGGAGTCTGGGA

GAT SANGERS AGTATTTGGCACTAGATGAAGGCGTTATTAGATTGGTGAAGCAATTTATGGAGTCTGGGA

************************************************************

GAT TRANSCRIPT AACCAGTGGCTTCTATCTGCCATGGACAACAAATATTGGCTGCTGCTGGTGTTCTAAAGG

GAT SANGERS AACCAGTGGCTTCTATCTGCCATGGACAACAAATATTGGCTGCTGCTGGTGTTCTAAAGG

************************************************************

GAT TRANSCRIPT GTAAAAAGTGTACCGCATACCCGGCTGTTAAACTCAATGTAGTTCTTGCTGGGGCAACAT

GAT SANGERS GTAAAAAGTGTACCGCATACCCGGCTGTTAAACTCAATGTAGTTCTTGCTGGGGCAACAT

************************************************************

GAT TRANSCRIPT GGTTAGAACCCGAACCAATAGACCGTTGCTTCACTGATGGTAATCTGGTTACTGGAGCAG

GAT SANGERS GGTTAGAACCCGAACCAATAGACCGTTGCTTCACTGATGGTAATCTGGTTACTGGAGCAG

************************************************************

GAT TRANSCRIPT CCTGGCCAGGTCATCCGGAGTTCATTTCTCAGTTTATGGCTCTTCTTGGTGTGCGCGTCT

GAT SANGERS C-----------------------------------------------------------

*

GAT TRANSCRIPT CATTTTAA

GAT SANGERS --------

**Adenosine kinase 2 (ADK2)**

ADK2 TRANSCRIPT CTTGAGACGAGTGATTCAACACACACACTATATAACCAACCCTCCATCTCACACTTCCAA

ADK2 SANGERS ------------------------------------------------------------

ADK2 TRANSCRIPT ATCTTGTAGCTTCTCATTTCTCCATTTTTCTTTAAATTATTATAACCTTAACAATTAAAT

ADK2 SANGERS ------------------------------------------------------------

ADK2 TRANSCRIPT TAAATATGGCAAATCTTGATGGCATTTTGTTGGGAATGGGAAATCCTTTATTGGATATTT

ADK2 SANGERS --AATATGGCAAATCTTGATGGCATTTTGTTGGGAATGGGAAATCCTTTATTGGACATTT

***************************************************** ****

ADK2 TRANSCRIPT CCTCTGTTGTTGACCAAGAATTCTTGGACAAATATGATATCAAGCTGAACAATGCAATCC

ADK2 SANGERS CCTCTGTTGTTGACCAAGAATTCTTGGACAAATATGATATCAAGCTGAACAATGCAATCC

************************************************************

ADK2 TRANSCRIPT TTGCAGAGGAGAAACATGTGCCCATGTATGATGAAATGGCATCTAAATTCAAGGTCGAGT

ADK2 SANGERS TTGCAGAGGAGAAACACGTGCCCATGTATGATGAAATGGCGTCTAAATTCAAGGTCGAGT

**************** *********************** *******************

ADK2 TRANSCRIPT TCATTGCTGGAGGTGCTACTCAGAACTCGATCAAAGTTGCACAGTGGATGCTTCAAACTC

ADK2 SANGERS TCATTGCTGGAGGTGCTACTCAGAACTCGATCAAAGTTGCACAGTGGATGCTTCAAACTC

************************************************************

ADK2 TRANSCRIPT CTGGTGCAACTGGTTACATTGGATGCATTGGAAAGGATAAGTTTGGGGAGGAAATGAAGA

ADK2 SANGERS CTGGTGCAACTGGTTACATTGGATGCATTGGAAAGGATAAGTTTGGGGAGGAAATGAAGA

************************************************************

ADK2 TRANSCRIPT AAGATTCAACGGCAGCCGGTGTTAATGTTCATTACTATGAAGATGAGACCACTCCCACTG

ADK2 SANGERS AAGATTCAACGGCAGCCGGTGTTAATGTTCATTACTATGAAGATGAGACCACTCCCACTG

************************************************************

ADK2 TRANSCRIPT GTACTTGTGCTGTTTGTGTCCTTGGTGGAGAAAGGTCTCTTGTTGCCAACTTGGCTGCTG

ADK2 SANGERS GTACTTGTGCTGTTTGTGTCCTTGGTGGAGAAAGGTCTCTTGTTGCCAACTTGGCTGCTG

************************************************************

ADK2 TRANSCRIPT CAAATTGCTACAAATCTGAACATTTGAAGAAACCTGAGAATTGGGCATTGGTTGAAAAGG

ADK2 SANGERS CAAATTGCTACAAATCTGAACATTTGAAGAAACCTGAGAATTGGGCATTGGTTGAAAAGG

************************************************************

ADK2 TRANSCRIPT CCAAGTACTACTACATTGCTGGATTTTTCCTCACTGTTTCCCCAGAGTCTATTCAGCTTG

ADK2 SANGERS CCAAGTTCTACTACATTGCTGGATTTTTCCTCACTGTTTCCCCAGAGTCTATTCAGCTTG

****** *****************************************************

ADK2 TRANSCRIPT TCGCTGAGCATGCAGCTGCAAATAACAAGATTTTCACTATGAATCTTTCGGCTCCATTTA

ADK2 SANGERS TCGCTGAGCATGCAGCTGCAAATAACAAGATTTTCACTATGAATCTTTCGGCTCCATTTA

************************************************************

ADK2 TRANSCRIPT TCTGTGAATTCTTTAGGGATCCACAGGAGAAGGCTCTTCCGTTCGTTGACATTGTCTTTG

ADK2 SANGERS TCTGTGAATTCTTTAGGGATCCACAGGAGAAGGCTCTTCCGTTCGTTGACATTGTCTTTG

************************************************************

ADK2 TRANSCRIPT GAAATGAAACAGAAGCGGTAACTTTTGCAAAAGTTCATGGGTGGGAGACTGAAAACGTTC

ADK2 SANGERS GAAATGAAACAGAAGCGGTAACTTTTGCAAAAGTTCATGGGTGGGAGACTGAAAACGTTC

************************************************************

ADK2 TRANSCRIPT AAGAAATTGCTGTGAAAATATCCCAGTGGCCAAAGTCATCTGGAACACACAAAAGGATGA

ADK2 SANGERS AAGAAATTGCTGTGAAAATATCCCAGTGGCCAAAGTCATCTGGAACACACAAAAGGATGA

************************************************************

ADK2 TRANSCRIPT CTGTAATTACCCAGGGTCACGATCCCGTTGTTGTTGCTGAGGCTGATGGGAAGGTGAAAT

ADK2 SANGERS CTGTAATTACCCAGGGTCACGATCCCGTTGTTGTTGCTGAGGCTGATGGGAAGGTGAAAT

************************************************************

ADK2 TRANSCRIPT TGTTCCCTGTCATCCCTCTGCCTAAAGAGAAACTTGTTGATACTAACGGTGCAGGTGATG

ADK2 SANGERS TGTTCCCTGTCATCCCTCTGCCTAAAGAGAAACTTGTTGATACTAACGGTGCAGGTGATG

************************************************************

ADK2 TRANSCRIPT CTTTCGTTGGAGGATTTCTGGCTCAGCTGGTTCAAGAGAAGCCTATCGAGGATTGTGTCA

ADK2 SANGERS CTTTCGTTGGAGGATTTCTGGCTCAATTGGTTCAAGAGAAGCCTATCGAGGATTGTGTCA

************************* *********************************

ADK2 TRANSCRIPT AGGCTGGTTGTTATGCGGCAAATGTAGTCATCCAGAGGTCTGGGTGCACATATCCAGAGA

ADK2 SANGERS AGGCTGGTTGTTATGCGGCAAATGTAGTCATCCAGAGGTCTGGGTGCACATATCCAGAGA

************************************************************

ADK2 TRANSCRIPT AGCCCGACTTCAAATAAGGGATAGTTTTACAATTATTCCGACTTGAACAATAATCTTGGA

ADK2 SANGERS AGCCCGACTTCAAATAAGGGATAGTTTTACAATTATTCCGACTTGAACAAT---------

***************************************************

ADK2 TRANSCRIPT TTTGTTTGAACGCCATATTACCG

ADK2 SANGERS -----------------------

**Monodehydroascorbate reductase 1 (MDAR1)**

MDAR1 TRANSCRIPT TGTCCGATCAATATTATGGCTGAGCAATCATTCAAGTACGTCATCCTCGGCGGCGGTGTT

MDAR1 SANGERS ----------------------------------AGTACGTCATCCTCGGCGGCGGTGTT

**************************

MDAR1 TRANSCRIPT GCTGCTGGGTACGCTGCTAGGGAATTTGCCAAGCAAGGAGCTAAGCCTGGTGAAGTCGCA

MDAR1 SANGERS GCTGCTGGGTACGCTGCTAGGGAATTTGCCAAGCAAGGAGCTAAGCCTGGTGAAGTCGCA

************************************************************

MDAR1 TRANSCRIPT ATTATTTCCAAAGAGGCGGTGGCTCCTTATGAACGTCCTGCTCTTAGCAAGGCATACCTC

MDAR1 SANGERS ATTATTTCCAAAGAGGCGGTGGCTCCTTATGAACGTCCTGCTCTTAGCAAGGCATACCTC

************************************************************

MDAR1 TRANSCRIPT TTTCCTGAAGGAACAGCAAGGCTTCCAGGTTTCCATACGTGTGTTGGAAGTGGAGGGGAG

MDAR1 SANGERS TTTCCTGAAGGAACAGCAAGGCTTCCAGGTTTCCATACGTGTGTTGGAAGTGGAGGGGAG

************************************************************

MDAR1 TRANSCRIPT AGATTGCTTCCTGAGTGGTACACTGAGAAGGGGGTATCATTGATCCTTAGCACAGAGATA

MDAR1 SANGERS AGATTGCTTCCTGAGTGGTACACTGAGAAGGGGGTATCATTGATCCTTAGCACAGAGATA

************************************************************

MDAR1 TRANSCRIPT GTGAAAGCTGATCTCGCATCTAAGACTCTTACTAGTGCATCAGGAGAAACATTCAAGTAC

MDAR1 SANGERS GTGAAAGCTGATCTCGCATCTAAGACTCTTACTAGTGCATCAGGAGAAACATTCAAGTAC

************************************************************

MDAR1 TRANSCRIPT GAGATCTTGATCATTGCAACTGGTGCCACAGTTCTAAAGTTGACAGATTTTGGTGTGCAA

MDAR1 SANGERS GAGATCTTGATCATTGCAACTGGTGCCACAGTTCTAAAGTTGACAGATTTTGGTGTGCAA

************************************************************

MDAR1 TRANSCRIPT GGGGCCGACTCCAAAAACATCCTTTACCTAAGAGAAATCGATGATGCCGATAAACTTGTG

MDAR1 SANGERS GGGGCCGACTCCAAAAACATCCTTTACCTAAGAGAAATCGATGATGCCGATAAACTTGTG

************************************************************

MDAR1 TRANSCRIPT GAAGCAATCAAGGCAAAGAAGGGTGGAAAGGCTGTTGTTGTTGGAGGGGGATACATTGGT

MDAR1 SANGERS GAAGCAATCAAGGCAAAGAAGGGTGGAAAGGCTGTTGTTGTTGGAGGGGGATACATTGGT

************************************************************

MDAR1 TRANSCRIPT CTTGAGCTTAGTGCAGCACTCAAGATCAATAACTATGACGTTAGCATGGTTTATCCTGAG

MDAR1 SANGERS CTTGAGCTTAGTGCAGCACTCAAGATCAATAACTATGACGTTAGCATGGTTTATCCTGAG

************************************************************

MDAR1 TRANSCRIPT CCTTGGTGCATGCCCAGACTTTTCACAGCTGACATAGCTTCATTCTATGAGGGGTATTAT

MDAR1 SANGERS CCTTGGTGCATGCCCAGACTTTTCACAGCTGACATAGCTTCATTCTATGAGGGGTATTAT

************************************************************

MDAR1 TRANSCRIPT GAAAACAAGGGAATTAGTATCATTAAAGGCACAGTAGCTGTAGGATTTGAGTCTAATGAC

MDAR1 SANGERS GAAAACAAGGGAATTAGTATCATTAAAGGCACAGTAGCTGTAGGATTTGAGTCTAATGAC

************************************************************

MDAR1 TRANSCRIPT AATGGAGAGGTTAAGGAAGTAAAGCTCAAGGATGGTAGAGTTCTGGAAGCCGACATAGTT

MDAR1 SANGERS AATGGAGAGGTTAAGGAAGTAAAGCTCAAGGATGGTAGAGTTCTGGAAGCCGACATAGTT

************************************************************

MDAR1 TRANSCRIPT ATTGTAGGTGTTGGGGCAAGACCTCTTACAGGATTATTCAAAGGGCAGGTTGAAGAAGAG

MDAR1 SANGERS ATTGTAGGTGTTGGGGCAAGACCTCTTACAGGATTATTCAAAGGGCAGGTTGAAGAAGAG

************************************************************

MDAR1 TRANSCRIPT AAAGGTGGAATTAAGACTGATGCGTTCTTCAAAACAAGCGTTCCTGATGTCTATGCAGTA

MDAR1 SANGERS AAAGGTGGAATTAAGACTGATGCGTTCTTCAAAACAAGCGTTCCTGATGTCTATGCAGTA

************************************************************

MDAR1 TRANSCRIPT GGAGATGTTGCCACATTCCCAATGAAAATATACAATGATGTAAGGAGAGTAGAACATGTT

MDAR1 SANGERS GGAGATGTTGCCACATTCCCAATGAAAATATACAATGATGTAAGGAGAGTAGAACATGTT

************************************************************

MDAR1 TRANSCRIPT GATCACGCTCGTAAATCTGCTGAGCAGGCCGTGAAGGCAATTTTTGCTGGAGCTTTGGGA

MDAR1 SANGERS GATCACGCTCGTAAATCTGCTGAGCAGGCCGTGAAGGCAATTTTTGCTGGAGCTTTGGGA

************************************************************

MDAR1 TRANSCRIPT GAGTCAATTGATGAGTATGAATATCTTCCATTCTTCTATTCTCGTGCCTTTGATCTGTCA

MDAR1 SANGERS GAGTCAATTGATGAGTATGAATATCTTCCATTCTTCTATTCTCGTGCCTTTGATCTGTCA

************************************************************

MDAR1 TRANSCRIPT TGGCAATTCTATGGAGACAATGTCGGTGACTCAGTGATTTTCGGTGACAACAACCCGACA

MDAR1 SANGERS TGGCAATTCTATGGAGACAATGTCGGTGACTCAGTGATTTTCGGTGACAACAACCCGACA

************************************************************

MDAR1 TRANSCRIPT TCTGCTACTCACAAATTCGGATCATACTGGATTAAAGATGGTAAGGTGGTCGGCGTATTC

MDAR1 SANGERS TCTGCTACTCACAAATTCGGATCATACTGGATTAAAGATGGTAAGGTGGTCGGCGTATTC

************************************************************

MDAR1 TRANSCRIPT CTAGAGGGCGGCACTCCTGAGGAAAACAAAGCGATAGCTAAGGTCGCCAAGGTCCAGCCT

MDAR1 SANGERS CTAGAGGGCGGCACTCCTGAGGAAAACAAAGCGATAGCTAAGGTCGCCAAGGTCCAGCCT

************************************************************

MDAR1 TRANSCRIPT CCAGCTGACAGCTTAGAGCAATTGGGAAAGGAGGGTCTCACTTTCGCCTCCAAGATCTGA

MDAR1 SANGERS CCAGCTGACAGCTTAGAG------------------------------------------

******************

MDAR1 TRANSCRIPT ATGCTGTAACCTGTAACC

MDAR1 SANGERS ------------------

**NAD(P)-binding Rossmann-fold superfamily protein (FLDH)**

FLDH TRANSCRIPT GAGTTTCCCAAGATGCAAGAAAAAGTACTCGTAACCGGGGCGTCCGGTTACCTAGGCGGT

FLDH SANGERS ---------------------------------ACCGGGGCGTCCGGTTACCTAGGCGGT

***************************

FLDH TRANSCRIPT CGTCTTTGCCGTGAATTAATAAACCAAGGCCATAAAGTCAAAGCATTCGTGCGCCGTACA

FLDH SANGERS CGTCTTTGCCGTGAATTAATAAACCAAGGCCATAAAGTCAAAGCATTCGTGCGCCGTACA

************************************************************

FLDH TRANSCRIPT AGTAACCTCTCTTTATTACCTTCTACAACCGGTCAATATGAAGGAGCTTTTGAGGTTGCC

FLDH SANGERS AGTAACCTCTCTTTATTACCTTCTACAACCGGTCAATATGAAGGAGCTTTTGAGGTTGCC

************************************************************

FLDH TRANSCRIPT TATGGTGACGTCACGGATTATGAATCGTTACTTGAAGCATGCTGTGATTGCCACGTCATA

FLDH SANGERS TATGGTGACGTCACGGATTATGAATCGTTACTTGAAGCATGCTGTGATTGCCACGTCATA

************************************************************

FLDH TRANSCRIPT TTTCATGTTGCTGCTCTAGTTGAACCTTGGCTACCTAATCCTTCTAGATTTGGCATTGTT

FLDH SANGERS TTTCATGTTGCTGCTCTAGTTGAACCTTGGCTACCTAATCCTTCTAGATTTGGCATTGTT

************************************************************

FLDH TRANSCRIPT AATGTTGGAGGACTGAAGAATGTACTGCAAGTATATAAAGAAGCTGGAACAATAAAGAAA

FLDH SANGERS AATGTTGGAGGACTGAAGAATGTACTGCAAGTATATAAAGAAGCTGGAACAATAAAGAAA

************************************************************

FLDH TRANSCRIPT ATCATTTATACGTCGTCGTATTTTGCCTTGGGTTCAACTGATGGATATGTTGCTGATGAA

FLDH SANGERS ATCATTTATACGTCGTCGTATTTTGCCTTGGGTTCAACTGATGGATATGTTGCTGATGAA

************************************************************

FLDH TRANSCRIPT AATCAAATGCATCCTGGGAAGTTCTTTTGCACCGAATATGAGAAATCAAAAGCGGTTGCT

FLDH SANGERS AATCAAATGCATCCTGGGAAGTTCTTTTGCACCGAATATGAGAAATCAAAAGCGGTTGCT

************************************************************

FLDH TRANSCRIPT GATAAGATTGCACTAGATGCTGCCTTAGAGGGGGTGCCTATCGTGCCATTGTACCCTGGA

FLDH SANGERS GATAAGATTGCACTAGATGCTGCCTTAGAGGGGGTGCCTATCGTGCCATTGTACCCTGGA

************************************************************

FLDH TRANSCRIPT GTCATATATGGACCTGGCAAAGTCACGTCTGGGAATGTGGTTGCACGTATGTTGATAGAG

FLDH SANGERS GTCATATATGGACCTGGCAAAGTCACGTCTGGGAATGTGGTTGCACGTATGTTGATAGAG

************************************************************

FLDH TRANSCRIPT CGCTTTAATGGGAGGTTACCGGGTTATATCGGCAAAGGGAATGATATATCTTCGTTTAGT

FLDH SANGERS CGCTTTAATGGGAGGTTACCGGGTTATATCGGCAAAGGGAATGATATATCTTCGTTTAGT

************************************************************

FLDH TRANSCRIPT CATGTTGACGATGTGGTCAATGGTCACATTGCGGCAATGCACAAAGGCCGGCCTGGTGAA

FLDH SANGERS CATGTTGACGATGTGGTCAATGGTCACATTGCGGCAATGCACAAAGGCCGGCCTGGTGAA

************************************************************

FLDH TRANSCRIPT AGATACCTTCTCACAGGAGAAAATGCATCGTTCAAGGAAGTTTTTGACCTAGTTGCTGCC

FLDH SANGERS AGATACCTTCTCACAGGAGAAAATGCATCGTTCAAGGAAGTTTTTGACCTAGTTGCTGCC

************************************************************

FLDH TRANSCRIPT ATGACAGAGACAAAACGACCTTCATTTAGCATTCCTTTATTTGTTATTGAAATATACGGC

FLDH SANGERS ATGACAGAGACAAAACGACCTTCATTTAGCATTCCTTTATTTGTTATTGAAATATACGGC

************************************************************

FLDH TRANSCRIPT TGGGTCTCCATTTTGATATCAAGAGTTACAGGGAAGCTTCCACTAATTAGTCCCCCGACA

FLDH SANGERS TGGGTCTCCATTTTGATATCAAGAGTTACAGGGAAGCTTCCACTAATTAGTCCCCCGACA

************************************************************

FLDH TRANSCRIPT GTGGATGTTCTCCGGCATCAATGGGCGTACTCTTGTGATAAAGCCAAAGAAGAGTTGGAT

FLDH SANGERS GTGGATGTTCTCCGGCATCAATGGGCGTACTCTTGTGATAAAGCCAAAGAAGAGTTGGAT

************************************************************

FLDH TRANSCRIPT TACAAACCTAGAAGCTTGAAAGAAGGTTTGTCTGAGGTGCTCCCATGGCTGAAGAGTTTA

FLDH SANGERS TACAAACCTAGAAGCTTGAAAGAAGGTTTGTCTGAGGTGCTCCCATGGCTGAAGAGTTTA

************************************************************

FLDH TRANSCRIPT GGTCTGATAAAGTACTAATAATATGGCAATCATCATTTGGAATTAACAGGAGGTATGTAG

FLDH SANGERS GGTCTGATAAAGTACTA-------------------------------------------

*****************

FLDH TRANSCRIPT CACAA

FLDH SANGERS -----

**4-coumarate:CoA ligase 3 (4CL3)**

4CL3 TRANSCRIPT AGGTGTGGGAACAACAAATCCCACAAAATCAAAAAACAAACAAACAACAAAAAACATATT

4CL3 SANGERS ------------------------------------------------------------

4CL3 TRANSCRIPT CAACAATGTTGTCTGTAGCTTCCCCTGAAACTCAAAAGCAAGAGCTTTCCTCCATTTCTA

4CL3 SANGERS ----------GTCTGTAGCTTCCCCTGAAACTCAAAAGCAAGAGCTTTCCTCCATTTCTA

**************************************************

4CL3 TRANSCRIPT CCCCCCCTTCTTCCACCCCCCAAAACCAATCCTCCATTTCTGGAGATCATCATAACAACT

4CL3 SANGERS CCCCCCCTTCTTCCACCCCCCAAAACCAATCCTCCATTTCTGGAGATCATCATAACAACT

************************************************************

4CL3 TRANSCRIPT CCAATGAAACCATCATTTTCAGATCCAAACTACCTGATATACCCATCTCCAACCACCTCC

4CL3 SANGERS CCAATGAAACCATCATTTTCAGATCCAAACTACCTGATATACCCATCTCCAACCACCTCC

************************************************************

4CL3 TRANSCRIPT CTCTCCACACATACTGCTTCGAAAATGCCTCCGAATACCCCAACAGGACATGCATCATTG

4CL3 SANGERS CTCTCCACACATACTGCTTCGAAAATGCCTCCGAATACCCCAACAGGACATGCATCATTG

************************************************************

4CL3 TRANSCRIPT ATGGCAAAACTGGCAAACAATACTCGTTTTTCGAAACAGACTCAATCTGCAGGAAAGTTG

4CL3 SANGERS ATGGCAAAACTGGCAAACAATACTCGTTTTTCGAAACAGACTCAATCTGCAGGAAAGTTG

************************************************************

4CL3 TRANSCRIPT CAGCTGGATTATCAAATCTTGGCATCCAGAAAGGAGATGTGATCATGGTCCTTCTCCAAA

4CL3 SANGERS CAGCTGGATTATCAAATCTTGGCATCCAGAAAGGAGATGTGATCATGGTCCTTCTCCAAA

************************************************************

4CL3 TRANSCRIPT ACTGCCCCGAATTCGTTTTCACATTCATGGGTGCTTCCATCATAGGTGCAGTCATCACTA

4CL3 SANGERS ACTGCCCCGAATTCGTTTTCACATTCATGGGTGCTTCCATCATAGGTGCAGTCATCACTA

************************************************************

4CL3 TRANSCRIPT CTGCAAATCCTTTCTACACCACTGCAGAAATCTTCAAACAGGTCAATGTATCCAACACCA

4CL3 SANGERS CTGCAAATCCTTTCTACACCACTGCAGAAATCTTCAAACAGGTCAATGTATCCAACACCA

************************************************************

4CL3 TRANSCRIPT AACTCATCATTACTCAATCCAACTACGTCGACAAGCTCAATAACAATAACAACAACAATA

4CL3 SANGERS AACTCATCATTACTCAATCCAACTACGTCGACAAGCTCAATAACAATAACAACAACAATA

************************************************************

4CL3 TRANSCRIPT TGACAGAATCCGATAACAAGTATCCAAAACTCGGAATCGACTTCAAGGTGATCACAATTG

4CL3 SANGERS TGACAGAATCCGATAACAAGTATCCAAAACTCGGAATCGACTTCAAGGTGATCACAATTG

************************************************************

4CL3 TRANSCRIPT ATACCCCCCCGGAAAACTGCCTACCCTTTTCACAACTCACCGAAACCACCCAAGAAAACC

4CL3 SANGERS ATACCCCCCCGGAAAACTGCCTACCCTTTTCACAACTCACCGAAACCACCCAAGAAAACC

************************************************************

4CL3 TRANSCRIPT AACTTCCATCAACTCCCATCGACTCGAACGACCCGGTAGCATTACCATTTTCCTCAGGCA

4CL3 SANGERS AACTTCCATCAACCCCCATCGACTCGAACGACCCGGTAGCATTACCATTTTCCTCAGGCA

************* **********************************************

4CL3 TRANSCRIPT CCACAGGGCTACCAAAGGGTGTGATCCTAACGCACAAAAGCCTGATCACTAGCGTTGCAC

4CL3 SANGERS CCACAGGGCTACCAAAGGGTGTGATCCTAACGCACAAAAGCCTGATCACTAGCGTTGCAC

************************************************************

4CL3 TRANSCRIPT AACAAGTAGATGGAGACAACCCGAACTTGTACCTAAAGCATGATGATGTTGTACTTTGCG

4CL3 SANGERS AACAAGTAGATGGAGACAACCCGAACTTGTACCTAAAGCATGATGATGTTGTACTTTGCG

************************************************************

4CL3 TRANSCRIPT TACTTCCATTGTTCCATATATACTCCCTAAATTCAGTGCTTCTATGTTCCTTGAGAGCTG

4CL3 SANGERS TACTTCCATTGTTCCATATATACTCCCTAAATTCAGTGCTTCTATGTTCCTTGAGAGCTG

************************************************************

4CL3 TRANSCRIPT GAGCAGCTGTATTGATCATGCAGAAATTTGAGATAGGGGCATTGTTGGAACTTATACAAA

4CL3 SANGERS GAGCAGCTGTATTGATCATGCAGAAATTTGAGATAGGGGCATTGTTGGAACTTATACAAA

************************************************************

4CL3 TRANSCRIPT GTCATCGAGTGTCGGTGGCGGCGGTGGTGCCGCCGCTAGTATTGGCGTTGGCAAAGAATC

4CL3 SANGERS GTCATCGAGTGTCGGTGGCGGCGGTGGTGCCGCCGCTAGTATTGGCGTTGGCAAAGAATC

************************************************************

4CL3 TRANSCRIPT CGATGGTGGATAAATATGATCTGAGTTCGATTAGGGTGGTGCTCTCCGGGGCGGCGCCGC

4CL3 SANGERS CGATGGTGGATAAATATGATCTGAGTTCGATTAGGGTGGTGCTCTCCGGGGCGGCGCCGC

************************************************************

4CL3 TRANSCRIPT TGGGGAAGGAGTTGGAACTAGCGTTACTTAATAGAGTCCCACATGCAATTTTTGGGCAGG

4CL3 SANGERS TGGGGAAGGAGTTGGAACTAGCGTTACTTAATAGAGTCCCACATGCAATTTTTGGGCAGG

************************************************************

4CL3 TRANSCRIPT GCTATGGCATGACTGAAGCTGGACCAGTACTATCAATGTCCCCTTCATTTGCAAAGCACC

4CL3 SANGERS GCTATGGCATGACTGAAGCTGGACCAGTACTATCAATGTCCCCTTCATTTGCAAAGCACC

************************************************************

4CL3 TRANSCRIPT CATACCCAGCAAAATCAGGGTCATGTGGAACTGTAGTTAGAAATGCTGACCTCAAGGTTA

4CL3 SANGERS CATACCCAGCAAAATCAGGGTCATGTGGAACTGTAGTTAGAAATGCTGACCTCAAGGTTA

************************************************************

4CL3 TRANSCRIPT TTGACCCCGAAACTGGTTCCTCCCTCGGCCGAAACCAACCTGGAGAAATTTGCATTCGTG

4CL3 SANGERS TTGACCCCGAAACTGGTTCCTCCCTCGGCCGAAACCAACCTGGAGAAATTTGCATTCGTG

************************************************************

4CL3 TRANSCRIPT GAGAACAGATCATGAAAGGCTATCTCAATGACCCCGAGGCAACAGCTAGGACGGTTGACA

4CL3 SANGERS GAGAACAGATCATGAAAGGCTATCTCAATGACCCCGAGGCAACAGCTAGGACGGTTGACA

************************************************************

4CL3 TRANSCRIPT TCGAGGGGTGGCTCCATACCGGTGACATTGGCTATGTGGACGACGATGATGAAGTGTTTA

4CL3 SANGERS TCGAGGGGTGGCTCCATACCGGTGACATTGGCTATGTGGACGACGATGATGAAGTGTTTA

************************************************************

4CL3 TRANSCRIPT TTGTTGACAGGGTGAAGGAACTCATCAAATTCAAGGGGTTCCAAGTTCCACCAGCTGAGC

4CL3 SANGERS TTGTTGACAGGGTGAAGGAACTCATCAAATTCAAGGGGTTCCAAGTTCCACCAGCTGAGC

************************************************************

4CL3 TRANSCRIPT TTGAGGCTCTCCTCATCTCCCACACCAACATTGCTGATGCTGCTGTTGTACCGCAAAAAG

4CL3 SANGERS TTGAGGCTCTCCTCATCTCCCACACCAACATTGCTGATGCTGCTGTTGTACCGCAAAAAG

************************************************************

4CL3 TRANSCRIPT ATGCTGATGCTGGAGAAGTCCCTGTTGCATTTGTGGTTCCTTCTAATGATGGCTTTGAAT

4CL3 SANGERS ATGCTGATGCTGGAGAAGTCCCTGTTGCATTTGTGGTTCCTTCTAATGATGGCTTTGAAT

************************************************************

4CL3 TRANSCRIPT TAACAGAAGCAGATGTCAAAGAATTTGTTTCTAAACAGGTTGTGTTCTACAAAAGGTTGC

4CL3 SANGERS TAACAGAAGCAGATGTCAAAGAATTTGTTTCTAAACAGGTTGTGTTCTACAAAAGGTTGC

************************************************************

4CL3 TRANSCRIPT ACAAGGTGTACTTTGTCCACTCTATTCCAAAGTCGCCGTCCGGCAAGATTTTGAGGAAAG

4CL3 SANGERS ACAAGGTGTACTTTGTCCACTCTATTCCAAAGTCGCCGTCCGGCAAGATTTTGAGGAAAG

************************************************************

4CL3 TRANSCRIPT ATCTCAGAGCTAAACTGGCTGCCGCGGCCTCCTCTTGAATTCTTAC

4CL3 SANGERS ATCTCAGA--------------------------------------

********

**Pantoate-beta-alanine ligase (PANC)**

PANC TRANSCRIPT GCACAAGAATATGGCAGCTAAGGAGCCATTGATCATCAGAGATAGAGATGAGATGAGAAA

PANC SANGERS ------------------------------------------------------------

PANC TRANSCRIPT ATGGTCGAGGTCCATGAGAGCCCAAGGCAGTTCAATTGGTTTAGTTCCCACCATGGGTTA

PANC SANGERS -----CGAGGTCCATGAGAGCCCAAGGCAGTTCAATTGGTTTAGTTCCCACCATGGGTTA

*******************************************************

PANC TRANSCRIPT TCTCCACGAAGGTCATATTTCCTTAGTAAAAGAAGCTCAGAAGCATACTGATCTCATAGT

PANC SANGERS TCTCCACGAAGGTCATATTTCCTTAGTAAAAGAAGCTCAGAAGCATACTGATCTCATAGT

************************************************************

PANC TRANSCRIPT TGTCTCAATTTATGTAAACCCCGGTCAATTTTCTCCAAATGAAGATCTCTCCACATACCC

PANC SANGERS TGTCTCAATTTATGTAAACCCCGGTCAATTTTCTCCAAATGAAGATCTCTCCACATACCC

************************************************************

PANC TRANSCRIPT CTCAAATTTTGAAGGTGATATTGAGAAATTGAGGTCTGTCCATGGTGGGGTTGATGTTGT

PANC SANGERS CTCAAATTTTGAAGGTGATATTGAGAAATTGAGGTCTGTCCATGGTGGGGTTGATGTTGT

************************************************************

PANC TRANSCRIPT TTTTAACCCATTCAATTTGTATGATTATGGTGTTGGTGAGGGTGGGGAGACGATGAAGAG

PANC SANGERS TTTTAACCCATTCAATTTGTATGATTATGGTGTTGGTGAGGGTGGGGAGACGATGAAGAG

************************************************************

PANC TRANSCRIPT AGGTGAAGAAGAGAAGGGTGAAAAGGTAATTTCATGTGTGGAAAGTAGCAAGTTTGGGCA

PANC SANGERS AGGTGAAGAAGAGAAGGGTGAAAAGGTAATTTCATGTGTGGAAAGTAGCAAGTTTGGGCA

************************************************************

PANC TRANSCRIPT TGAGACATGGATAAGAGTGGAAAATTTGGAGAAGGGGTTGTGTGGAAAGAGCAGACCTAT

PANC SANGERS TGAGACATGGATAAGAGTGGAAAATTTGGAGAAGGGGTTGTGTGGAAAGAGCAGACCTAT

************************************************************

PANC TRANSCRIPT TTTCGTTAGAGGTGTGGCAACTGTTGTGGCCAAGTTGTTCAATATTGTTGAGCCTGATGT

PANC SANGERS TTTCGTTAGAGGTGTGGCAACTGTTGTGGCCAAGTTGTTCAATATTGTTGAGCCTGATGT

************************************************************

PANC TRANSCRIPT TGCTGTGTTTGGGAAGAAGGACTATCAGCAATGGAGAATTATCCGTAGAATGGTTAGAGA

PANC SANGERS TGCTGTGTTTGGGAAGAAGGACTATCAGCAATGGAGAATTATCCGTAGAATGGTTAGAGA

************************************************************

PANC TRANSCRIPT TCTTGATTTTGGCATAAAAATAATTGGTTCAGAGCTTGTTCGGGAGCATGATGGACTGGC

PANC SANGERS TCTTGATTTTGGCATAAAAATAATTGGTTCAGAGCTTGTTCGGGAGCATGATGGACTGGC

************************************************************

PANC TRANSCRIPT CATGAGCTCAAGGAATGTGCACCTATCACCTCAGGAGAGGGAAAAGGCATTGTCAATAAG

PANC SANGERS CATGAGCTCAAGGAATGTGCACCTATCACCTCAGGAGAGGGAAAAGGCATTGTCAATAAG

************************************************************

PANC TRANSCRIPT CCGGTCACTATATGAAGCTAAAGAGGCAGTGGAAAATGGTGAAATCGATTGCACAAAGTT

PANC SANGERS CCGGTCACTATATGAAGCTAAAGAGGCAGTGGAAAATGGTGAAATCGATTGCACAAAGTT

************************************************************

PANC TRANSCRIPT GAGAAATAATGTTGCCCAATCCATTCAAAAAGCTGGTGGAAACGTAGATTATAATGAGAT

PANC SANGERS GAGAAATAATGTTGCCCAATCCATTCAAAAAGCTGGTGGAAACGTAGATTATAATGAGAT

************************************************************

PANC TRANSCRIPT TGTGGATCAAGAAAGTCTAGAAAGTGTGGAAGTGATACAGAGACCAGTTGTTTTTTGTAT

PANC SANGERS TGTGGATCAAGAAAGTCTAGAAAGTGTGGAAGTGATACAGAGACCAGTTGTTTTTTGTAT

************************************************************

PANC TRANSCRIPT TGCTGCATTGTTTGGAAACGTCAGGCTGATCGATAACATGGAAATTAATGTTTAACAAGG

PANC SANGERS TGCTGCATTGTTTGGAAACGTCAGGCTGATCGATAACATGGAAATTAATGTTTAACAAGG

************************************************************

PANC TRANSCRIPT AGTTTTGCCCCATCTCTACCATCTTTAGCTTCTATTGCTCTGTTTCGTGAGGGTGTCTTG

PANC SANGERS AGTTTTGCCCCATCTCTACCATCTTTAGCTTCTATTGCTCTGTTTCGTGAGGGTGTCT--

**********************************************************

PANC TRANSCRIPT TCGAAAACTCAGATTCAGCC

PANC SANGERS --------------------

**Proliferating cell nuclear antigen 2 (PCNA2)**

PCNA TRANSCRIPT CCCTCTTCGAAAATGTTGGAACTAAGGCTAGTACAAGGAAGTCTCCTTAAGAAGGTTCTT

PCNA SANGERS --------------GTTGGAACTAAGGCTAGTACAAGGAAGTCTCCTTAAGAAGGTTCTT

**********************************************

PCNA TRANSCRIPT GAATCGATCAAGGACTTAGTCAACGATGCTAACTTTGACTGCTCCTCAACTGGCTTCGCT

PCNA SANGERS GAATCGATCAAGGACTTAGTCAACGATGCTAACTTTGACTGCTCCTCAACTGGCTTCGCT

************************************************************

PCNA TRANSCRIPT TTGCAAGCAATGGACAGTAGCCATGTGGCGCTGGTGGCGTTACTTCTTAGAGCGGAGGGG

PCNA SANGERS TTGCAAGCAATGGACAGTAGCCATGTGGCGCTGGTGGCGTTACTTCTTAGAGCGGAGGGG

************************************************************

PCNA TRANSCRIPT TTCGAGCATTATCGATGCGATAGGAATATGTCGATGGGGATGAATTTAGGGAATATGGCG

PCNA SANGERS TTCGAGCATTATCGATGCGATAGGAATATGTCGATGGGGATGAATTTAGGGAATATGGCG

************************************************************

PCNA TRANSCRIPT AAGATGATGAAGTGTGCGGGGAATGATGATATTATTACGCTTAAAGCTGATGATGGGAGT

PCNA SANGERS AAGATGATGAAGTGTGCGGGGAATGATGATATTATTACGCTTAAAGCTGATGATGGGAGT

************************************************************

PCNA TRANSCRIPT GATACTGTTACTTTCATGTTTGAAAGTCCTACTCAAGACAAGATTGCAGACTTTGAGATG

PCNA SANGERS GATACTGTTACTTTCATGTTTGAAAGTCCTACTCAAGACAAGATTGCAGACTTTGAGATG

************************************************************

PCNA TRANSCRIPT AAGCTTATGGACATCGACAGTGAGCATCTTGGGATTCCAGAAGCAGAGTATCATGCTATC

PCNA SANGERS AAGCTTATGGACATCGACAGTGAGCATCTTGGGATTCCAGAAGCAGAGTATCATGCTATC

************************************************************

PCNA TRANSCRIPT GTAAGGATGCCTTCAGCAGAATTTGCTAGAATTTGTAAAGATCTGAGCAGTATTGGTGAT

PCNA SANGERS GTAAGGATGCCTTCAGCAGAATTTGCTAGAATTTGTAAAGATCTGAGCAGTATTGGTGAT

************************************************************

PCNA TRANSCRIPT ACAGTTGTGATATCCGTTACCAAGGAAGGAGTGAAGTTCTCTACTAGAGGTGACATTGGA

PCNA SANGERS ACAGTTGTGATATCCGTTACCAAGGAAGGAGTGAAGTTCTCTACTAGAGGCGACATTGGA

************************************************** *********

PCNA TRANSCRIPT GCTGCAAATATCGTTTGCCGACAAAACACAACTGTTGATAAGCCTGAGGATTCAACCATA

PCNA SANGERS GCTGCAAATATCGTTTGCCGACAAAACACAACTGTTGATAAGCCTGAGGATTCAACCATA

************************************************************

PCNA TRANSCRIPT ATAGAGATGAATGAACCTGTCTCGTTAACTTTTGCTCTGAGATACCTCAACTCCTTCACC

PCNA SANGERS ATAGAGATGAATGAACCTGTCTCGTTAACTTTTGCTCTGAGATACCTCAACTCCTTCACC

************************************************************

PCNA TRANSCRIPT AAAGCTACCCCTCTGTCTGGTACAGTTACCATAAGCTTGTCTTCAGAACTTCCCGTGGTT

PCNA SANGERS AAAGCTACCCCTCTGTCTGGTACAGTTACCATAAGCTTGTCTTCAGAACTTCCCGTGGTT

************************************************************

PCNA TRANSCRIPT GTGGAGTACAAGATTGCTGAGATGGGTTACATAAGGTTCTATTTGGCCCCTAAGATTGAA

PCNA SANGERS GTGGAGTACAAGATTGCTGAGATGGGTTACATAAGGTTCTATTTGGCCCCTAAGATTGAA

************************************************************

PCNA TRANSCRIPT GAGGACGATGAAGGGACACAACCTTGAGCCTCGTCTATGAAAGTTTGAATCGTTTTCCTG

PCNA SANGERS GAGGATGATGAAGGGACACAACCTTGAGCCTCGTCTATGAAAGTTTGAATCGTTTTCCTG

***** ******************************************************

PCNA TRANSCRIPT GTACCGGTGGATGGTTTTGGGCAGAACATGTTGTGAAGATCCGTTTCATATGTG

PCNA SANGERS GTACCGGTGGATGGTTTTGGGCAGAACATGTTGTGAA-----------------

*************************************

**Aldolase superfamily protein (ALD)**

ALD TRANSCRIPT GATCCCACACTCTACTGTCAGTGAATTGAAAATGTCAGCTACTTTGCAATCTCCGCTCTC

ALD SANGERS -----------------------------------------CTTTGCAATCTCCGCTCTC

*******************

ALD TRANSCRIPT AACTATCCTCTCTTCTTCACCTCTTCAGGAGAAGAAGCTGAGATTTGCTTCACAATCCGT

ALD SANGERS AACTATCCTCTCTTCTTCACCTCTTCAGGAGAAGAAGCTGAGATTTGCTTCACAATCCGT

************************************************************

ALD TRANSCRIPT GAAGCCAGCGGCTTTGATCATCAAGCCGCTGCGAGCTCTTTCTCTTGTGCGTGGTGCTAC

ALD SANGERS GAAGCCAGCGGCTTTGATCATCAAGCCGCTGCGAGCTCTTTCTCTTGTGCGTGGTGCTAC

************************************************************

ALD TRANSCRIPT CGGCTTCTCGTCTTCTACTGACACTGTTCCAAGTGCTGAATTAGATGCTGTGACGAAATA

ALD SANGERS CGGCTTCTCGTCTTCTACTGACACTGTTCCAAGTGCTGAATTAGATGCTGTGACGAAATA

************************************************************

ALD TRANSCRIPT CAGCGAGATTGTTCCAGACACAGTTATCTTCGATGATTTCGAGAGGTTTCCTCCAACAGC

ALD SANGERS CAGCGAGATTGTTCCAGACACAGTTATCTTCGATGATTTCGAGAGGTTTCCTCCAACAGC

************************************************************

ALD TRANSCRIPT CGCTACTGTTAGCTCGTCCTTGGTATTAGGTATATGTGGCCTTCCAGATTCTAAATTTAA

ALD SANGERS CGCTACTGTTAGCTCGTCCTTGGTATTAGGTATATGTGGCCTTCCAGATTCTAAATTTAA

************************************************************

ALD TRANSCRIPT GAGTTCTGTGGATAGAGCTTTAGCAGATTCGGAGTGTTACAATATAGAGAATCTGGCCTC

ALD SANGERS GAGTTCTGTGGATAGAGCTTTAGCAGATTCGGAGTGTTACAATATAGAGAATCTGGCCTC

************************************************************

ALD TRANSCRIPT TCGGATGTCCTGTTTCTCAGATAAGGCTATCGTGAATGTTGGAGGTGGGCTCGCAAAGCT

ALD SANGERS TCGGATGTCCTGTTTCTCAGATAAGGCTATCGTGAATGTTGGAGGTGGGCTCGCAAAGCT

************************************************************

ALD TRANSCRIPT AGTCCCTGGTCGAGTTTCTACAGAAGTCGATGCTCGTCTAGCTTATGACACCCAGGCCAT

ALD SANGERS AGTCCCTGGTCGAGTTTCTACAGAAGTCGATGCTCGTCTAGCTTATGACACCCAGGCCAT

************************************************************

ALD TRANSCRIPT AATCAAGAAGGTGCATGACCTTTTGAGATTGTATGATAAAGTTGAAGTTCCTGTTGAACG

ALD SANGERS AATCAAGAAGGTGCATGACCTTTTGAGATTGTATGATAAAGTTGAAGTTCCTGTTGAACG

************************************************************

ALD TRANSCRIPT GTTGTTGTTCAAAATTCCTTCCACCTGGCAGGGGATCGAGGCATCTAGATTACTAGAAGC

ALD SANGERS GTTGTTGTTCAAAATTCCTTCCACCTGGCAGGGGATCGAGGCATCTAGATTACTAGAAGC

************************************************************

ALD TRANSCRIPT CGAGGGAATTCAGACACACTTGACATTTGTGTTCAGCTTTTGTCAAGCAGCAGCTGCAGC

ALD SANGERS CGAGGGAATTCAGACACACTTGACATTTGTGTTCAGCTTTTGTCAAGCAGCAGCTGCAGC

************************************************************

ALD TRANSCRIPT CCAAGCTGGTGCTTCGGTCATTCAGATTTTTGTTGGTCGCCTTCGGGATTGGGCACGTAA

ALD SANGERS CCAAGCTGGTGCTTCGGTCATTCAGATTTTTGTTGGTCGCCTTCGGGATTGGGCACGTAA

************************************************************

ALD TRANSCRIPT CCATACTGGCGACACAGAAATAGATACTGCTGTAAGAAGAGGAGATGACCCTGGATTGAC

ALD SANGERS CCATACTGGCGACACAGAAATAGATACTGCTGTAAGAAGAGGAGATGACCCTGGATTGAC

************************************************************

ALD TRANSCRIPT GTTGGTGACAAAAGCTTATAACTATATTCACAAGAACGGATACAAATCAAAGCTGATGGC

ALD SANGERS GTTGGTGACAAAAGCTTATAACTATATTCACAAGAACGGATACAAATCAAAGCTGATGGC

************************************************************

ALD TRANSCRIPT AGCAGCCATAAGAAACAAACAAGATGTCTTCAATCTTTTAGGGGTTGATTACATCATAAC

ALD SANGERS AGCAGCCATAAGAAACAAACAAGATGTCTTCAATCTTTTAGGGGTTGATTACATCATAAC

************************************************************

ALD TRANSCRIPT ACCTTTGAAGATATTGCAGTCTCTCAAAGAATCCGTGACACCTCCTGATGAGAAGTACTC

ALD SANGERS ACCTTTGAAGATATTGCAGTCTCTCAAAGAATCCGTGACACCTCCTGATGAGAAGTACTC

************************************************************

ALD TRANSCRIPT TCTGGTGAGAAGGTTGTCACCAGAATCTGCTGCTGTCTACAACTTCAGTAGTGAAGAGCT

ALD SANGERS TCTGGTGAGAAGGTTGTCACCAGAATCTGCTGCTGTCTACAACTTCAGTAGTGAAGAGCT

************************************************************

ALD TRANSCRIPT TGCAAAGTGGGATCAGTATACCTTTTCCTCAGCCATGGGGCCTGCAGCTGTTGAGCTTCT

ALD SANGERS TGCAAAGTGGGATCAGTATACCTTTTCTTCAGCCATGGGGCCTGCAGCTGTTGAGCTTCT

*************************** ********************************

ALD TRANSCRIPT CGCTACCGGATTGGACGGCCACGCCAACCAATCCAGGCGGCTCGAGGAATTCTTCGGGAA

ALD SANGERS CGCTACCGGATTGGACGGCCACGCCAACCAATCCAGGCGGCTCGAGGAATT---------

***************************************************

ALD TRANSCRIPT GATATGGCCTCCTCCAAATGTGTA

ALD SANGERS ------------------------

**Adenylate kinase 1 (ADK 1)**

ADK1 TRANSCRIPT CACTCATCTAATCTCTCAACCATTTTCAGACAACAACATGGCTGCATCATCAGTTTCCCT

ADK1 SANGERS ------------------------------------------------------------

ADK1 TRANSCRIPT TGAAGATGTTCCATCAATGGACATTATGACGGAGCTTCTTCGCCGTTTCAAGTGTTCTGA

ADK1 SANGERS --AAGATGTTCCATCAATGGACATTATGACGGAGCTTCTTCGCCGTTTCAAGTGTTCTGA

**********************************************************

ADK1 TRANSCRIPT GAAGCCCGATAAACGCCTCATTCTCATTGGTCCACCTGGTTCTGGAAAGGGAACCCAATC

ADK1 SANGERS GAAGCCCGATAAACGCCTCATTCTCATTGGTCCACCTGGTTCTGGAAAGGGAACCCAATC

************************************************************

ADK1 TRANSCRIPT ACCCATAATCAAAGATGACTATTGTTTATGTCATTTGGCTACTGGTGATATGCTCAGAGC

ADK1 SANGERS ACCCATAATCAAAGATGACTATTGTTTATGTCATTTGGCTACTGGTGATATGCTCAGAGC

************************************************************

ADK1 TRANSCRIPT TGCTGTTGCAGCTAAAACTCCTCTTGGGATCAAGGCGAAAGAAGCTATGGAAAAGGGTGA

ADK1 SANGERS TGCTGTTGCAGCTAAAACTCCTCTTGGGATCAAGGCGAAAGAAGCTATGGAAAAGGGTGA

************************************************************

ADK1 TRANSCRIPT GCTTGTTTCCGATGACTTGGTTGTTGGGATTATAGACGAAGCAATGAAGAAACCCTCATG

ADK1 SANGERS GCTTGTTTCCGATGACTTGGTTGTTGGGATTATAGACGAAGCAATGAAGAAACCCTCATG

************************************************************

ADK1 TRANSCRIPT CCAGAAGGGTTTCATTCTTGATGGATTCCCTAGAACTGTGGTTCAAGCAGAAAAGCTTGA

ADK1 SANGERS CCAGAAGGGTTTCATTCTTGATGGATTCCCTAGAACTGTGGTTCAAGCAGAAAAGCTTGA

************************************************************

ADK1 TRANSCRIPT CCAGATGCTTCAGAAGCAGGGAGCTAAAGTTGATAAGGTCCTTAATTTTGCAATTCATGA

ADK1 SANGERS CCAGATGCTTCAGAAGCAGGGAGCTAAAGTTGATAAGGTCCTTAATTTTGCAATTCATGA

************************************************************

ADK1 TRANSCRIPT CTCAATCTTGGAGGAGAGGATTACTGGTCGTTGGATCCATCCATCAAGTGGCAGAACATA

ADK1 SANGERS CTCAATCTTGGAGGAGAGGATTACTGGTCGTTGGATCCATCCATCAAGTGGCAGAACATA

************************************************************

ADK1 TRANSCRIPT CCACACTAAGTTTGCACCACCCAAAGCTCCTGGAGTTGATGATGTCTCTGGAGAGCCTTT

ADK1 SANGERS CCACACTAAGTTTGCACCACCCAAAGCTCCTGGAGTTGATGATGTCTCTGGAGAGCCTTT

************************************************************

ADK1 TRANSCRIPT GATACAAAGAAAAGATGACACCAAAGAGGTTCTGAAGTCACGTCTAGATGCATTTCACAG

ADK1 SANGERS GATACAAAGAAAAGATGACACCAAAGAGGTTCTGAAGTCACGTCTAGATGCATTTCACAG

************************************************************

ADK1 TRANSCRIPT ACAAACTGAACCGGTGATAGACTACTATTCCAAGAAAGGCGTAGTCGCAAATCTCCCTGC

ADK1 SANGERS ACAAACTGAACCGGTGATAGACTACTATTCCAAGAAAGGCGTAGTCGCAAATCTCCCTGC

************************************************************

ADK1 TRANSCRIPT CGAGAAGGCTCCAAAGGAGGTAACAGCTGAAGTTCAGAAGGTTCTATCTTCTTAACAGTT

ADK1 SANGERS CGAGAAGGCTCCAAAGGAGGTAACAGCTGAAGTTCAGAAGGTTCTATCTTCTTAACAGTT

************************************************************

ADK1 TRANSCRIPT TGCTGTACTCGATAGCAGAGTTATGTGGTAATTATACATAGTCACGGGCAAACATTG

ADK1 SANGERS TGCTGTACTCGATAGCAGAGTTATGTGGTAATTATACATAGTCACG-----------

**********************************************

**cytosolic NADP+-dependent isocitrate dehydrogenase (cICDH)**

cICDH TRANSCRIPT TTCGACAAGATCAAGGTTGCTAACCCCATCGTCGAAATGGACGGAGACGAGATGACCCGT

cICDH SANGERS ------------------------------------------------------------

cICDH TRANSCRIPT GTCATTTGGCAAATGATCAAGGACAAGCTTATCTTCCCCTTTGTGGAGTTGGATATTAAG

cICDH SANGERS -TCATTTGGCAAATGATCAAGGACAAGCTTATCTTCCCCTTTGTGGAGTTGGATATTAAG

***********************************************************

cICDH TRANSCRIPT TACTTTGACCTTGGCCTTCCTCACCGTGATGCAACTGATGACAAGGTTACTGTTGAAAGC

cICDH SANGERS TACTTTGACCTTGGCCTTCCTCACCGTGATGCAACTGATGACAAGGTTACTGTTGAAAGC

************************************************************

cICDH TRANSCRIPT GCGGAAGCTACTCTGAAGTACAATGTAGCTATCAAGTGTGCGACCATCACTCCAGATGAA

cICDH SANGERS GCGGAAGCTACTCTGAAGTACAATGTAGCTATCAAGTGTGCGACCATCACTCCAGATGAA

************************************************************

cICDH TRANSCRIPT GATCGTGTAAAGGAGTTCAACTTGAAGAGCATGTGGAGGAGTCCAAATGGAACCATTAGA

cICDH SANGERS GATCGTGTAAAGGAGTTCAACTTGAAGAGCATGTGGAGGAGTCCAAATGGAACCATTAGA

************************************************************

cICDH TRANSCRIPT AATATCTTGAATGGCACCGTCTTTAGAGAACCAATTATGTGCAAAAACGTTCCTCGGCTT

cICDH SANGERS AATATCTTGAATGGCACCGTCTTTAGAGAACCAATTATGTGCAAAAACGTTCCTCGGCTT

************************************************************

cICDH TRANSCRIPT GTCCCAGGATGGACAAAGCCAATTTGCATTGGAAGGCATGCTTTTGGTGACCAATACAGA

cICDH SANGERS GTCCCAGGATGGACAAAGCCAATTTGCATTGGAAGGCATGCTTTTGGTGACCAATACAGA

************************************************************

cICDH TRANSCRIPT GCTACTGATACAGTTATCAAAGGAGCTGGCAAACTCAAATTGGTGTTTGTACCTGAGGGA

cICDH SANGERS GCTACTGATACAGTTATCAAAGGAGCTGGCAAACTCAAATTGGTGTTTGTACCTGAGGGA

************************************************************

cICDH TRANSCRIPT AGTGATGAGAAGACAGAGTTCGAGGTTTACAACTTCACAGGAGCTGGTGGAGTAGCTTTG

cICDH SANGERS AGTGATGAGAAGACAGAGTTCGAGGTTTACAACTTCACAGGAGCTGGTGGAGTAGCTTTG

************************************************************

cICDH TRANSCRIPT TCCATGTACAATACAGATGAGTCAATCCGAGCCTTTGCTGAGGCTTCAATGAACATGGCA

cICDH SANGERS TCCATGTACAATACAGATGAGTCAATCCGAGCCTTTGCTGAGGCTTCAATGAACATGGCA

************************************************************

cICDH TRANSCRIPT TACCAGAAGAAATGGCCCCTATACCTCAGCACAAAGAATACAATTCTTAAAAAATATGAT

cICDH SANGERS TACCAGAAGAAATGGCCCCTATACCTCAGCACGAAGAATACAATTCTTAAAAAATATGAT

******************************** ***************************

cICDH TRANSCRIPT GGAAGATTCAAGGACATCTTCCAAGAAGTATATGAAGCTTCATGGAAGTCTAAGTTTGAA

cICDH SANGERS GGAAGATTCAAGGACATCTTCCAAGAAGTATATGAAGCTTCATGGAAGTCTAAGTTTGAA

************************************************************

cICDH TRANSCRIPT GAAGCTGGAATATGGTATGAACATCGTCTCATTGATGATATGGTTGCTTATGCTCTGAAG

cICDH SANGERS GAAGCTGGAATATGGTATGAACATCGTCTCATTGATGATATGGTTGCTTATGCTCTGAAG

************************************************************

cICDH TRANSCRIPT AGTGATGGAGCATATGTATGGGCTTGCAAAAACTATGATGGAGATGTGCAAAGCGATTTC

cICDH SANGERS AGTGATGGGGCATATGTTTGGGCTTGCAAAAACTATGATGGAGATGTGCAAAGCGATTTC

******** ******** ******************************************

cICDH TRANSCRIPT CTAGCTCAAGGATTCGGATCTCTTGGATTGATGACATCAGTGCTGGTTTGCCCTGACGGA

cICDH SANGERS CTAGCTCAAGGATTCGGATCTCTTGGATTGATGACATCAGTGCTGGTTTGCCCTGACGGA

************************************************************

cICDH TRANSCRIPT AAGACCATAGAGGCTGAAGCTGCCCATGGAACTGTTACCCGCCACTATAGGGTTCACCAA

cICDH SANGERS AAGACCATAGAGGCTGAAGCTGCCCATGGAACTGTTACCCGCCACTATAGGGTTCACCAA

************************************************************

cICDH TRANSCRIPT AAAGGAGGTGAAACCAGCACCAACAGTATTGCCTCAATATTTGCTTGGACTAGAGGACTA

cICDH SANGERS AAAGGAGGTGAAACCAGCACCAACAGTATTGCCTCAATATTTGCTTGGACTAGAGGACTA

************************************************************

cICDH TRANSCRIPT GCGCACAGAGCAAAGTTGGATGGTAATGCTAAATTGTTGGAGTTCACTGAGAAACTAGAA

cICDH SANGERS GCGCACAGAGCAAAGTTGGATGGTAATGCTAAATTGTT-GAGTTCACTGAGAAACTAGAA

************************************** *********************

cICDH TRANSCRIPT GCCGCTTGTATTGGTACAGTCGAGTCTGGGAAGATGACCAAAGATCTTGCACTCATCATT

cICDH SANGERS GCCGCTTGTATTGGTGCAGTAGAGTCTGGGAAGATGACCAAAGATCTTGCACTCATCATT

*************** **** ***************************************

cICDH TRANSCRIPT CATGGATCCAAGCTCTCTAGGGACACATATCTGAACACTGAAGAGTTCATCGATGCTGTA

cICDH SANGERS CATGGATCCAAGCTCTCCAGGGACACATATCTGAACAC----------------------

***************** ********************

cICDH TRANSCRIPT GCAGCTGATCTGAGATCC

cICDH SANGERS ------------------

**Ascorbate peroxidase 3 (APX3)**

APX3 TRANSCRIPT GTCCATTAACATTGACCGAAAGATTGAATAGAAAGAAAGAAGCCATTAGGATTTGGCAAA

APX3 SANGERS ---------------------------------------AAGCCATTAGGATTTGGCAAA

*********************

APX3 TRANSCRIPT TAACCAGAATCACAAAAACAAATAGTAGGCACTAGGCAGGGTCCTTCCTTATCTTCGGAA

APX3 SANGERS TAACCAGAATCACAAAAACAAATAGTAGGCACTAGGCAGGGTCCTTCCTTATCTTCGGAA

************************************************************

APX3 TRANSCRIPT AATGGCGAAACCGATCGTGGACACTGAGTACCTGAAAGAGGTGGAAAGAGTTCGCCGTGA

APX3 SANGERS AATGGCGAAACCGATCGTGGACACTGAGTACCTGAAAGAGGTGGAAAGAGTTCGCCGTGA

************************************************************

APX3 TRANSCRIPT CCTCCGTGCTCTCATCTCCAACAACAACTGCTCACCCATCATGCTTCGTCTAGCATGGCA

APX3 SANGERS CCTCCGTGCTCTCATCTCCAACAACAACTGCTCACCCATCATGCTTCGTCTAGCATGGCA

************************************************************

APX3 TRANSCRIPT CGATGCTGGAACGTATGATGCTAAGACGAAGACTGGAGGTGCTAATGCTTCTATTAGGAA

APX3 SANGERS CGATGCTGGAACGTATGATGCTAAGACGAAGACTGGAGGTGCTAATGCTTCTATTAGGAA

************************************************************

APX3 TRANSCRIPT CGAACAAGAGTACTCTCACAGTTGTAACAATGGCCTCACCATTGCTCTCAACTTCTGCCA

APX3 SANGERS CGAACAAGAGTACTCTCACAGTTGTAACAATGGCCTCACCATTGCTCTCAACTTCTGCCA

************************************************************

APX3 TRANSCRIPT ACAAATCAAGGACAAGCATCCTAGAATCACTTACGCCGACCTCTTCCAGCTTGCAGGCGT

APX3 SANGERS ACAAATCAAGGACAAGCATCCTAGAATCACTTACGCCGACCTCTTCCAGCTTGCAGGCGT

************************************************************

APX3 TRANSCRIPT TGTTGCAGTTGAGGTTACAGGTGGCCCGACAATTCAGTTTCTTCCTGGAAGAAAGGACTC

APX3 SANGERS TGTTGCAGTTGAGGTTACAGGTGGCCCGACAATTCAGTTTCTTCCTGGAAGAAAGGACTC

************************************************************

APX3 TRANSCRIPT CAAGGTTTGTACTAATGAAGGACGCCTGCCTGATGCCAATAAAGGTGTGGGCCATCTGAG

APX3 SANGERS CAAGGTTTGTACTAATGAAGGACGCCTGCCTGATGCCAATAAAGGTGTGGGCCATCTGAG

************************************************************

APX3 TRANSCRIPT GGATATTTTCTATCGGATGGGTTTATCAGACAAGGATATTGTCGCATTATCAGGAGCTCA

APX3 SANGERS GGATATTTTCTATCGGATGGGTTTATCAGACAAGGATATTGTCGCATTATCAGGAGCTCA

************************************************************

APX3 TRANSCRIPT CACATTGGGAAGGGCTCATCCTGAGAGATCGGGCTTTGATGGCCCATGGACACAAGACCC

APX3 SANGERS CACATTGGGAAGGGCTCATCCTGAGAGATCGGGCTTTGATGGCCCATGGACACAAGACCC

************************************************************

APX3 TRANSCRIPT TCTGAAGTTCGATAACAGCTACTTTGTGGAGCTCCTTAAGGGGGAAAGTGAAGGCCTATT

APX3 SANGERS TCTGAAGTTCGATAACAGCTACTTTGTGGAGCTCCTTAAGGGGGAAAGTGAAGGCCTATT

************************************************************

APX3 TRANSCRIPT GAAGCTTGCTACTGATAAAGCTCTACTGGAAAATCCTGAGTTCCGCTATTACGTAGAGTT

APX3 SANGERS GAAGCTTGCTACTGATAAAGCTCTACTGGAAAATCCTGAGTTCCGCTATTACGTAGAGTT

************************************************************

APX3 TRANSCRIPT ATATGCCAAGGATGAGGATGCTTTCTTTAGAGATTATGCTGTATCACACAAGAAACTTTC

APX3 SANGERS ATATGCCAAGGATGAGGATGCTTTCTTTAGAGATTATGCTGTATCACACAAGAAACTTTC

************************************************************

APX3 TRANSCRIPT TGAACTGGGTTTTACCCCAAGTAGCTCGATTCCGAAGGTGAATGATAGCACCATTTTAGC

APX3 SANGERS TGAACTGGGTTTTACCCCAAGTAGCTCGATTCCGAAGGTGAATGATAGCACCATTTTAGC

************************************************************

APX3 TRANSCRIPT GCAAAGTGCTGTTGGAGTTGTAGTGGCTGCTGCAGTGGTGATAATTAGTTACTTGTATGA

APX3 SANGERS GCAAAGTGCTGTTGGAGTTGTAGTGGCTGCTGCAGTGGTGATAATTAGTTACTTGTATGA

************************************************************

APX3 TRANSCRIPT AGCCAGGAAAAAAATATAAGTGAACTGCCGTCTGCCACCATGTAACAGTATTAGAACATT

APX3 SANGERS AGCCAGGAAAAAAATATAAGTGAACTGCCGTCTGCCACCATGTAACAGTATTAGAACATT

************************************************************

APX3 TRANSCRIPT TATATACTGGTATTTTATATGGATTTGATAGTGTGGCAAGAAGCTTAAGA

APX3 SANGERS TATATACTGGTATTTTATATGGATTTGATAGTGTG---------------

***********************************

**Ras-related small GTP-binding family protein (RAS)**

RAS TRANSCRIPT CAATTGGTTGTTGAATGGCGGCACCACCAGCAAGGGCTCGTGCCGACTACGATTATCTTG

RAS SANGER -----------------------------------------------------------G

*

RAS TRANSCRIPT TAAAGCTCCTCCTTATTGGTGATAGTGGTGTGGGTAAGAGTTGCCTTCTGTTGCGTTTTT

RAS SANGER TAAAGCTCCTCCTTATTGGTGATAGTGGTGTGGGTAAGAGTTGCCTTCTGTTGCGTTTTT

************************************************************

RAS TRANSCRIPT CTGATGGTTCCTTCACTACTAGTTTCATTACCACAATTGGAATTGATTTTAAGATAAGAA

RAS SANGER CTGATGGTTCCTTCACTACTAGTTTCATTACCACAATTGGAATTGATTTTAAGATAAGAA

************************************************************

RAS TRANSCRIPT CAATCGAGCTTGATGGAAAGCGTATCAAGCTCCAAATTTGGGATACAGCTGGACAGGAAC

RAS SANGER CAATCGAGCTTGATGGAAAGCGTATCAAGCTCCAAATTTGGGATACAGCTGGACAGGAAC

************************************************************

RAS TRANSCRIPT GATTCCGCACCATTACTACTGCTTACTACCGTGGTGCAATGGGCATTTTGTTGGTTTATG

RAS SANGER GATTCCGCACCATTACTACTGCTTACTACCGTGGTGCAATGGGCATTTTGTTGGTTTATG

************************************************************

RAS TRANSCRIPT ATGTGACTGACGAATCATCATTTAACAATATTAAGAATTGGATACGTAACATCGAACAGC

RAS SANGER ATGTGACTGACGAATCATCATTTAACAATATTAAGAATTGGATACGTAACATCGAACAGC

************************************************************

RAS TRANSCRIPT ATGCGTCTGATAATGTTAACAAGATACTGGTAGGAAACAAGGCCGACATGGATGAAAGCA

RAS SANGER ATGCGTCTGATAATGTTAACAAGATACTGGTAGGAAACAAGGCCGACATGGATGAAAGCA

************************************************************

RAS TRANSCRIPT AAAGGGCTGTGCCTACCTCCAAGGGTCAAGCACTTGCTGATGAATACGGGATCAAATTTT

RAS SANGER AAAGGGCTGTGCCTACCTCCAAGGGTCAAGCACTTGCTGATGAATACGGGATCAAATTTT

************************************************************

RAS TRANSCRIPT TTGAGACTAGTGCAAAAACAAGTCTCAATGTGGAAAATGTTTTCTTTTCAATAGCAAGAG

RAS SANGER TTGAGACTAGTGCAAAAACAAGTCTCAATGTGGAAAATGTTTTCTTTTCAATAGCAAGAG

************************************************************

RAS TRANSCRIPT ACATAAAACAGAGGCTTGCAGACACCGACAACAAGGCAGAGCCTTCAACTATCAAAATTA

RAS SANGER ACATAAAACAGAGGCTTGCAGACACCGACAACAAGGCAGAGCCTTCAACTATCAAAATTA

************************************************************

RAS TRANSCRIPT ATCAACCTGATGCCGCTGGTGGGACGGGCCAAGCCGCCCAGAAATCTGCCTGCTGTGGTT

RAS SANGER ATCAACCTGATGCCGCTGGTGGGACGGGCCAAGCCGCCCAGAAATCTGCCTGCTGTGGTT

************************************************************

RAS TRANSCRIPT CTTAGAACTGCAAAATTTGCAAAGGGAAGGGCGTTTGTAGGTTCTA

RAS SANGER CTTAGAACTGCAAAATTTGCAAAGGGAAGGGCGTT-----------

***********************************

**Squalene synthase 1 (SQS1)**

SQS1 TRANSCRIPT ATGGGAGGGGGTAATATTATAGGGACTGTATTTTCCCATCCGGATGAGTTATTTCCACTG

SQS1 SANGER ----------------------------------------CGGATGAGTTATTTCCACTG

********************

SQS1 TRANSCRIPT GTGAAACTGAAGATGGCGGCGAAGGAAGCCGAGAAGCAGATCCCATCGGAACCCCATTGG

SQS1 SANGER GTGAAACTGAAGATGGCGGCGAAGGAAGCCGAGAAGCAGATCCCATCGGAACCCCATTGG

************************************************************

SQS1 TRANSCRIPT GGATTCTGTTATTCGATGCTTCTTAAGGTTTCGAGAAGCTTCGCACTCGTCATTCAACAG

SQS1 SANGER GGATTCTGTTATTCGATGCTTCTTAAGGTTTCGAGAAGCTTCGCACTCGTCATTCAACAG

************************************************************

SQS1 TRANSCRIPT CTCCCCACCGACCTTCGTAATGCTGTGTGCATATTTTACTTGGTTCTCCGTGCACTTGAT

SQS1 SANGER CTCCCCACCGACCTTCGTAATGCTGTGTGCATATTTTACTTGGTTCTCCGTGCACTTGAT

************************************************************

SQS1 TRANSCRIPT ACTGTAGAGGATGACACGAGTATAGCTACAGAAGTTAAGGTTCCCATTTTGATGGCTTTC

SQS1 SANGER ACTGTAGAGGATGACACGAGTATAGCTACAGAAGTTAAGGTTCCCATTTTGATGGCTTTC

************************************************************

SQS1 TRANSCRIPT CATCGTCACGTATATGATAAGGAATGGCACTTCTCTTGTGGCACAAAGGAGTACAAAGTT

SQS1 SANGER CATCGTCACGTATATGATAAGGAATGGCACTTCTCTTGTGGCACAAAGGAGTACAAAGTT

************************************************************

SQS1 TRANSCRIPT CTCATGGACCAATTCTCTATGGTTCAAACTGCTTTTCTCGAGCTTGGGACCAGTTATCAA

SQS1 SANGER CTCATGGACCAATTCTCTATGGTTCAAACTGCTTTTCTCGAGCTTGGGACCAGTTATCAA

************************************************************

SQS1 TRANSCRIPT GAGGCAATTGACGATATTACAATGAGAATGGGTGCAGGAATGGCAAAATTTATATGCAAG

SQS1 SANGER GAGGCAATTGACGATATTACAATGAGAATGGGTGCAGGAATGGCAAAATTTATATGCAAG

************************************************************

SQS1 TRANSCRIPT GAGGTAGAAACTATTAGTGATTATGATGAGTATTGTCATTATGTTGCTGGACTGGTCGGA

SQS1 SANGER GAGGTAGAAACTATTAGTGATTATGATGAGTATTGTCATTATGTTGCTGGACTGGTCGGA

************************************************************

SQS1 TRANSCRIPT TTGGGGTTGTCAAAGCTTTTTCATGCCTCTGGAAAGGAAGATTTGGTTTCCGATGATTTA

SQS1 SANGER TTGGGGTTGTCAAAGCTTTTTCATGCCTCTGGAAAGGAAGATTTGGTTTCCGATGATTTA

************************************************************

SQS1 TRANSCRIPT TCCAATTCTATGGGTTTATTTCTTCAGAAAACAAACATTATCAGAGACTATCTGGAGGAC

SQS1 SANGER TCCAATTCTATGGGTTTATTTCTTCAGAAAACAAACATTATCAGAGACTATCTGGAGGAC

************************************************************

SQS1 TRANSCRIPT ATAAATGAGATACCCAAGTCACGTATGTTTTGGCCACGCCAGATATGGAGTAAATATGTC

SQS1 SANGER ATAAATGAGATACCCAAGTCACGTATGTTTTGGCCACGCCAGATATGGAGTAAATATGTC

************************************************************

SQS1 TRANSCRIPT GACAAACTTGAGGATTTGAAATATGAACAAAACTCTGTGAAGGCTGTAGAATGTCTCAAC

SQS1 SANGER GACAAACTTGAGGATTTGAAATATGAACAAAACTCTGTGAAGGCTGTAGAATGTCTCAAC

************************************************************

SQS1 TRANSCRIPT GATATGGTTACAAATGCTCTGTCACATGTGGAAGATTGCCTGACATATATGGGTGCATTG

SQS1 SANGER GATATGGTTACAAATGCTCTGTCACATGTGGAAGATTGCCTGACATATATGGGTGCATTG

************************************************************

SQS1 TRANSCRIPT CGTGATCCTGCCATCTTTCGATTCTGTGCTATTCCGCAGATTATGGCTATTGGGACATTA

SQS1 SANGER CGTGATCCTGCCATCTTTCGATTCTGTGCTATTCCGCAGATTATGGCTATTGGGACATTA

************************************************************

SQS1 TRANSCRIPT GCTCTGTGCTACAACAACATTGAACTATTCCGAGGTGTAGTGAAAATGAGGCGTGGTCTC

SQS1 SANGER GCTCTGTGCTACAACAACATTGAACTATTCCGAGGTGTAGTGAAAATGAGGCGTGGTCTC

************************************************************

SQS1 TRANSCRIPT ACTGCGAAAGTGATAGCTCAGACCAAAGCTATGCCAGATGTTTATGGTGCTTTCTTTGAT

SQS1 SANGER ACTGCGAAAGTGATAGCTCAGACCAAAGCTATGCCAGATGTTTATGGTGCTTTCTTTGAT

************************************************************

SQS1 TRANSCRIPT TTTTCTAGAATGCTGAAGTCTAAGGTTAACGAGAATGATCCGAATTCTACAAAAACTATA

SQS1 SANGER TTTTCTAGAATGCTGAAGTCTAAGGTTAACGAGAATGATCCGAATTCTACAAAAACTATA

************************************************************

SQS1 TRANSCRIPT GATAGGCTAGATACAATAATGAAATCTTGCAGGGAGTCTGGAACCCTGGATAAAAGGCGA

SQS1 SANGER GATAGGCTAGATACAATAATGAAATCTTGCAGGGAGTCTGGAACCCTGGATAAAAGGCGA

************************************************************

SQS1 TRANSCRIPT TCTTTCATGGTTGAAAATGATTCAAGCTACAATTCAGGACTGCTTATTATCATCTTTATC

SQS1 SANGER TCTTTCATGGTTGAAAATGATTCAAGCTACAATTCAGGACTGCTTATTATCATCTTTATC

************************************************************

SQS1 TRANSCRIPT CTTTTTGCTATAATCCTGGCGTATCTACCTTTTCAGCCAAAAAATACATGATTGTATTTT

SQS1 SANGER CTTTTTGCTATAATCCTGGCGTATCTACCTTTTCAGCCAAAAAATACATGATTGTATTTT

************************************************************

SQS1 TRANSCRIPT TTGAGGAGTAAAACCTTGTGCATTGCAATGTGGTTGGACATTCAACTTCTGAAATATGTG

SQS1 SANGER TTGAGGAGTAAAACCTTGTGCATTGCAATGTGGTTGGACATTCAACTTCTGAAATA----

********************************************************

SQS1 TRANSCRIPT TAATTCAACCGTTTCATGTGGAAAGTTG

SQS1 SANGER ----------------------------

**glyceraldehyde-3-phosphate dehydrogenase C subunit 1(GAPC1)**

GAPC1 TRANSCRIPT TCTAACACCTACTACTCTCATGGGTTCAGACAAGAAGATTAAGATCGGAATTAACGGATT

GAPC1 SANGER -----------------------------------AGATTAAGATCGGAATTAACGGATT

*************************

GAPC1 TRANSCRIPT CGGAAGAATCGGTCGTTTGGTTGCTAGAGTTGCTCTACAGAGAGACGATGTTGAACTTGT

GAPC1 SANGER CGGAAGAATCGGTCGTTTGGTTGCTAGAGTTGCTCTACAGAGAGATGATGTTGAACTTGT

********************************************* **************

GAPC1 TRANSCRIPT TGCAGTCAACGATCCATTCATCACCACTGAATACATGACATATATGTTTAAGTATGACAG

GAPC1 SANGER TGCAGTCAACGATCCATTCATCACCACTGAATACATGACATATATGTTTAAGTATGACAG

************************************************************

GAPC1 TRANSCRIPT TGTTCATGGTCAATGGAAGCACAATGAACTTAAGGTTCATGATGAGAAGACCCTTCTTTT

GAPC1 SANGER TGTTCATGGTCAATGGAAGCACAATGAACTTAAGGTTCATGATGAGAAGACCCTTCTTTT

************************************************************

GAPC1 TRANSCRIPT CGGAGAAAAACCAGTACGCGTCTTTGGATTCAGGAACCCAGAGGAGATCCCATGGGGTGA

GAPC1 SANGER CGGAGAAAAACCAGTACGCGTCTTTGGATTCAGGAACCCAGAGGAGATCCCATGGGGTGA

************************************************************

GAPC1 TRANSCRIPT AACTGGTGCCGACTTTGTTGTGGAGTCCACCGGTGTATTCACTGACAAGGACAAGGCTGC

GAPC1 SANGER AACTGGTGCCGACTTTGTTGTGGAGTCCACCGGTGTATTCACCGACAAGGACAAGGCTGC

****************************************** *****************

GAPC1 TRANSCRIPT TGCTCACTTGAAGGGTGGTGCTAAGAAGGTAGTTATCTCAGCTCCTAGCAAGGACGCTCC

GAPC1 SANGER TGCTCACTTGAAGGGTGGTGCTAAGAAGGTAGTTATCTCAGCTCCTAGCAAGGACGCTCC

************************************************************

GAPC1 TRANSCRIPT CATGTTTGTTGTGGGTGTTAACCACGAAGAATACAAAGCAGATCTTGATATTGTTTCAAA

GAPC1 SANGER CATGTTTGTTGTGGGTGTTAACCACGAAGAATACAAAGCCGATCTTGATATTGTTTCAAA

*************************************** ********************

GAPC1 TRANSCRIPT CGCTAGCTGCACAACCAACTGTCTTGCGCCATTGGCTAAGGTGATCAATGATAGGTTTGG

GAPC1 SANGER CGCTAGCTGCACAACCAACTGTCTTGCGCCATTGGCTAAGGTGATCAATGATAGGTTTGG

************************************************************

GAPC1 TRANSCRIPT CATTGTGGAGGGTCTCATGACTACCGTCCACTCCATTACCGCCACTCAGAAGACTGTTGA

GAPC1 SANGER CATTGTGGAGGGTCTCATGACTACCGTCCACTCCATTACCGCCACTCAGAAGACTGTTGA

************************************************************

GAPC1 TRANSCRIPT TGGTCCATCATCGAAGGACTGGAGAGGTGGAAGGGCTGCCTCATTCAACATCATTCCCAG

GAPC1 SANGER TGGTCCATCATCGAAGGACTGGAGAGGTGGAAGGGCTGCCTCATTCAACATCATTCCCAG

************************************************************

GAPC1 TRANSCRIPT CAGCACAGGTGCTGCCAAGGCAGTCGGGAAAGTGCTTCCAGCACTTAACGGAAAATTGAC

GAPC1 SANGER CAGCACAGGTGCTGCCAAGGCAGTCGGGAAAGTGCTTCCAGCACTTAACGGAAAATTGAC

************************************************************

GAPC1 TRANSCRIPT TGGAATGTCTTTCCGTGTACCAACTGTTGATGTCTCAGTGGTTGACCTCACTGTTAGGCT

GAPC1 SANGER TGGAATGTCTTTCCGTGTACCAACTGTTGATGTCTCAGTGGTTGACCTCACTGTTAGGCT

************************************************************

GAPC1 TRANSCRIPT AGAGAAGGATGCTACCTATGAAGAAATCAAAGCCGCTATCAAGGAAGAGTCAGAAGGCAA

GAPC1 SANGER AGAGAAGGATGCTACCTATGAAGAAATCAAAGCCGCTATCAAGGAAGAGTCAGAAGGCAA

************************************************************

GAPC1 TRANSCRIPT GCTTAAAGGTATCCTGGGATACACGGAGGATGATGTCGTATCTACAGATTTTGTTGGTGA

GAPC1 SANGER GCTTAAAGGTATCCTGGGATACACGGAGGATGATGTCGTATCTACAGATTTTGTTGGTGA

************************************************************

GAPC1 TRANSCRIPT CAACAGATCAAGCATCTTCGATGCTAAGGCTGGAATCGCCTTGAACAAGAACTTCGTGAA

GAPC1 SANGER CAACAGATCAAGCATCTTCGATGCTAAGGCTGGAATCGCCTTGAACAAGAACTTCGTGAA

************************************************************

GAPC1 TRANSCRIPT GCTCGTGTCATGGTATGACAACGAATGGGGATACAGTTCTCGTGTGGTGGACCTCATCGT

GAPC1 SANGER GCTCGTGTCATGGTATGACAACGAATGGGGATACAGTTCTCGTGTGGTGGACCTCATCGT

************************************************************

GAPC1 TRANSCRIPT CCATATGGCTTCCGTTTCTGCTTAAGGCTTCTTGTTTCAACTACTACTATGAAGGCTTTT

GAPC1 SANGER CCATATGGCTTCCGTTTCTGCTTAAGGCTTCTTGTTTCAACTACTACTATGAAGGCTTTT

************************************************************

GAPC1 TRANSCRIPT TGTGGCTTCAGTTAGAGTGTTTCTAGCTGAGTTTGTTCTTTAATAAATGAGTCATATTTC

GAPC1 SANGER TGTGGCTTCAGTTAGAGTGTTTCTAGCTGAGTTTGTTCTTTAATAAATGAGTCATATTTC

************************************************************

GAPC1 TRANSCRIPT CCCCTCCCATTGCCTCCCAAGAACTTAGTTTGCATATTATACTGAGCTTTGTATGAACTC

GAPC1 SANGER CCCCTCCCATTGCCTCCCAAGAACTTAGTTTGCATATTATACTGAGCTTTGTATGAACTC

************************************************************

GAPC1 TRANSCRIPT AGGTTTTTCTTTGTTTTAATGGAGAATGAGAATATTTTCTAGTTACTTATTTTCATTTTA

GAPC1 SANGER AGGTTTTTCTTTGTTTTAATGGAGAATGAGAATATTTTCTAGTTACTTATTTTCATTTTA

************************************************************

GAPC1 TRANSCRIPT ATGTTTTAGTTGCCCTTCATCAAGTTTGTTTGACAAGAAAGTAGATGCGATGCATATTTG

GAPC1 SANGER ATGTTTTAGTTGCCCTTCATCAAGTT----------------------------------

**************************

GAPC1 TRANSCRIPT GACAGTATC

GAPC1 SANGER ---------

**Rubisco activase (RCA)**

RCA TRANSCRIPT AGCCAAAATCGACACAATCACTAGCTATGGCAGCTTCAATTATTCCAACTTCATCATTTT

RCA SANGER -----------------------------------------------ACTTCATCATTTT

*************

RCA TRANSCRIPT TTGGCACTAGTTTGAAGAAATTGAGTCCCACATTGAGTCTACCAAAGGCTTCTGGGAGCT

RCA SANGER TTGGCACTAGTTTGAAGAAATTGAGTCCCACATTGAGTCTACCAAAGGCTTCTGGGAGCT

************************************************************

RCA TRANSCRIPT TCAAGGTTTGTGCTAATGCTAGTGATCCTGGAAATGAACAAGTCCAAACAGGCAAAGATA

RCA SANGER TCAAGGTTTGTGCTAATGCTAGTGATCCTGGAAATGAACAAGTCCAAACAGGCAAAGATA

************************************************************

RCA TRANSCRIPT AATGGAAGGGTCTCGCCTACGATATCTCCGACGACCAACAAGATATTACTAGAGGGAAAG

RCA SANGER AATGGAAGGGTCTCGCCTACGATATCTCCGACGACCAACAAGATATTACTAGAGGGAAAG

************************************************************

RCA TRANSCRIPT GGATGGTGGACACCCTTTTCCAAGCTCCTGTGGGAGATGGCACTCATGTCCCTGTCCTTA

RCA SANGER GGATGGTGGACACCCTTTTCCAAGCTCCTGTGGGAGATGGCACTCATGTCCCTGTCCTTA

************************************************************

RCA TRANSCRIPT ACTCCTATGAGTACATCAGCCAGGGACTTCGCACATTGGACAACAGAACAAAGGATGGTC

RCA SANGER ACTCCTATGAGTACATCAGCCAGGGACTTCGCACATTGGACAACAGAACAAAGGATGGTC

************************************************************

RCA TRANSCRIPT TCTATATCGCCCCGGCCTTCATGGACAAACTAATTGTTCACATCACCAAGAACTTCATGA

RCA SANGER TCTATATCGCCCCGGCCTTCATGGACAAACTAATTGTTCACATCACCAAGAACTTCATGA

************************************************************

RCA TRANSCRIPT CTTTACCTAACATCAAGGTTCCTCTTATCTTGGGTATTTGGGGAGGCAAAGGTCAGGGGA

RCA SANGER CTTTACCTAACATCAAGGTTCCTCTTATCTTGGGTATTTGGGGAGGCAAAGGTCAGGGGA

************************************************************

RCA TRANSCRIPT AATCTTTCCAATGTGAGCTTGTTTTCGCCAAGATGGGAATCAACCCCATTATGATGAGTG

RCA SANGER AATCTTTCCAATGTGAGCTTGTTTTCGCCAAGATGGGAATCAACCCCATTATGATGAGTG

************************************************************

RCA TRANSCRIPT CTGGAGAACTCGAAAGCGGAAATGCAGGAGAGCCAGCAAAGCTGATCAGGCAAAGGTACC

RCA SANGER CTGGAGAACTCGAAAGCGGAAATGCAGGAGAGCCAGCAAAGCTGATCAGGCAAAGGTACC

************************************************************

RCA TRANSCRIPT GTGAGGCTGCAGATATAATCAAGAAAGGTAAAATGTGTTGCCTCTTCATCAATGATCTCG

RCA SANGER GTGAGGCTGCAGATATAATCAAGAAAGGTAAAATGTGTTGCCTCTTCATCAATGATCTCG

************************************************************

RCA TRANSCRIPT ACGCTGGTGCTGGACGTATGGGAGGCACTACACAATACACTGTCAACAACCAAATGGTGA

RCA SANGER ACGCTGGTGCTGGACGTATGGGAGGCACTACACAATACACTGTCAACAACCAAATGGTGA

************************************************************

RCA TRANSCRIPT ATGCTACTCTGATGAACATTGCTGATAGTCCAACAAATGTCCAGCTCCCTGGAATGTACA

RCA SANGER ATGCTACTCTGATGAACATTGCTGATAGTCCAACAAATGTCCAGCTCCCTGGAATGTACA

************************************************************

RCA TRANSCRIPT ACAAAGAAGAGATTCCTAGGGTGCCTATTATTGTCACTGGGAACGATTTTTCGACTCTGT

RCA SANGER ACAAAGAAGAGATTCCTAGGGTGCCTATTATTGTCACTGGGAACGATTTTTCGACTCTGT

************************************************************

RCA TRANSCRIPT ATGCTCCCCTTATCCGTGATGGCCGTATGGAGAAATTCTACTGGGCGCCTACACGAGAAG

RCA SANGER ATGCTCCCCTTATCCGTGATGGCCGTATGGAGAAATTCTACTGGGCGCCTACACGAGAAG

************************************************************

RCA TRANSCRIPT ATCGTATCGGTGTGTGTACTGGAATATTTAGGACTGATAATGTTCCTGAACAAGACATTG

RCA SANGER ATCGTATCGGTGTGTGTACTGGAATATTTAGGACTGATAATGTTCCTGAACAAGACATTG

************************************************************

RCA TRANSCRIPT TCAAGCTTGTTGATACCTTCCCTGGTCAATCCATAGATTTCTTTGGTGCTTTGAGGGCAA

RCA SANGER TCAAGCTTGTTGATACCTTCCCTGGTCAATCCATAGATTTCTTTGGTGCTTTGAGGGCAA

************************************************************

RCA TRANSCRIPT GAGTGTACGATGATGAAGTGAGGAAGTGGATCACAGAAATTGGTGTTGACAACATTGGCA

RCA SANGER GAGTGTACGATGATGAAGTGAGGAAGTGGATCACAGAAATTGGTGTTGACAACATTGGCA

************************************************************

RCA TRANSCRIPT AGAAGCTAGTGAACTCGAGGGAAGGACCACCAAAATTTGATCAACCAAAGATGACACTCA

RCA SANGER AGAAGCTAGTGAACTCGAGGGAAGGACCACCAAAATTTGATCAACCAAAGATGACACTCA

************************************************************

RCA TRANSCRIPT AGAAGCTACTTGAATATGGCAACATGCTTGTTCAAGAACAGGAGAATGTGAAGAGAGTAC

RCA SANGER AGAAGCTACTTGAATATGGCAACATGCTTGTTCAAGAACAGGAGAATGTGAAGAGAGTAC

************************************************************

RCA TRANSCRIPT AATTAGCTGACAAGTACTTGAACGAGGCCGCTCTAGGGGACGCTAATGCTGATGCCATGG

RCA SANGER AATTAGCTGACAAGTACTTGAACGAGGCCGCTCTAGGGGACGCTAATGCTGATGCCATGG

************************************************************

RCA TRANSCRIPT CCAGTGGAAACTTCTACGGGAAGGCAGCACAACAACTGAACGTTCCAGTGCCCGAAGGAT

RCA SANGER CCAGTGGAAACTTCTACGGGAAGGCAGCACAACAACTGAACGTTCCAGTGCCCGAAGGAT

************************************************************

RCA TRANSCRIPT GCACTGATCCAGGTGCAACAAACTTTGATCCAACAGCGAGGAGTGATGATGGTAGCTGTG

RCA SANGER GCACTGATCCAGGTGCAACAAACTTTGATCCAACAGCGAGGAGTGATGATGGTAGCTGTG

************************************************************

RCA TRANSCRIPT TATACAAATTTTAGGCTTATTTCTAGTGATGTTTGGAAAGAACTTCAAGCAATGTAAGGG

RCA SANGER TATACAAATTTTAGGC--------------------------------------------

****************

RCA TRANSCRIPT AGTCCAATGGTAT

RCA SANGER -------------

**Actin-11 (ACT11)**

ACTIN TRANSCRIPT ATGGCAGACACTGAGGATATCCAGCCACTCGTCTGTGACAATGGTACCGGAATGGTGAAG

ACTIN SANGER -----------------------------------------------------------G

*

ACTIN TRANSCRIPT GCTGGATTTGCTGGAGATGATGCTCCTAGGGCAGTTTTCCCAAGTATCGTGGGAAGGCCA

ACTIN SANGER GCTGGATTTGCTGGAGATGATGCTCCTAGGGCAGTTTTCCCAAGTATCGTGGGAAGGCCA

************************************************************

ACTIN TRANSCRIPT AGACACACTGGAGTTATGGTTGGTATGGGTCAGAAAGATGCCTATGTTGGTGATGAGGCT

ACTIN SANGER AGACACACTGGAGTTATGGTTGGTATGGGTCAGAAAGATGCCTATGTTGGTGATGAGGCT

************************************************************

ACTIN TRANSCRIPT CAATCCAAGAGAGGTATCTTGACCTTGAAATATCCTATTGAGCATGGTATTGTTAGCAAC

ACTIN SANGER CAATCCAAGAGAGGTATCTTGACCTTGAAATATCCTATTGAGCATGGTATTGTTAGCAAC

************************************************************

ACTIN TRANSCRIPT TGGGATGACATGGAGAAGATCTGGCATCACACCTTCTACAATGAGCTTCGTGTTGCCCCA

ACTIN SANGER TGGGATGACATGGAGAAGATCTGGCATCACACCTTCTACAATGAGCTTCGTGTTGCCCCA

************************************************************

ACTIN TRANSCRIPT GAGGAGCACCCTGTTCTTTTGACCGAGGCACCCCTCAACCCCAAGGCTAACAGGGAGAAA

ACTIN SANGER GAGGAGCACCCTGTTCTTTTGACCGAGGCACCCCTCAACCCCAAGGCTAACAGGGAGAAA

************************************************************

ACTIN TRANSCRIPT ATGACTCAAATCATGTTTGAGACATTCAACGTTCCTGCTATGTATGTTGCTATTCAGGCT

ACTIN SANGER ATGACTCAAATCATGTTTGAGACATTCAACGTTCCTGCTATGTATGTTGCTATTCAGGCT

************************************************************

ACTIN TRANSCRIPT GTTCTTTCTCTATATGCCAGTGGTCGTACTACCGGTATTGTGCTGGATTCTGGTGATGGT

ACTIN SANGER GTTCTTTCTCTATATGCCAGTGGTCGTACTACCGGTATTGTGCTGGATTCTGGTGATGGT

************************************************************

ACTIN TRANSCRIPT GTGAGTCACACTGTCCCCATCTATGAAGGATATGCCTTACCACATGCTATCCTTCGTCTT

ACTIN SANGER GTGAGTCACACTGTCCCCATCTATGAAGGATATGCCTTACCACATGCTATCCTTCGTCTT

************************************************************

ACTIN TRANSCRIPT GACCTTGCCGGGCGTGACCTTACAGAGCATCTCATGAAGATCCTTACCGAGAGAGGTTAC

ACTIN SANGER GACCTTGCCGGGCGTGACCTTACAGAGCATCTCATGAAGATCCTTACCGAGAGAGGTTAC

************************************************************

ACTIN TRANSCRIPT ATGTTCACCACCTCTGCCGAACGGGAAATTGTCCGTGACATTAAGGAGAAGCTTGCTTAT

ACTIN SANGER ATGTTCACCACCTCTGCCGAACGGGAAATTGTCCGTGACATTAAGGAGAAGCTTGCTTAT

************************************************************

ACTIN TRANSCRIPT GTGGCTCTTGACTATGAGCAGGAGCTTGACACTTCCAAGAGCAGCTCATCAATTGAGAAG

ACTIN SANGER GTGGCTCTTGACTATGAGCAGGAGCTTGACACTTCCAAGAGCAGCTCATCAATTGAGAAG

************************************************************

ACTIN TRANSCRIPT AACTACGAGTTGCCCGACGGGCAGGTTATTACAATTGGAGCCGAGAGATTCCGTTGCCCA

ACTIN SANGER AACTACGAGTTGCCCGACGGGCAGGTTATTACAATTGGAGCCGAGAGATTCCGTTGCCCA

************************************************************

ACTIN TRANSCRIPT GAGGTCCTCTTCCAGCCTTCCGTTATCGGAATGGAAGCTGCAGGAATTCACGAGACCACC

ACTIN SANGER GAGGTCCTCTTCCAGCCTTCCGTTATCGGAATGGAAGCTGCAGGAATTCACGAGACCACC

************************************************************

ACTIN TRANSCRIPT TACAACTCCATCATGAAGTGTGATGTGGATATCAGGAAGGACCTCTATGGTAACATTGTG

ACTIN SANGER TACAACTCCATCATGAAGTGTGATGTGGATATCAGGAAGGACCTCTATGGTAACATTGTG

************************************************************

ACTIN TRANSCRIPT CTCAGTGGTGGTTCGACTATGTTCCCTGGTATCGCTGACCGTATGAGCAAAGAAATCACT

ACTIN SANGER CTCAGTGGTGGTTCGACTATGTTCCCTGGTATCGCTGACCGTATGAGCAAAGAAATCACT

************************************************************

ACTIN TRANSCRIPT GCTTTGGCCCCAAGCAGCATGAAGATCAAGGTGGTTGCACCACCAGAAAGGAAGTACAGT

ACTIN SANGER GCTTTGGCCCCAAGCAGCATGAAGATCAAGGTGGTTGCACCACCAGAAAGGAAGTACAGT

************************************************************

ACTIN TRANSCRIPT GTCTGGATTGGAGGATCCATCCTTGCATCCCTCAGCACCTTCCAACAGATGTGGATTTCC

ACTIN SANGER GTCTGGATTGGAGGATCCATCCTTGCATCCCTCAGCACCTTCCAACAGATGTGGATTTCC

************************************************************

ACTIN TRANSCRIPT AAGAGCGAGTATGATGAATCTGGTCCATCAATTGTCCACAGGAAGTGCTTCTAAGGTGCT

ACTIN SANGER AAGAGCGAGTATGATGAATCTGGTCCATCAATTGTCCACAGGAAGTGCTTCTAAGGTGCT

************************************************************

ACTIN TRANSCRIPT ATTATCACCATGCTGGTGAACCTCTACTTTCTCTCTGTTAAGTTGGTTTTATTTTTTGTC

ACTIN SANGER ATTATCACCATGCTGGTGAACCTCTACTTTCTCTCTGTTAAGTTGGTTTTATTT------

******************************************************

ACTIN TRANSCRIPT TTATGTCTTGAACAAACAAAGGTTGGTTGACATGG

ACTIN SANGER -----------------------------------

**Alpha tubulin (AT)**

AT TRANSCRIPT ATGAGGGAGTGCATTTCGATCCATATTGGTCAAGCTGGTATCCAAGTTGGTAATGCTTGT

AT SANGERS ------------------------------------------------------------

AT TRANSCRIPT TGGGAACTTTACTGCCTTGAACATGGCATTCAGCCTGATGGACAAATGCCTTCAGATGCA

AT SANGERS -GGGAACTTTACTGCCTTGAACATGGCATTCAGCCTGATGGACAAATGCCTTCAGATGCA

***********************************************************

AT TRANSCRIPT ACCGTGGGTGGTGGAGATGACGCCTTCAACACGTTTTTCTCCGAAACCGGGGCTGGAAAG

AT SANGERS ACCGTGGGTGGTGGAGATGACGCCTTCAACACGTTTTTCTCCGAAACCGGGGCTGGAAAG

************************************************************

AT TRANSCRIPT CACGTCCCTCGTGCCGTGTTTGTAGATCTGGAGCCTACTGTCATTGATGAGGTCCGTACT

AT SANGERS CACGTCCCTCGTGCCGTGTTTGTAGATCTGGAGCCTACTGTCATTGATGAGGTCCGTACT

************************************************************

AT TRANSCRIPT GGTACCTACCGCCAGCTCTTCCACCCTGAGCAGCTCATCAGTGGCAAGGAGGATGCCGCT

AT SANGERS GGTACCTACCGCCAGCTCTTCCACCCTGAGCAGCTCATCAGTGGCAAGGAGGATGCCGCT

************************************************************

AT TRANSCRIPT AACAACTTCGCTCGTGGACATTATACCATTGGCAAGGAGATTGTGGACCTTTGCTTGGAT

AT SANGERS AACAACTTCGCTCGTGGACATTATACCATTGGCAAGGAGATTGTGGACCTTTGCTTGGAT

************************************************************

AT TRANSCRIPT AGGATCAGGAAGCTTGCCGACAACTGCACTGGTTTGCAAGGATTCTTGGTGTTCCATGCC

AT SANGERS AGGATCAGGAAGCTTGCCGACAACTGTACTGGTTTCCAAGGTTTCTTGGTGTTCCATGCC

************************** ******** ***** ******************

AT TRANSCRIPT GTTGGTGGTGGTACTGGATCGGGGCTTGGATCTCTTCTATTGGAGAGGCTTTCCGTTGAC

AT SANGERS GTTGGTGGTGGTACTGGATCGGGGCTTGGCTCTCTTCTATTGGAGAGGCTTTCCGTTGAC

***************************** ******************************

AT TRANSCRIPT TACGGGAAGAAATCCAAGCTTGGATTCACTATCTACCCATCTCCTCAGGTCTCAACTGCT

AT SANGERS TACGGGAAGAAATCCAAGCTTGGATTCACTATCTACCCATCTCCTCAGGTGTCAACTGCT

************************************************** *********

AT TRANSCRIPT GTTGTTGAACCTTACAACTCTGTGCTCTCAACTCACTCACTCCTTGAGCACACTGATGTT

AT SANGERS GTTGTTGAACCTTACAACTCTGTGCTCTCCACTCACTCACTCCTTGAGCACACTGATGTT

***************************** ******************************

AT TRANSCRIPT GCAGTGCTCCTTGATAATGAGGCTATTTACGATATCTGTCGCAAGTCCCTCGACATTGAG

AT SANGERS GCAGTGCTCCTTGATAATGAGGCTATTTACGATATCTGTCGCAAGTCCCTCGACATTGAG

************************************************************

AT TRANSCRIPT CGCCCAACTTACACCAATCTGAACAGGCTTGTTTCTCAGGTCATCTCTTCATTGACAACT

AT SANGERS CGCCCAACTTACACCAATCTGAACAGGCTTGTTTCTCAGGTCATCTCTTCATTGACAACT

************************************************************

AT TRANSCRIPT TCCTTGAGGTTCGATGGTGCCTTGAATGTAGATGTTAATGAGTTCCAGACCAATTTGGTG

AT SANGERS TCCTTGAGGTTCGATGGTGCCTTGAATGTAGATGTTAATGAGTTCCAGACCAATTTGGTG

************************************************************

AT TRANSCRIPT CCATACCCCAGGATTCACTTTATGCTTTCGTCATATGCACCTGTTATTTCCGCTGAGAAA

AT SANGERS CCATACCCCAGGATTCACTTTATGCTTTCGTCATATGCACCTGTTATTTCCGCTGAGAAA

************************************************************

AT TRANSCRIPT GCTTATCACGAGCAACTATCTGTTGCTGAGATAACAAACACTTCATTTGAGCCATCTTCT

AT SANGERS GCTTATCACGAGCAACTATCTGTTGCTGAGATAACAAACACTTCATTTGAGCCATCTTCT

************************************************************

AT TRANSCRIPT ATGATGGTCAAATGTGACCCTCGCCACGGGAAATACATGGCCTGCTGTCTGATGTACCGT

AT SANGERS ATGATGGTCAAATGTGACCCTCGCCACGGGAAATACATGGCCTGCTGTCTGATGTACCGT

************************************************************

AT TRANSCRIPT GGTGATGTGGTGCCTAAGGATGTGAATGCTGCTGTTGCAACCATCAAGACTAAGAGAACC

AT SANGERS GGTGATGTGGTGCCTAAGGATGTGAATGCTGCTGTTGCAACCATCAAGACTAAGAGAACC

************************************************************

AT TRANSCRIPT ATTCAGTTTGTTGATTGGTGCCCCACCGGCTTCAAGTGTGGAATCAACTATCAGCCACCA

AT SANGERS ATTCAGTTTGTTGATTGGTGCCCCACCGGCTTCAAGTGTGGAATCAACTATCAGCCACCA

************************************************************

AT TRANSCRIPT ACTGTCGTTCCTGGTGGTGACTTGGCTAAGGTGCAGAGGGCTGTTTCCATGATCTCCAAC

AT SANGERS ACTGTCGTTCCTGGTGGTGACTTGGCTAAGGTGCAGAGGGCTGTTTCCATGATCTCCAAC

************************************************************

AT TRANSCRIPT ACTACTAGTGTAGCTGAGGTGTTCTCGAGGATTGACCACAAGTTCGATCTGATGTATGCT

AT SANGERS ACTACTAGTGTAGCTGAGGTGTTCTCGAGGATTGACCACAAGTTCGATCTGATGTATGCC

***********************************************************

AT TRANSCRIPT AAGCGTGCGTTCGTGCACTGGTATGTTGGTGAGGGTATGGAGGAAGGAGAATTCTCCGAG

AT SANGERS AAGCGTGCGTTCGTGCACTGGTATGTTGGTGAGGGTATGGAGGAAGGAGAATTCTCCGAG

************************************************************

AT TRANSCRIPT GCTAGGGAGGACCTGGCTGCTCTCGAAAAGGATTACGAAGAAGTCGGTGCTGAGTCTGCC

AT SANGERS GCTAGGGAGGACCTGGCTGCTCTCGAAAAGGATTACGAAGAAGTCGGTGCTGAGTCTGCC

************************************************************

AT TRANSCRIPT GAGGGAGAGGATGATGATGGTGAAGACTACTAAGGGGAGT

AT SANGERS GAGGGAGAG-------------------------------

*********

**Diacylglycerol acyltransferase 2 (DGAT2)**

DGAT2 TRANSCRIPT TAAGATGGAGGTGAACGGCACATCGCCGCCGTCAAAGGGGTTGTCCGGCGAGGCAGCACC

DGAT2 SANGER ------------------------------------------------------------

DGAT2 TRANSCRIPT GCCGTCAACGGCGGCGGAGTTCAAAGGCGTGCAAGGCTCTTGGCCTGAAACTATTTTGGC

DGAT2 SANGER -----------CGGCGGAGTTCAAAGGCGTGCAAGGCTCTTGGCCTGAAACTATTTTGGC

*************************************************

DGAT2 TRANSCRIPT AATGATATTATGGCTTGGAGCTATTCATTTTATTGTTATAGTGATCTTGACTTCCTTCAT

DGAT2 SANGER AATGATATTATGGCTTGGAGCTATTCATTTTATTGTTATAGTGATCTTGACTTCCTTCAT

************************************************************

DGAT2 TRANSCRIPT TTTCCTCCCTTTATCCAAATTTCTAATGGTCATTGGATTGTTGGTGGTGTTTATGGTGCT

DGAT2 SANGER TTTCCTCCCTTTATCCAAATTTCTAATGGTCATTGGATTGTTGGTGGTGTTTATGGTGCT

************************************************************

DGAT2 TRANSCRIPT TCCTATAGATGAGAAAAGCAAATGGGGAACAAAATTAGCAAGATATATATGTAAGCATGC

DGAT2 SANGER TCCTATAGATGAGAAAAGCAAATGGGGAACAAAATTAGCAAGATATATATGTAAGCATGC

************************************************************

DGAT2 TRANSCRIPT TTGTGGTTACTTTCCAGTGAGCTTGTATGTAGAGGATATCAAAGCTTTCAATCCTAAAGA

DGAT2 SANGER TTGTGGTTACTTTCCAGTGAGCTTGTATGTAGAGGATATCAAAGCTTTCAATCCTAAAGA

************************************************************

DGAT2 TRANSCRIPT GGCCTATGTTTTTGGATATGAGCCCCATTCAGTGTGGCCAATTGGAGTTGTTGCACTTTC

DGAT2 SANGER GGCCTATGTTTTTGGATATGAGCCCCATTCAGTGTGGCCAATTGGAGTTGTTGCACTTTC

************************************************************

DGAT2 TRANSCRIPT CGACCATACACGTTTTCTGCCTCTTCGGGACGTTAAAGTCCTTGCAAGTTCTGCTGTGTT

DGAT2 SANGER CGACCATACACGTTTTCTGCCTCTTCGGGACGTTAAAGTCCTTGCAAGTTCTGCTGTGTT

************************************************************

DGAT2 TRANSCRIPT TTATACGCCGTTCTTGAGGCATATATGGTCATGGTTAGGGGTTACAACTGCCTCAAGGAA

DGAT2 SANGER TTATACGCCGTTCTTGAGGCATATATGGTCATGGTTAGGGGTTACAACTGCCTCAAGGAA

************************************************************

DGAT2 TRANSCRIPT AATTTTTACATCCCTTTTGTCGAATGGTTACAGCTGCATCATTGTCCCAGGTGGTGTTCA

DGAT2 SANGER AATTTTTACATCCCTTTTGTCGAATGGTTACAGCTGCATCATTGTCCCAGGTGGTGTTCA

************************************************************

DGAT2 TRANSCRIPT AGAGGCATTTTATATGGAGCGCGGTTCCGAGATTGCATTCATAAAAAGTAGAAAAGGATT

DGAT2 SANGER AGAGGCATTTTATATGGAGCGCGGTTCCGAGATTGCATTCATAAAAAGTAGAAAAGGATT

************************************************************

DGAT2 TRANSCRIPT TGTTCGCCTTGCGATGGAGATGGGCAAACCTTTAGTTCCAGTTTTCTGCTTTGGCCAGAG

DGAT2 SANGER TGTTCGCCTTGCGATGGAGATGGGCAAACCTTTAGTTCCAGTTTTCTGCTTTGGCCAGAG

************************************************************

DGAT2 TRANSCRIPT TGATGTGTACAAGTGGTGGAAACCCAGTGGAAAACTGTACTTGGATTTTTCAAGAGCTAT

DGAT2 SANGER TGATGTGTACAAGTGGTGGAAACCCAGTGGAAAACTGTACTTGGATTTTTCAAGAGCTAT

************************************************************

DGAT2 TRANSCRIPT AAAGTTCACCCCCATTTTATTCTGGGGAATTCTTGGTACACCTCTTCCTTTCCGTCGTCC

DGAT2 SANGER AAAGTTCACCCCCATTTTATTCTGGGGAATTCTTGGTACACCTCTTCCTTTCCGTCGTCC

************************************************************

DGAT2 TRANSCRIPT TTTGCATGTGGTGGTTGGTAAACCGATTCTGCTAGAAAAGAATCCGCAACCATCTATCGA

DGAT2 SANGER TTTGCATGTGGTGGTTGGTAAACCGATTCTGCTAGAAAAGAATCCGCAACCATCTATCGA

************************************************************

DGAT2 TRANSCRIPT AGAGGTGGCCGAAGTGCATGCCCAGTTCGTTGAAGCGCTTAAGGATCTTTTCGAAAAACA

DGAT2 SANGER AGAGGTGGCCGAAGTGCATGCCCAGTTCGTTGAAGCGCTTAAGGATCTTTTCGAAAAACA

************************************************************

DGAT2 TRANSCRIPT TAAAGCGAGGGTGGGTCATCCTGAACTTCCATTGAGGATCCTTTAGTAATCATGAAAATT

DGAT2 SANGER TAAAGCGAGGGTGGGTCATCCTGAACTTCCATTGAGGATCCTTTAGTAATCATGAAAATT

************************************************************

DGAT2 TRANSCRIPT TGTATGCCTGATAATGTATTATTAATATGGAAAAAATAAGTTACCCCCCCCTCCTTTCTT

DGAT2 SANGER TGTATGCCTGATAATGTATTATTAATATGGAAAAAATAAGTTACCCC-------------

***********************************************

DGAT2 TRANSCRIPT TCACTATTTTCCTTTGGCATGGTAGAACTGT

DGAT2 SANGER -------------------------------

**Fatty acid desaturase 2 (FAD2)**

FAD2 TRANSCRIPT TGAACAATGGGTGCTGGAGGCCGAATGTCGGTTGCCTCGGAGGGAAAGAAATCAAAGTCC

FAD2 SANGER ----------------------------------------------------------CC

**

FAD2 TRANSCRIPT GACGTCGTCCAACGAGTCCCGAGTGATAAGCCACCATTCACAGTTGGGGACATCAAGAAA

FAD2 SANGER GACGTCGTCCAACGAGTCCCGAGTGATAAGCCACCATTCACAGTTGGGGACATCAAGAAA

************************************************************

FAD2 TRANSCRIPT GCCATCCCACCCCATTGTTTCCAGCGATCTGTCCTGCACTCTTTCTCCTATGTTGTTTAT

FAD2 SANGER GCCATCCCACCCCATTGTTTCCAGCGATCTGTCCTGCACTCTTTCTCCTATGTTGTTTAT

************************************************************

FAD2 TRANSCRIPT GATCTGGTCATTGCCTCGGTCTTCTACTATGTTGCATCGCATTACATCCATGTCCTTCCT

FAD2 SANGER GATCTGGTCATTGCCTCGGTCTTCTACTATGTTGCATCGCATTACATCCATGTCCTTCCT

************************************************************

FAD2 TRANSCRIPT TATCCACTCTCATACATAGCGTGGCCTGTGTATTGGTTCTGCCAGGGTTGTGTTCTAACC

FAD2 SANGER TATCCACTCTCATACATAGCGTGGCCTGTGTATTGGTTCTGCCAGGGTTGTGTTCTAACC

************************************************************

FAD2 TRANSCRIPT GGTGTTTGGGTTATCGCTCATGAATGTGGTCACCATGCTTTCAGCGACTACCAATGGCTG

FAD2 SANGER GGTGTTTGGGTTATCGCTCATGAATGTGGTCACCATGCTTTCAGCGACTACCAATGGCTG

************************************************************

FAD2 TRANSCRIPT GACGACACTGTTGGCCTGGTCCTCCATTCTGCTCTCCTTGTGCCTTATTTCTCATGGAAA

FAD2 SANGER GACGACACTGTTGGCCTGGTCCTCCATTCTGCTCTCCTTGTGCCTTATTTCTCATGGAAA

************************************************************

FAD2 TRANSCRIPT TACAGTCATCGTCGCCACCACTCAAATACCGGATCTCTTGAACGTGATGAGGTATTTGTG

FAD2 SANGER TACAGTCATCGTCGCCACCACTCAAATACCGGATCTCTTGAACGTGATGAGGTATTTGTG

************************************************************

FAD2 TRANSCRIPT CCTAAAAAGAGATCAGGCGTCAGCTGGTTTTTCAAGTATCTGAATAATCCACCAGGCAGG

FAD2 SANGER CCTAAAAAGAGATCAGGCGTCAGCTGGTTTTTCAAGTATCTGAATAATCCACCAGGCAGG

************************************************************

FAD2 TRANSCRIPT GTCCTCGTTCTTTTGGTCCAACTCACCCTCGGTTGGCCTTTGTATTTGATGCTCAACGTC

FAD2 SANGER GTCCTCGTTCTTTTGGTCCAACTCACCCTCGGTTGGCCTTTGTATTTGATGCTCAACGTC

************************************************************

FAD2 TRANSCRIPT TCAGGGCGGCCTTATGATCGGTTTGCTTGCCATTTTGACCCCAAAAGTCCAATCTACAAT

FAD2 SANGER TCAGGGCGGCCTTATGATCGGTTTGCTTGCCATTTTGACCCCAAAAGTCCAATCTACAAT

************************************************************

FAD2 TRANSCRIPT AACCGTGAGCGCCTTCAGATCTTGATTTCTGATGCTGGTATTATGGCCGTCATGTATGGC

FAD2 SANGER AACCGTGAGCGCCTTCAGATCTTGATTTCTGATGCTGGTATTATGGCCGTCATGTATGGC

************************************************************

FAD2 TRANSCRIPT TTATACCGCCTTGTGGCAGCAAAAGGAATTGCTTGGTTGGTGTGTTATTACGGTGTTCCT

FAD2 SANGER TTATACCGCCTTGTGGCAGCAAAAGGAATTGCTTGGTTGGTGTGTTATTACGGTGTTCCT

************************************************************

FAD2 TRANSCRIPT TTGCTTGTGGTGAATGGTTTTCTTGTATTGATCACATATTTGCAGCACACTCACCCTTCA

FAD2 SANGER TTGCTTGTGGTGAATGGTTTTCTTGTATTGATCACATATTTGCAGCACACTCACCCTTCA

************************************************************

FAD2 TRANSCRIPT TTGCCTCACTATGATTCCTCCGAATGGGACTGGTTGAGGGGGGCGCTCGCCACAGTTGAC

FAD2 SANGER TTGCCTCACTATGATTCCTCCGAATGGGACTGGTTGAGGGGGGCGCTCGCCACAGTTGAC

************************************************************

FAD2 TRANSCRIPT AGAGACTACGGCATCCTCAACAAGGTCTTCCATAACATCACCGATACTCATGTTGCTCAC

FAD2 SANGER AGAGACTACGGCATCCTCAACAAGGTCTTCCATAACATCACCGATACTCATGTTGCTCAC

************************************************************

FAD2 TRANSCRIPT CATTTGTTCTCAACCATGCCACATTATCATGCCATGGAGGCGACGAAAGTAATCAAGCCA

FAD2 SANGER CATTTGTTCTCAACCATGCCACATTATCATGCCATGGAGGCGACGAAAGTAATCAAGCCA

************************************************************

FAD2 TRANSCRIPT TTACTAGGAGAGTACTACCAGTGTGACAGGACTCCCGTCTTCAAGGCCATGTATAGAGAA

FAD2 SANGER TTACTAGGAGAGTACTACCAGTGTGACAGGACTCCCGTCTTCAAGGCCATGTATAGAGAA

************************************************************

FAD2 TRANSCRIPT GTGAAGGAGTGCATCTACGTTGAGGCCGACGAAGGCGAGCAGAAGAAAGGAGTATTCTGG

FAD2 SANGER GTGAAGGAGTGCATCTACGTTGAGGCCGACGAAGGGGACCAGAAGAAAGGAGTATTCTGG

*********************************** ** *********************

FAD2 TRANSCRIPT TACAAAAACAAGCTCTAAATTTCCAAAATGGAAAAAGTGGGAAGATGCTAATGAGCATAC

FAD2 SANGER TACAAAAACAAGCTCTAAATTTCGAAAATGGAAAAAGTGGGAAGATGCTAATGAGCATAC

*********************** ************************************

FAD2 TRANSCRIPT TCCCCTTTCTTGTAAGGTAGGTTTGTTGTGTTTCTTGTTATGAAACTTTGGTTGTTGAAA

FAD2 SANGER TCCCCTTTCTTGTAAGGTAGGTTTGTTGTGTTTCTTGTTATGAAACTTTGGT--------

****************************************************

FAD2 TRANSCRIPT TAAAGTTGTCATCAGAGAACTTGAAACTGAC

FAD2 SANGER -------------------------------

**Hydroxysteroid dehydrogenase 1 (HSD1)**

HSD1 TRANSCRIPT GCCATGGGACTTGTTGATTTAATCCACATGTTTCTCAATTTAACTGCCCCCATGTTCACA

HSD1 SANGERS ------------------------------------------ACTGCCCCCATGTTCACA

******************

HSD1 TRANSCRIPT TTCTTCAGCCTACTCCTCATCCTACCTCCATTCTACTTTTTCAAGTCCTTCATGTCAATT

HSD1 SANGERS TTCTTCAGCCTACTCCTCATCCTACCTCCATTCTACTTTTTCAAGTCCTTCATGTCAATT

************************************************************

HSD1 TRANSCRIPT GTAAACACCGTTTTCAGTGAGAACATGGCCGGGAAGGTGGTTCTGATAACCGGGGCATCG

HSD1 SANGERS GTAAACACCGTTTTCAGTGAGAACATGGCCGGGAAGGTGGTTCTGATAACCGGGGCATCG

************************************************************

HSD1 TRANSCRIPT TCGGGGATCGGGGAACATTTGGCGTATGAGTATGCTAGCAGAGGTGCATGTTTGGTTCTT

HSD1 SANGERS TCGGGGATCGGGGAACATTTGGCGTATGAGTATGCTAGCAGAGGTGCATGTTTGGTTCTT

************************************************************

HSD1 TRANSCRIPT TGTGCTAGGAGAGTTGAGAAACTTCATCAAGTTGCTCAGAGGGCTAGGGAGATGGGTGCT

HSD1 SANGERS TGTGCTAGGAGAGTTGAGAAACTTCATCAAGTTGCTCAGAGGGCTAGGGAGATGGGTGCT

************************************************************

HSD1 TRANSCRIPT CTTGATGCTGTTGTCATTCCTACTGATGTTGTCAAAATTGATGATTGTAAAAGGGCTGTT

HSD1 SANGERS CTTGATGCTGTTGTCATTCCTACTGATGTTGTCAAAATTGATGATTGTAAAAGGGCTGTT

************************************************************

HSD1 TRANSCRIPT CAGCAAACGATTCATTATTTTGGGAGATTGGACCACCTTGTAAATAATGCTGGAATCAGT

HSD1 SANGERS CAGCAAACGATTCATTATTTTGGGAGATTGGACCACCTTGTAAATAATGCTGGAATCAGT

************************************************************

HSD1 TRANSCRIPT TCACTTTGCCTGCTTGAAGATGTTGATGATGTAACTGACTTTAGAACGATCATGGACATA

HSD1 SANGERS TCACTTTGCCTGCTTGAAGATGTTGATGATGTAACTGACTTTAGAACGATCATGGACATA

************************************************************

HSD1 TRANSCRIPT AATTTCTGGGGGTCTGTTTACATGACTAGCTGTGCAGCTCCATATCTGAGGCAAAGCAGA

HSD1 SANGERS AATTTCTGGGGGTCTGTTTACATGACTAGCTGTGCAGCTCCATATCTGAGGCAAAGCAGA

************************************************************

HSD1 TRANSCRIPT GGGCGGATAATTGTGCTTTCGTCGTCGGCTTCTTGGTTGCCAGCACCAAGGATGAGCTTC

HSD1 SANGERS GGGCGGATAATTGTGCTTTCGTCGTCGGCTTCTTGGTTGCCAGCACCAAGGATGAGCTTC

************************************************************

HSD1 TRANSCRIPT TATAATGCAAGCAAAGCCGCAATGGCTCAGTTCTTCGAGACTATGAGGGTCGAGTTTGGA

HSD1 SANGERS TATAATGCAAGCAAAGCCGCAATGGCTCAGTTCTTCGAGACTATGAGGGTCGAGTTTGGA

************************************************************

HSD1 TRANSCRIPT CCTGACATTGGAATCACCCTTGTGACTCCGGGCTTTGTAGAGTCCGAACTTACTCAAGGA

HSD1 SANGERS CCTGACATTGGAATCACCCTTGTGACTCCGGGCTTTGTAGAGTCCGAACTTACTCAAGGA

************************************************************

HSD1 TRANSCRIPT AAATTCATAGGCAAGGGAGGGCGCTTGGAAGTTTATCAAGAGATGAGAGATGTGCAAGTT

HSD1 SANGERS AAATTCATAGGCAAGGGAGGGCGCTTGGAAGTTTATCAAGAGATGAGAGATGTGCAAGTT

************************************************************

HSD1 TRANSCRIPT AGTTTGGCCCCAGTAGCAAGAACAGAAAGTTGTGCAAAGGCAATTGTAAGAAGTGCTTGT

HSD1 SANGERS AGTTTGGCCCCAGTAGCAAGAACAGAAAGTTGTGCAAAGGCAATTGTAAGAAGTGCTTGT

************************************************************

HSD1 TRANSCRIPT CGAGGCGAGAGATATCTGACCGAGCCAGCATGGTTCAGAACCACCCTCTGGTGGCACTTG

HSD1 SANGERS CGAGGCGAGAGATATCTGACCGAGCCAGCATGGTTCAGAACCACCCTCTGGTGGCACTTG

************************************************************

HSD1 TRANSCRIPT TTCTGTCCTGAGCTTATGGAGTGGGTGTTCAGAATAATGTACATAACCAATCCCGGGGAG

HSD1 SANGERS TTCTGTCCTGAGCTTATGGAGTGGGTGTTCAGAATAATGTACATAACCAATCCCGGGGAG

************************************************************

HSD1 TRANSCRIPT CCGGCCTCTGAGGCGTGGAGCAAGAAGATATTGGACGTGAGCGGAGCCAAAGCGTTGCTC

HSD1 SANGERS CCGGCCTCTGAGGCGTGGAGCAAGAAGATATTGGACGTGAGCGGAGCCAAAGCGTTGCTC

************************************************************

HSD1 TRANSCRIPT TATCCGGAAACCATGCTATCCGCCGATGTCAAAGCAGAATGATGTGGCTATGAATGTGTT

HSD1 SANGERS TATCCGGAAACCATGCTATCCGCCGATGTCAAAGCAGAATGATGTGGCTATGAATGTGTT

************************************************************

HSD1 TRANSCRIPT ATCTAGTGATAATTTGTGAAGCTGTTTCCTATTGTTAAAAGTGTAATGTGTTTTTGTGTA

HSD1 SANGERS ATCTAGTGATAATTTGTGAAGCTGTTTCCTATTGTTAAAAGTGTAAT-------------

***********************************************

HSD1 TRANSCRIPT AAGAGATTGCAGTCGAGTTGTGTA

HSD1 SANGERS ------------------------

**Caleosin (CAL)**

CAL TRANSCRIPT GGTAGGAGAAGCACCTCTAGCACCAGTGACAGTTGAAAGAAGAGTACGCAATGATTTGGA

CAL SANGER ------------------------------------AAGAAGAGTACGCAATGATTTGGA

************************

CAL TRANSCRIPT GAATACCCTTCCTAAGCCATATATGGCAAGGGGATTGATTGCTCCGGATACTGACCATCC

CAL SANGER GAATACCCTTCCTAAGCCATATATGGCAAGGGGATTGATTGCTCCGGATACTGACCATCC

************************************************************

CAL TRANSCRIPT TACTGGAACACCTGGACACTGGCATGAACATTTGAGTGTACTTCAACAACATGTGGCTTT

CAL SANGER TACTGGAACACCTGGACACTGGCATGAACATTTGAGTGTACTTCAACAACATGTGGCTTT

************************************************************

CAL TRANSCRIPT CTTTGATCAAGATGATAATGGAATTGTCTATCCTTGGGAAACCTATGCTGGATTTCGTCA

CAL SANGER CTTTGATCAAGATGATAATGGAATTGTCTATCCTTGGGAAACCTATGCTGGATTTCGTCA

************************************************************

CAL TRANSCRIPT AGTTGGGTTCAACATGGTAATTTCTCTTTTAGCGGCTATCGTTATCAATGTGGCATTGAG

CAL SANGER AGTTGGGTTCAACATGGTAATTTCTCTTTTAGCGGCTATCGTTATCAATGTGGCATTGAG

************************************************************

CAL TRANSCRIPT TTATCCAACTCTCCCGGGGTGGATACCTTCTCCATTCTTCCCTGTTTACATACTCAACAT

CAL SANGER TTATCCAACTCTCCCGGGGTGGATACCTTCTCCATTCTTCCCTGTTTACATACTCAACAT

************************************************************

CAL TRANSCRIPT ACATAAGAGCAAACATGGTAGTGATACTGGAACTTATGACACCGAGGGAAGGTTCATGCC

CAL SANGER ACATAAGAGCAAACATGGTAGTGATACTGGAACTTATGACACCGAGGGAAGGTTCATGCC

************************************************************

CAL TRANSCRIPT TGTGAACTTCGAGAATACCTTCAGCAAGTATGCGAACACTATGCCCGACAAACTTACACT

CAL SANGER TGTGAACTTCGAGAATACCTTCAGCAAGTATGCGAACACTATGCCCGACAAACTTACACT

************************************************************

CAL TRANSCRIPT TGGAGAACTGTGGGCTATGACAGAAGGAAATCGAGTTTCATTCGATTTTTTTGGATGGCT

CAL SANGER TGGAGAACTGTGGGCTATGACAGAAGGAAATCGAGTTTCATTCGATTTTTTTGGATGGCT

************************************************************

CAL TRANSCRIPT TGCAAGCAAATTCGAGTGGGGAATTCTGTATTTACTAGCAAGAGATGAGGAAGGATTTCT

CAL SANGER TGCAAGCAAATTCGAGTGGGGAATTCTGTATTTACTAGCAAGAGATGAGGAAGGATTTCT

************************************************************

CAL TRANSCRIPT GTCGAAAGAAGCCATCCGACGCTGTTTTGATGGTAGCTTGTTCGAGTATTGTGCCAAGTT

CAL SANGER GTCGAAAGAAGCCATCCGACGCTGTTTTGATGGTAGCTTGTTCGAGTATTGTGCCAAGTT

************************************************************

CAL TRANSCRIPT AAACATGAATGCTGAGGCGAAAATGCAATAAATTGGGTACAAAATAAATCAATTCATCTT

CAL SANGER AAACATGAATGCTGAGGCGAAAATGCAATAAATTGGGTACAAAATAAATCAATTCATCTT

************************************************************

CAL TRANSCRIPT GCAGATGTTTTACATATCTATATAGTACAGAGTACTATTAATAAAGTCTAGTTTGTCTTT

CAL SANGER GCAGATGTTTTACATATCTATATAGTACAGAGTACTATTAATAAAGTCTAGTTTGTCTTT

************************************************************

CAL TRANSCRIPT GAATATTGAAGTCTCGGTTTTGCTTAGTTGAATGTATTGATGCATTAGCTTCTATGTATA

CAL SANGER GAATATTGAAGTCTCGGTTTTGCTTAGTTGAATGTATTGATGCATTAGCTTCTATGTATA

************************************************************

CAL TRANSCRIPT TGAAACTTGGTCTTCGTTCTTGTTAATAAATGCCAAGGAACCAGTG

CAL SANGER TGAAACTTGGTCTTCGTTCTTGTTAATAAATGCCAAGGAAC-----

*****************************************

**Delta-6 desaturase (D6D)**

D6D TRANSCRIPT ATGGCTACTTCTGCAATGAAGAAGTACATTACTGCAGATGAGCTCAAGAATCATGACAAA

D6D SANGER ATGGCTACTTCTGCAATGAAGAAGTACATTACTGCAGATGAGCTCAAGAATCATGACAAA

************************************************************

D6D TRANSCRIPT GAAGGTGATCTCTGGATCTCCATTCAAGGGAAAGTCTATGATGTTTCAGATTGGTTGAAA

D6D SANGER GAAGGTGATCTCTGGATCTCCATTCAAGGGAAAGTCTATGATGTTTCAGATTGGTTGAAA

************************************************************

D6D TRANSCRIPT AACCATCCAGGTGGAAAATTTCCTATAATGAGCCTTGGTGGTCAAGAGGTAACTGATGCA

D6D SANGER AACCATCCAGGTGGAAAATTTCCTATAATGAGCCTTGGTGGTCAAGAGGTAACTGATGCA

************************************************************

D6D TRANSCRIPT TTTGTTGCATTTCATCCTAATTCCACTTGGAAGCTTCTTGAAAGCTTCTTCACTGGTTAT

D6D SANGER TTTGTTGCATTTCATCCTAATTCCACTTGGAAGCTTCTTGAAAGCTTCTTCACTGGTTAT

************************************************************

D6D TRANSCRIPT TATCTTAAAGATTACTCGGTTTCTGAGGTTTCGAAAGATTATAGGAAGCTTGTGTTTGAG

D6D SANGER TATCTTAAAGATTACTCGGTTTCTGAGGTTTCGAAAGATTATAGGAAGCTTGTGTTTGAG

************************************************************

D6D TRANSCRIPT TTTTCTAAAATGGGGTTGTTTGATAAAAAAGGTCATATTGTGTTAGTTACTGTTTTGTTT

D6D SANGER TTTTCTAAAATGGGGTTGTTTGATAAAAAAGGTCATATTGTGTTAGTTACTGTTTTGTTT

************************************************************

D6D TRANSCRIPT ATTATGATGTTGTTTGCTATGAGTGTTTATGGGGTATTGTTTTGTGAGGGTATTTTGGTA

D6D SANGER ATTATGATGTTGTTTGCTATGAGTGTTTATGGGGTATTGTTTTGTGAGGGTATTTTGGTA

************************************************************

D6D TRANSCRIPT CATTTGCTTTCCGGGGGTTTGATGGGGTTTCTTTGGATTCAGAGTGGTTGGATTGGGCAT

D6D SANGER CATTTGCTTTCCGGGGGTTTGATGGGGTTTCTTTGGATTCAGAGTGGTTGGATTGGGCAT

************************************************************

D6D TRANSCRIPT GATGCAGGTCATTATATTGTTATGCCTAATCCGAGGCTTAATAAGGTTATGGGCATTGTT

D6D SANGER GATGCAGGTCATTATATTGTTATGCCTAATCCGAGGCTTAATAAGGTTATGGGCATTGTT

************************************************************

D6D TRANSCRIPT GCTGCGAATTGTCTTGGTGGAATAAGTATTGGTTGGTGGAAATGGAACCACAATGCACAT

D6D SANGER GCTGCGAATTGTCTTGGTGGAATAAGTATTGGTTGGTGGAAATGGAACCACAATGCACAT

************************************************************

D6D TRANSCRIPT CACATTGCCTGTAATAGCCTTGAGTATGACCCGGATTTACAATATATACCGTTTCTTGTT

D6D SANGER CACATTGCCTGTAATAGCCTTGAGTATGACCCGGATTTACAATATATACCGTTTCTTGTT

************************************************************

D6D TRANSCRIPT GTTTCGTCCAAGTTTTTTAGCTCACTCACCTCTCATTTCTATGAGAAAAAATTGACTTTT

D6D SANGER GTTTCGTCCAAGTTTTTTAGCTCACTCACCTCTCATTTCTATGAGAAAAAATTGACTTTT

************************************************************

D6D TRANSCRIPT GACTCTTTATCGAGATTCTTTGTTAGCCATCAACATTGGACATTTTACCCTGTTATGTGT

D6D SANGER GACTCTTTATCGAGATTCTTTGTTAGCCATCAACATTGGACATTTTACCCTGTTATGTGT

************************************************************

D6D TRANSCRIPT GCTGCTAGGCTTAATATGTTTGTACAATCTCTCGTAATGTTGTTGACGAAAAGAAATGTG

D6D SANGER GCTGCTAGGCTTAATATGTTTGTACAATCTCTCGTAATGTTGTTGACGAAAAGAAATGTG

************************************************************

D6D TRANSCRIPT GCATATAGAGGTCAGGAACTCTTGGGATTGTTAGTGTTCTGGATTTGGTACCCGTTGCTC

D6D SANGER GCATATAGAGGTCAGGAACTCTTGGGATTGTTAGTGTTCTGGATTTGGTACCCGTTGCTC

************************************************************

D6D TRANSCRIPT GTTTCGTGTTTGCCTAATTGGGGTGAAAGAATAATGTTTGTCGTTGCTAGCCTCTCCGTG

D6D SANGER GTTTCGTGTTTGCCTAATTGGGGTGAAAGAATAATGTTTGTCGTTGCTAGCCTCTCCGTG

************************************************************

D6D TRANSCRIPT ACTGGACTTCAACAAGTCCAGTTCTCGTTGAACCATTTCTCTTCAAATGTTTATGTTGGA

D6D SANGER ACTGGACTTCAACAAGTCCAGTTCTCGTTGAACCATTTCTCTTCAAATGTTTATGTTGGA

************************************************************

D6D TRANSCRIPT AAGCCTCGAGGGAATGATTGGTTCGAGAAACAAACAGTTGGGACGCTCGACATTTCTTGC

D6D SANGER AAGCCTCGAGGGAATGATTGGTTCGAGAAACAAACAGTTGGGACGCTCGACATTTCTTGC

************************************************************

D6D TRANSCRIPT CCTTCTTGGATGGATTGGTTTCATGGTGGATTGCAATTTCAAGTCGAGCATCATTTGTTC

D6D SANGER CCTTCTTGGATGGATTGGTTTCATGGTGGATTGCAATTTCAAGTCGAGCATCATTTGTTC

************************************************************

D6D TRANSCRIPT CCTAAGCTACCCAGATGCCATCTTAGGAAGATCTCGCCCTTTGTGATGGAGCTATGCAAG

D6D SANGER CCTAAGCTACCCAGATGCCATCTTAGGAAGATCTCGCCCTTTGTGATGGAGCTATGCAAG

************************************************************

D6D TRANSCRIPT AAGCATAACTTGTCCTACAATTATGCCTCATTCTATAAGGCAAATGCAATGACACTCAAA

D6D SANGER AAGCATAACTTGTCCTACAATTATGCCTCATTCTATAAGGCAAATGCAATGACACTCAAA

************************************************************

D6D TRANSCRIPT ACATTAAGGGACACTGCATTGCAAGCTAGGGATCTAACGAAACCGCTCCCAAAGAATTTG

D6D SANGER ACATTAAGGGACACTGCATTGCAAGCTAGGGATCTAACGAAACCGCTCCCAAAGAATTTG

************************************************************

D6D TRANSCRIPT GTATGGGAAGCTCTTCATACTCATGGCTAA

D6D SANGER GTATGGGAAGCTCTTCATACTCATGGTTAA

************************** ***

**Oleosin (OLE)**

OLE TRANSCRIPT ATGGCTATGACTGTTGATCACCACCGCCCACAGCACCTTTCTCACCATGAGACCTCCAGG

OLE SANGER ATGGCTATGACTGTTGATCACCACCGCCCACAACACCTTTCTCACCATGAGACCTCCAGG

******************************** ***************************

OLE TRANSCRIPT AATCCTCTAGCCCACCAGGTGGTGAAAACTGCAACTGCCGTCACAGCTGGTGGTTCACTC

OLE SANGER AATCCTCTAGCCCACCAGGTGGTGAAAACTGCAACTGCCGTCACAGCTGGTGGTTCACTC

************************************************************

OLE TRANSCRIPT CTTGTGGTGTCAGGCCTGACTTTGGCAGCAACTGTGATTGCCCTAACAATGGCCACCCCA

OLE SANGER CTTGTGGTGTCAGGCCTGACTTTGGCAGCAACTGTGATTGCCCTAACAATGGCCACCCCA

************************************************************

OLE TRANSCRIPT TTGCTTGTCCTCTTCAGCCCGGTCCTCGTCCCTGCTGCACTCACCGTCTTCTTGCTTGTC

OLE SANGER TTGCTTGTCCTCTTCAGCCCGGTCCTCGTCCCTGCTGCACTCACCGTCTTCTTGCTTGTC

************************************************************

OLE TRANSCRIPT ACTGGTTTCTTGATTTCGGGCGGCTTTGGGGTGGCTGCCGTGAGTGTCCTGTCGTGGATT

OLE SANGER ACTGGTTTCTTGATTTCGGGCGGCTTTGGGGTGGCTGCCGTGAGTGTCCTGTCGTGGATT

************************************************************

OLE TRANSCRIPT TATCGCTATGTGACAGGAAAGCACCCTGTTGGAGCGGATAGCTTGGATCAGGCGAAGAAT

OLE SANGER TATCGCTATGTGACAGGAAAGCACCCTGTTGGAGCGGATAGCTTGGATCAGGCGAAGAAT

************************************************************

OLE TRANSCRIPT ACCCTGGCGGGGAAGGCTCGGGAGATGAAGGACAAGGCAGAGCAATTCGGGCAGCATAAC

OLE SANGER ACCCTGGCGGGGAAGGCTCGGGAGATGAAGGACAAGGCAGAGCAATTCGGGCAGCATAAC

************************************************************

OLE TRANSCRIPT GTTCAGGGAGGGAACCAGTAA

OLE SANGER GTTCAGGGAGGGAACCAGTAA

*********************
